# Load-dependent RGD-context sensing via αV-class integrins reprograms cell adhesion and mechanics

**DOI:** 10.1038/s41467-026-77028-8

**Published:** 2026-08-24

**Authors:** Upnishad Sharma, Jakob M. Reber, Jonne Helenius, Cara Buchholz, Reinhard Fässler, Nico Strohmeyer, Daniel J. Müller

**Affiliations:** 1 Department of Biosystems Science and Engineering, Swiss Federal Institute of Technology Zurich (ETH), Basel, Switzerland; 2 Department of Molecular Medicine, Max Planck Institute of Biochemistry, Martinsried, Germany

**Keywords:** Integrins, Extracellular matrix, Applications of AFM, Fluorescence imaging, Characterization and analytical techniques

## Abstract

The biochemical and mechanical properties of extracellular matrix proteins govern cell adhesion, mechanics, and migration. How cells use integrins to discriminate between the arginine-glycine-aspartic acid motifs presented by different extracellular matrix proteins, a process central to tissue homeostasis and disease, has remained unclear. Here we show that mammalian cells mount a distinct “biphasic” mechanical response through αV-class integrins to the arginine-glycine-aspartic acid motif of vitronectin compared with fibronectin, osteopontin, and cyclic arginine-glycine-aspartic acid. Within seconds of contact with vitronectin, we find that αV-class integrins strengthen cell adhesion through two load-dependent mechanotransduction pathways in which αVβ3 and αVβ5 integrins take complementary roles. Under low load, we demonstrate that the first phase requires both integrins together with an intact, pre-tensed actomyosin cortex, talin, paxillin, and focal adhesion kinase activity, with αVβ5 integrin additionally engaging clathrin-mediated endocytosis. Under higher load, we show that the second phase is dominated by αVβ3 integrin–directed actin-related protein 2/3, cellular Src kinase, and phosphatidyl inositol-3-kinase signaling, which organizes the consensus adhesome, while αVβ5 integrin concurrently drives cellular stiffening. Taken together, we find that αV-class integrins rapidly deploy arginine-glycine-aspartic acid -motif- and β-subunit-specific programs that cooperatively tune cell adhesion and mechanics according to the extracellular matrix composition.

## Abstract (web summary)

Here the authors show that αV-class integrins distinguish the RGD motif of vitronectin from other ligands and, within seconds, deploy β-subunit-specific, load-dependent programs in which αVβ3 and αVβ5 cooperate to tune fibroblast adhesion and mechanics to ECM composition.

## Introduction

Mammalian cells adhere to the extracellular matrix (ECM) using highly dynamic and hierarchical integrin-based adhesion complexes that establish an ECM-receptor-adapter-cytoskeleton axis^1–4^. Integrins are obligatory heterodimeric transmembrane receptors that enable adherent cells to sense and interpret biochemical, structural and mechanical cues in their immediate environment^5,6^. Different classes of integrins adhere to diverse ECM proteins like fibronectin, osteopontin, or vitronectin to assemble adhesion complexes^7–10^. Such integrin-ECM interactions are critical for biological processes such as wound healing and development^11–14^. α5β1, α8β1 and the αV-class (αVβ1, αVβ3, αVβ5, αVβ6, and αVβ8) integrins bind to the arginine-glycine-aspartic acid (RGD) motif of ECM proteins, govern early morphogenesis^15^, osteogenesis, lipid biogenesis^16^ and are involved in several disease conditions, including fibrosis^17^, tumor metastasis, and age-related macular degeneration^10,18–24^. Although all αV-class integrins bind RGD-containing ECM ligands, the individual members have evolved specialized roles - TGFβ activation for αVβ6 and αVβ8 integrins^17,25^; pro-survival and pro-angiogenic signaling for αVβ3 integrin^24,26,27^; and novel adhesion assemblies for αVβ5 integrin^28–32^. Thereby, the RGD-motif in ECM proteins, such as presented in non-structured regions in vitronectin and osteopontin, or in structured domains in fibronectin, triggers specific phenotypic responses of cellular systems^33^. In recent years, elucidating the interaction of integrins with vitronectin has attracted substantial interest across cell biology, mechanobiology, biomaterials science, and a wide range of medical applications^29,34–36^. However, the mechanisms by which cells employ integrins to recognize the RGD-motif within vitronectin, discriminate it from RGD-motifs in other ECM proteins, and subsequently regulate adhesion remain poorly understood.

The involvement of αV-class integrins in diverse biological processes coincides with their exposure to a wide variety of mechanical cues like variable intra- or extracellular tensile forces that influence the assembly and dynamics of adhesion complexes^1,11,14,37–43^. Upon ligand binding, αV-class integrins assemble nascent adhesions that mature into adhesion complexes as the actomyosin-mediated contractile forces increase, on timescales of minutes to hours^4,38,44,45^. How rapidly αV-class integrins recognize the RGD-motif of the key ECM proteins, fibronectin and vitronectin, and how rapidly cells at this onset of adhesion respond and make decisions that affect cell adhesion on the long time scale could not be addressed, mainly due to the limited temporal and force resolution of the biophysical techniques applied^1,33,46,47^. Moreover, while mechanotransduction of integrins binding to fibronectin is well studied, it is debated whether cell adhesion to vitronectin is mechanosensitive^45,48–52^, although it was shown recently that force-mediated structural changes in the hybrid domain of integrin αVβ3 are essential for its differential binding to fibronectin and vitronectin^33,53^. The way αV-class integrins relay this contextualization of the RGD-motif under varying mechanical load, such as experienced within tissues, also demands inquiry. Although the effects of cell-generated tensile and traction forces on αV-class and β1-class integrins have been studied from a few minutes to several hours^33,54^, the effect of force-loading during early cell adhesion (≤120 s) when cells make decisions, to vitronectin, remains elusive. Thus, exploring the early time of adhesion initiation and strengthening is fundamental to understanding how cells employ integrins to mechanosense the ECM and initiate cellular responses.

Here, we explore how αV-class integrins initiate and mechanically strengthen cell adhesion to RGD-motifs in various substrates. Applying an interdisciplinary approach that includes atomic force microscopy (AFM)-based single-cell force spectroscopy (SCFS), mass spectrometry-based adhesion proteomics, chemical perturbations, three-dimensional structured illumination microscopy (3D SIM), and fluorescence microscopy, we characterize the early and late adhesion of genetically engineered mouse embryonic fibroblasts. We find that two prominent members of αV-class integrins, αVβ3 and αVβ5 integrin, distinguish the different RGD-context in fibronectin, osteopontin, cyclic-RGD, and vitronectin in seconds and that this contextualization leads fibroblasts to strengthen individual integrin-bonds as well as cell adhesion monophasically or biphasically in response to varying mechanical load. Thereby, αV-class integrins facilitate the unique mechanosensitive biphasic adhesion response of fibroblasts only to vitronectin and through two different integrin β-subunits, β3 and β5. Each of these integrin subunits uses specific components of the actomyosin cytoskeleton and employs different biochemical signaling pathways to establish substrate-specific cell adhesion and mechanical responses. Furthermore, we observe that, depending on the mechanical load, the signaling pathway required by either one of the two αV-class integrins dominates the other.

## Results

### αV-class integrins distinguish context of RGD-motif under mechanical load

**Fig. 1:**
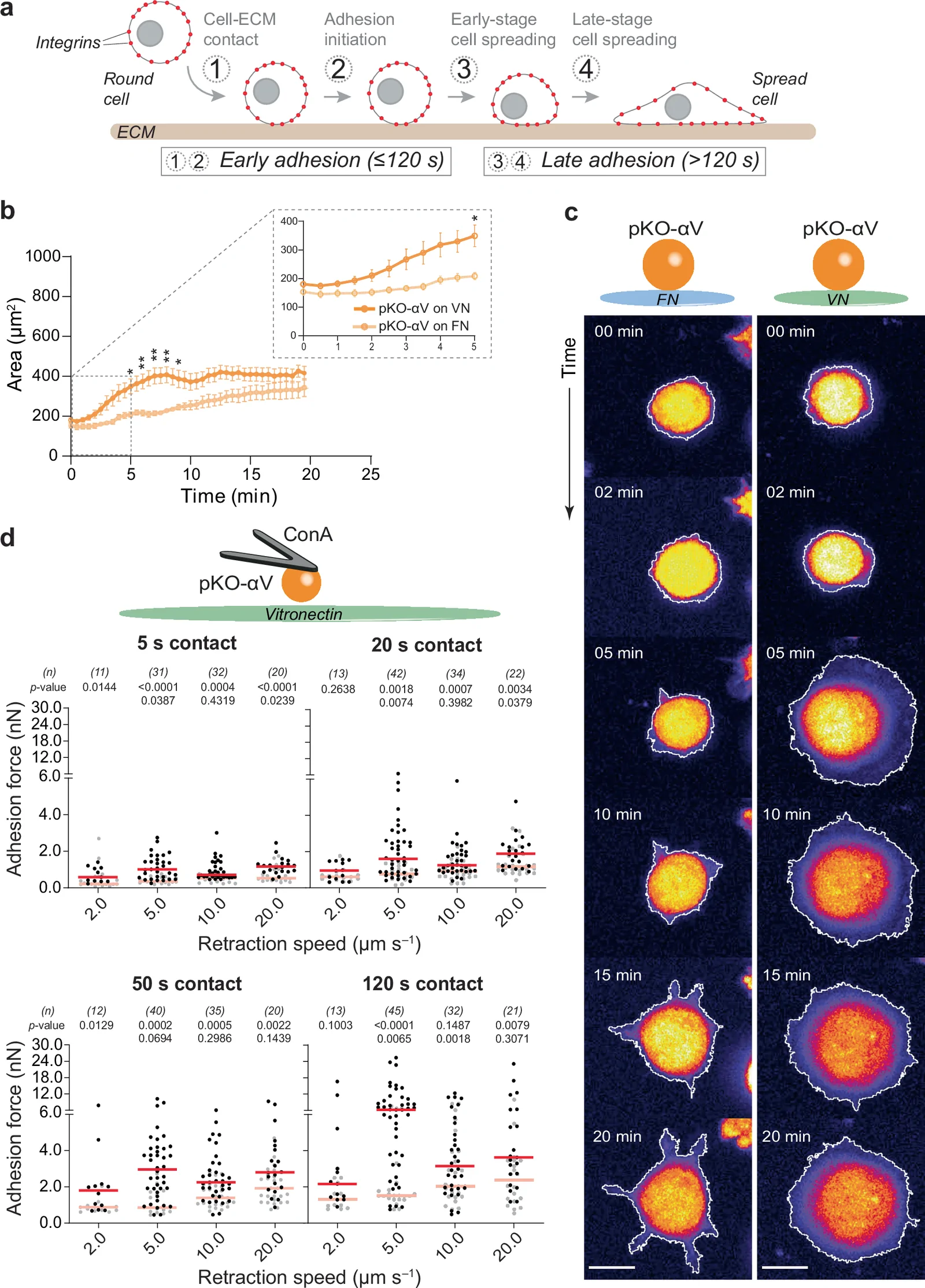
Within seconds of contact to vitronectin αV-class integrins distinguish RGD context under mechanical load. **a** Schematic key changes a cell undergoes as it transitions from the round to the spread state on an ECM-coated substrate. Cell-ECM contact (1) is followed by adhesion initiation (2); these together comprise early adhesion (≤120 s). Thereafter (>120 s), the early-stage cell spreading (3) results in cell flattening, with the late-stage cell spreading being accompanied by the formation of integrin-based adhesion complexes (4). **b** Spreading kinetics of pKO-αV fibroblasts on fibronectin (FN) (*n* = 12) or vitronectin (VN) (*n* = 16) following their first contact with the substrate. Confocal microscopy images were acquired every 30 s. The inset shows the spreading dynamics of fibroblasts on vitronectin and fibronectin within the first 5 min after attachment in better detail. Each data point presents the mean ± SE from > 10 fibroblasts. *P*-value at 5 min (* = 0.0102); 6 min (** = 0.0037); 7 min (** = 0.0031); 8 min (** = 0.0062); 9 min (* = 0.0374), obtained using two-tailed Mann–Whitney U-test. **c** Time-stamped confo**c**al microscopy of pKO-αV fibroblasts stained with CellTracker™ dye (shown in pseudocolor) and spreading on fibronectin or vitronectin. White outlines represent the masks used for calculating the areas used in (**b**). Scale bars, 10 µm. **d** Adhesion force of pKO-αV fibroblasts to vitronectin-coated glass increases ‘biphasically’ with retraction speed and contact time. The biphasic increase in adhesion force becomes most prominent at 120 s contact time. Each dot represents the measurement of a single fibroblast; red bars show the median. *P*-values were obtained using a two-tailed Mann-Whitney U-test. *P*-values in the top row compare cell adhesion force on vitronectin (black) to fibronectin (lightened). *P*-values in the bottom row compare data for a given retraction speed to that of the preceding retraction speed (*e.g*., 5 µm s^–1^ with 2 µm s^–1^). *n* denotes the number of fibroblasts characterized on three different experimental days.

To understand whether αV-class integrins respond to the RGD-motif context, we used mouse embryonic fibroblasts (pKO-αV, pan integrin knockout cells with β2^−/−^, β7^−/−^, β1^−/−^, αV^−/−^, αV reintroduced) expressing two αV-class integrins, αVβ3 and αVβ5, at similar levels on the cell surface^45^. To characterize the effect of the RGD-context sensing during early stages of cell spreading, we performed time-lapse confocal microscopy of pKO-αV fibroblasts adhering to vitronectin or fibronectin within 20 min (Fig. 1a–c). On vitronectin, pKO-αV fibroblasts spread over larger areas (~400 µm^2^) and faster (within 10 min at a rate of 36.45 µm^2^ min^–1^) than on fibronectin (~350 µm^2^, within 20 min at 2.46 µm^2^ min^–1^). To further characterize the protein composition of the adhesion plaques (adhesomes) of pKO-αV fibroblasts cultured on vitronectin- or fibronectin-substrates for longer times of 45 min, we conducted mass spectrometry, which revealed substrate-dependent differences (Supplementary Fig. 1). On fibronectin, pKO-αV fibroblasts enriched different collagens (Col1a1, Col1a2, Col3a1, Col12a1, Col18a1, etc.), whereas cultured on vitronectin they enriched components of the complement system (C3, C4b, C8b, C8g, Cfb, etc.). The uniqueness of these peptides against the source peptides used to culture the cells was further confirmed by sequence analysis (Supplementary Table 1). Taken together, these differences suggest that within longer adhesion times ≥ 20 min, fibroblasts differentially respond to the RGD-motif context of vitronectin and fibronectin.

We next characterized, whether this RGD-contextualization of ECM proteins by αV-class integrin initiates already at earlier (≤120 s) phases when fibroblasts establish adhesion and are exposed to mechanical load, such as occurring in tissues^39,55,56^. Utilizing AFM-based SCFS^57–60^ (Supplementary Fig. 2a–c), we quantified the adhesion force of pKO-αV fibroblasts to fibronectin- or vitronectin-coated substrates for contact times between 5–120 s, by detaching them at different retraction speeds of 2 to 20 µm s^–1^ (ref. ^48,61^), which simulated mechanical loading rates comparable to those occurring in tissues, such as interstitial fluid flows^55^. With vitronectin, we observed a rapid increase in cell adhesion force between 2 and 5 µm s^–1^ (Fig. 1d). At 10 µm s^–1^, however, the adhesion force reduced and thereafter increased more gradually until 20 µm s^–1^. A detailed insight into this non-linear cell adhesion response was achieved by probing the retraction speed regime of 3, 6 and 8 µm s^–1^ (Supplementary Fig. 3a). We termed this behavior of steeply increasing the cell adhesion force up to a certain speed (loading rate), above which the adhesion force abruptly decreases before it gradually increases again at higher speeds, the ‘biphasic adhesion response’ of the cell to mechanical load. Markedly, pKO-αV fibroblasts showed a monophasic adhesion response to fibronectin with increasing retraction speeds (Supplementary Fig. 3b).

The biphasic adhesion response of pKO-αV fibroblasts becomes more prominent upon increasing the contact time to vitronectin to 120 s, and the cell adhesion maximizes at a retraction speed of 5 µm s^–1^ (Fig. 1d). To further test which cellular mechanisms contribute to regulating this response, we examined the adhesion response of fibroblasts to mechanical load at contact times of 120 s. First, we used the assay to test whether other ligands of αV-class integrins that carry the RGD-motif, osteopontin (Supplementary Fig. 4a) or cRGD [cyclo-RGDfV pentapeptide] (Supplementary Fig. 4b), caused a biphasic adhesion response of pKO-αV fibroblasts, which they did not. Together, these experiments showed that the biphasic adhesion response of fibroblasts to mechanical load was specific to the RGD-motif in vitronectin.

### Integrin β-subunits dictate biphasic cell adhesion response

**Fig. 2:**
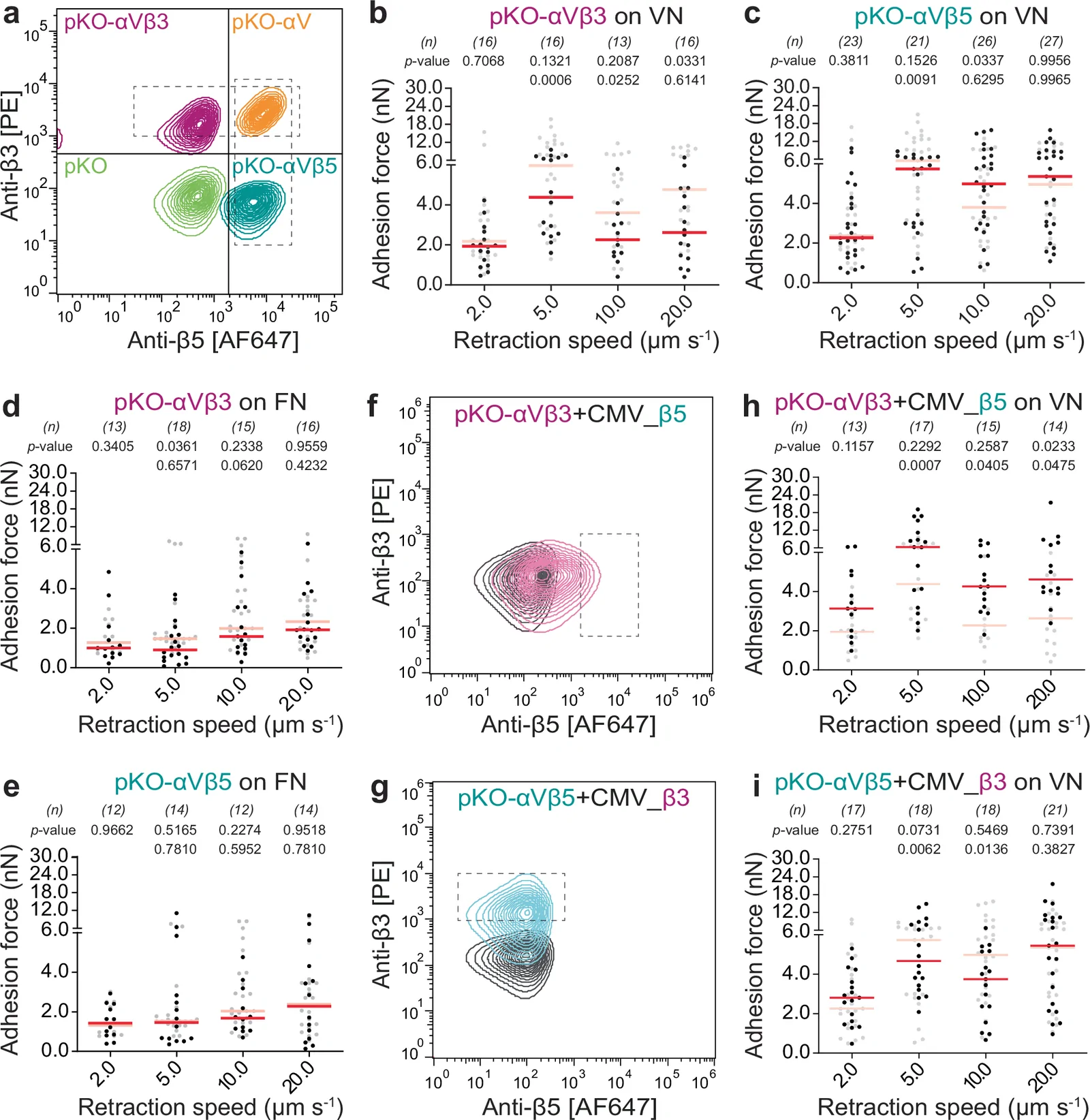
β-subunits of αV-class integrin liganded to vitronectin cause fibroblasts to strengthen adhesion biphasically in response to mechanical load. **a** Flow cytometry analysis of pKO, pKO-αVβ5, pKO-αV, and pKO-αVβ3 fibroblasts (“Methods”). **b**–**e** Adhesion force of pKO-αVβ3 and pKO-αVβ5 fibroblasts to vitronectin (**b**, **c**) and to fibronectin (**d**, **e**) with increasing retraction speed. **b** Biphasic adhesion response of pKO-αVβ3 fibroblasts to vitronectin appears like pKO-αV fibroblasts (lightened data) but shows lower force. **c** Biphasic adhesion response of pKO-αVβ5 fibroblasts to vitronectin is unlike pKO-αV fibroblasts (lightened data). The lightened data in (**b**, **c**) compare the adhesion force of pKO-αV fibroblasts to vitronectin (Fig. 1d). **d** pKO-αVβ3 fibroblasts adhering to fibronectin show a monophasic adhesion response with increasing retraction speed, like pKO-αV fibroblasts (lightened data). **e** pKO-αVβ5 fibroblasts adhering to fibronectin show a monophasic adhesion response with increasing retraction speed, like pKO-αV fibroblasts (lightened data). **f** Flow cytometry sorting scheme confirming CMV_β5 rescued pKO-αVβ3 fibroblasts (pink) against isotype control (gray). **g** Flow cytometry sorting scheme to confirm CMV_β3 rescued pKO-αVβ5 fibroblasts (blue) against isotype control (gray). The gray box in f and g represents fibroblasts sorted for similar surface integrin expression to pKO-αV fibroblasts, subsequently used for further experimentation. **h** pKO-αVβ3 fibroblasts rescued for integrin β5 expression (dark red bar, black dots) show increased adhesion force with increasing retraction speed versus non-rescued pKO-αVβ3 fibroblasts (lightened data). **i** pKO-αVβ5 fibroblasts rescued for integrin β3 expression (dark red bar, black dots) show a restored biphasic increase of adhesion force with increasing retraction speed versus non-rescued pKO-αVβ5 fibroblasts (lightened data). SCFS data in **b**–**e**, **h**, and **i** were recorded after 120 s on the substrate. Red bars in **b**–**e**, **h**, and **i** represent the median. *P*-values were obtained using a two-tailed Mann-Whitney U-test. *P*-values in the top row compare the data to the control (lightened) data. *P*-values in the bottom row compare data for a given retraction speed with the preceding retraction speed (e.g., 5 µm s^–1^ with 2 µm s^–1^). *n* denotes the number of fibroblasts measured.

The presence of αVβ3 and αVβ5 integrins in pKO-αV fibroblasts hinders the assignment of the vitronectin-specific biphasic adhesion response to either of the two heterodimers. To delineate the contributions of αVβ3 and αVβ5 integrins, we performed a CRISPR/Cas9-mediated deletion of the gene encoding the integrin β3 (*Itgb3*)- or β5 (*Itgb5*)-subunit in pKO-αV fibroblasts, resulting in pKO-αVβ5 and pKO-αVβ3 fibroblasts, respectively (Fig. 2a). We sorted pKO-αVβ3 and pKO-αVβ5 fibroblasts using flow cytometry to obtain cells with integrin expression-levels similar to pKO-αV fibroblasts (Fig. 2a). As quantified by 3D SIM, the cell surface density of αV-class integrins was comparable for pKO-αV, pKO-αVβ3, and pKO-αVβ5 fibroblasts (Supplementary Fig. 5). These fibroblasts were then subjected to SCFS as described above to quantify their early adhesion to vitronectin or fibronectin. To vitronectin, pKO-αVβ3 fibroblasts rapidly increased adhesion force upon increasing the retraction speed from 2 to 5 µm s^–1^, though with lower force compared to pKO-αV fibroblasts (Fig. 2b). The adhesion force considerably reduced at 10 µm s^–1^ retraction speed and remained low at 20 µm s^–1^. In contrast, pKO-αVβ5 fibroblasts increased the adhesion force to vitronectin with increasing the retraction speed from 2 to 5 µm s^–1^ and plateaued with high adhesion force at higher retraction speeds (Fig. 2c). This suggests that αVβ3 and αVβ5 integrins differentially contribute to the biphasic cell adhesion response to mechanical load. Moreover, this biphasic adhesion response, which is mediated through αVβ3 and αVβ5 integrins, is unique to vitronectin (Fig. 2d, e). Furthermore, upon constitutively activating pKO-αVβ3 or pKO-αVβ5-fibroblasts using manganese, their biphasic adhesion response disappeared (Supplementary Fig. 6), which suggests that integrin β3- and β5-subunits must be mechanically activated to mediate the biphasic adhesion response^62^.

### Individual integrin subunits govern biphasic adhesion response

To explore how integrin β3- and β5-subunits facilitate the biphasic adhesion response of fibroblasts, we restored their surface expression in pKO-αVβ5 and pKO-αVβ3 fibroblasts, respectively (Fig. 2f, g). Upon rescuing the respective integrin β-subunit, we found the biphasic adhesion response of pKO-αVβ3 and pKO-αVβ5 fibroblasts became similar to that of pKO-αV fibroblasts (Fig. 2h, i). Interestingly, rescuing the pKO-αVβ3 fibroblasts with the β5-subunit led to a higher cell adhesion force, especially at higher retraction speeds (Fig. 2h). As expected, rescuing the β3-subunit in pKO-αVβ5 fibroblasts considerably lowered the cell adhesion force at 10 µm s^–1^ compared to 5 µm s^–1^ (Fig. 2i). Complementary to the genetic manipulation, the inhibition of the integrin β3-subunit in pKO-αV fibroblasts by P11, a non-RGD inhibitor of αVβ3 integrin with the sequence HSDVHK^63,64^, an antagonist specific to αVβ3 integrin but not to αVβ5 integrin^65^, corroborated the contribution of the β5-subunit to the biphasic cell adhesion response, though at lower overall adhesion force (Supplementary Fig. 7).

Additionally, the data suggest that the αV-subunit, being the only unaltered integrin subunit among the fibroblasts, dominates the considerable increase in cell adhesion force between 2 µm s^–1^ and 5 µm s^–1^ (Fig. 1d, Supplementary Fig. 3a and Fig. 2h, i). Based on this observation, we infer that the rapid increase in cell adhesion force between 2 µm s^–1^ and 5 µm s^–1^ was conserved for pKO-αV, pKO-αVβ3, and pKO-αVβ5 fibroblasts, despite their different integrin β-subunit compositions. Because the cell adhesion force between 5 µm s^–1^ and 10 µm s^–1^ plateaued for αVβ5 integrin and decreased for αVβ3 integrin, the biphasic adhesion response critically depends on the β-subunit of the heterodimer. The repression and rescue of the β-subunit expression in various αV-class integrin-expressing fibroblasts helped us delineate the potential role of individual integrin α- and β-subunits in responding to mechanical load, which is further addressed in the following.

### β3- and β5-subunits modulate integrin-vitronectin bonds

Next, we tested whether the cell adhesion response to varying mechanical load (retraction speed) arises from single integrin-vitronectin (receptor-ligand) bond characteristics. We measured the binding probability of pKO-αV, pKO-αVβ3, and pKO-αVβ5 fibroblasts to vitronectin by detaching individual fibroblasts after ~50 ms interaction with vitronectin (“Methods”, Supplementary Fig. 8)^59,60,66^. αVβ5 integrin in pKO-αVβ5 fibroblasts showed the highest binding probability to vitronectin of 0.76 ± 0.03 (mean ± SE), αVβ3 integrin in pKO-αVβ3 fibroblasts showed vitronectin-binding probabilities of 0.68 ± 0.02, and both integrins in pKO-αV fibroblasts showed the lowest vitronectin-binding probability of 0.59 ± 0.03. Because αVβ3 and αVβ5 integrins individually show higher binding probabilities than together, this indicates that both αV-class integrins influence each other in establishing adhesion to vitronectin.

Further, we analyzed the rupture force of single integrin-vitronectin bonds, which were also recorded by SCFS upon detaching fibroblasts from vitronectin (Supplementary Fig. 9). The rupture force of single vitronectin-bound αVβ3 integrins in pKO-αVβ3 fibroblasts increased non-linearly with the mechanical load (Supplementary Fig. 9a, b). This non-linear dependence for αV-class integrin bound to vitronectin parallels the previously reported speed-dependent rupture forces of α5β1 integrin bound to fibronectin^48,61^. Because the loading rate experienced at a given retraction speed is co-determined by the adhesion stiffness of the cell, which differs between conditions, we describe this dependence empirically and do not interpret it in terms of single-bond kinetics. The higher rupture force of integrins in pKO-αV and pKO-αVβ5 fibroblasts and the overall lower rupture force of integrins in pKO-αVβ3 fibroblasts recapitulated the overall behavior of cell adhesion force (Supplementary Fig. 9 and Fig. 2b, c). However, the rupture force of integrins in pKO-αV fibroblasts, expressing αVβ3 and αVβ5 integrins, was between that of pKO-αVβ3 fibroblasts expressing αVβ3 integrin and pKO-αVβ5 fibroblasts expressing αVβ5 integrin (Supplementary Fig. 9). Moreover, the loading rate experienced by each integrin-vitronectin bond before rupturing depends on the retraction speed and differs for αVβ3 and αVβ5 integrins (Supplementary Fig. 10). This indicates that in pKO-αV fibroblasts αVβ3 and αVβ5 integrins act synergistically^67^ when experiencing mechanical load upon adhering to vitronectin for 120 s. However, the non-linear rupture force of integrins in pKO-αV fibroblasts in response to increasing retraction speed was more similar to that of αVβ3 integrin from pKO-αVβ3 fibroblasts compared to αVβ5 integrin from pKO-αVβ5 fibroblasts, which indicates that αVβ3 integrin dominates αVβ5 integrin if both are co-expressed. This dominance of αVβ3 over αVβ5 integrin for the binding of vitronectin is particularly interesting, since αVβ5 integrin establishes higher ligand-unbinding forces compared to αVβ3 integrin (Supplementary Fig. 9). Thus, while both αVβ3 and αVβ5 integrins facilitate the non-linear adhesion response of vitronectin-bound αV-class integrins, αVβ3 integrin dominates the binding behavior over αVβ5 integrin, although it is αVβ5 integrin that contributes most to the higher cell adhesion force to vitronectin (Fig. 2h).

### Biphasic cell adhesion response requires actomyosin cortex regulation

To explore the roles of the intracellular components of the ECM-integrin-adapter-cytoskeleton axis on the biphasic cell adhesion response, we characterized the involvement of structural and regulatory components of the actomyosin cortex, including F-actin, myosin II, Rho-associated coiled-coil containing protein kinase (ROCK), Rho-effector formin protein mDia, and the actin-related protein 2/3 (Arp2/3) complex. To this end, we used small molecule inhibitors such as latrunculin A to perturb F-actin, blebbistatin to perturb myosin II, Y27632 to perturb ROCK, SMIFH2 to perturb mDia, or CK666 to perturb Arp2/3. The inhibition of these actomyosin components or regulators did not change the cell surface expression levels of αVβ3 and αVβ5 integrins (Supplementary Fig. 11). Disrupting F-actin formation by latrunculin A considerably reduced the cell adhesion force and led to a loss of the biphasic adhesion response in pKO-αV, pKO-αVβ3, and pKO-αVβ5 fibroblasts (Supplementary Fig. 12a). Perturbing intracellular force generation by inhibiting actomyosin contractility, which is essential for intracellular force generation at adhesion sites, through the inhibition of myosin II and ROCK activity^11^, reduced the adhesion force exclusively at 5 µm s^–1^ and removed the biphasic adhesion response (Supplementary Fig. 12b, c). The cell adhesion force for all other retraction speeds remained unchanged. Thus, myosin II and ROCK activity contribute to the biphasic adhesion response of pKO-αV, pKO-αVβ3, and pKO-αVβ5 fibroblasts but not to the overall cell adhesion force at high loading rates.

**Fig. 3:**
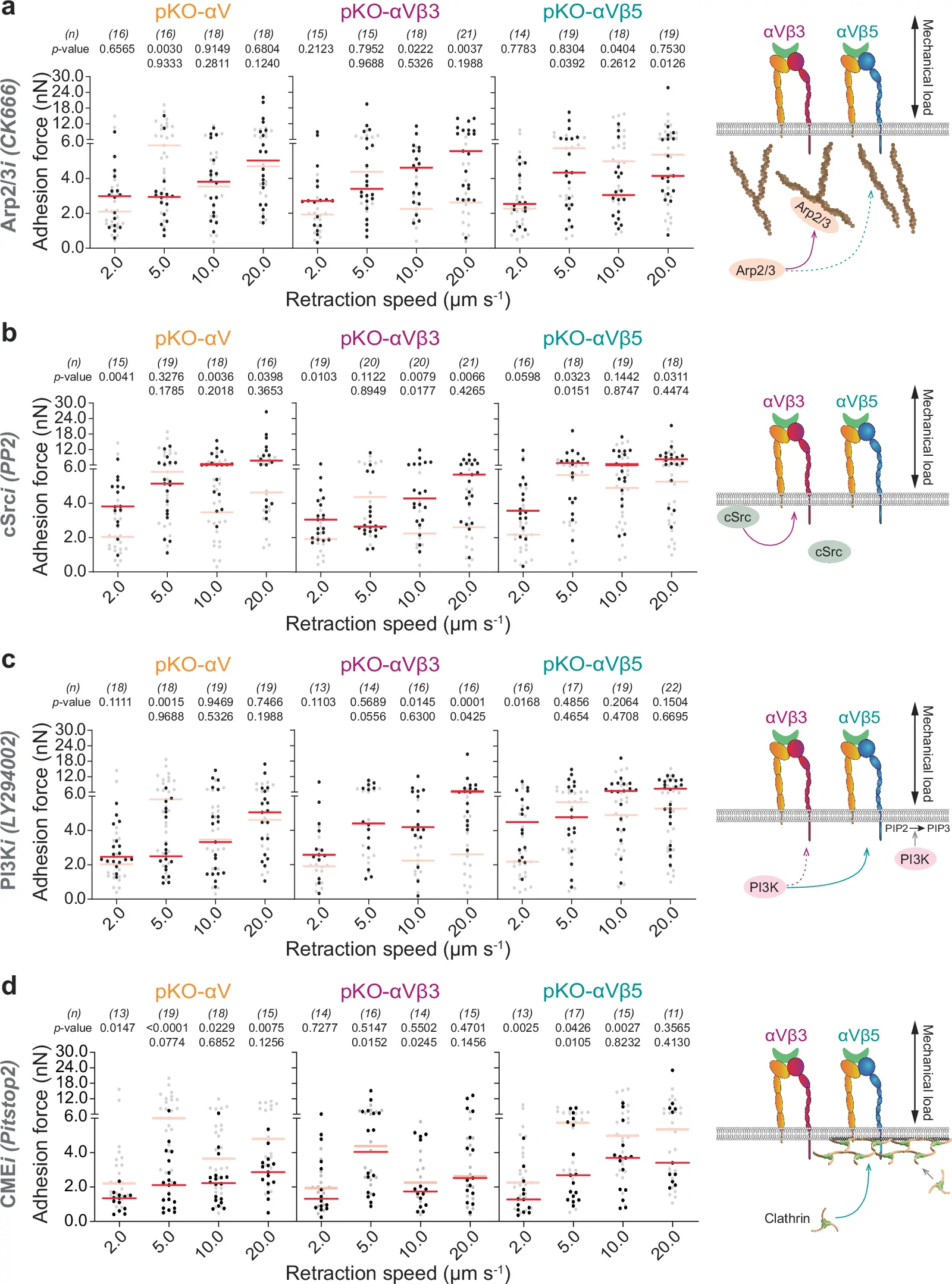
Arp2/3, cSrc, PI3K, and clathrin-mediated endocytosis differentially mediate biphasic adhesion response to mechanical load in an integrin β-subunit-specific manner. **a**–**d** Retraction speed-dependent adhesion force of chemically perturbed pKO-αV, pKO-αVβ3 and pKO-αVβ5 fibroblasts on vitronectin substrate after 120 s of contact. Fibroblasts were treated with **a** 200 μM Arp2/3 inhibitor CK666, **b** 20 μM cSrc kinase inhibitor PP2, **c** 10 μM phosphatidyl inositol 3-kinase (PI3K) inhibitor LY294002, and **d** 30 μM clathrin-mediated endocytosis (CME) inhibitor Pitstop2. Schematics on the right in each panel highlight the differential involvement of the perturbed signaling pathway to αVβ3 or αVβ5 integrin under mechanical load. Each dot in the SCFS data corresponds to one fibroblast, red bars to the median. *P*-values were obtained using a two-tailed Mann–Whitney U-test. *n* denotes the number of cells measured. *P*-values in the top row compare the data to the unperturbed (lightened) data. *P*-values in the bottom row compare data for a given retraction speed to the preceding speed (e.g., 5 µm s^–1^ with respect to 2 µm s^–1^).

Disrupting actin nucleation by inhibiting the downstream Rho-effector and formin protein mDia diminished the biphasic adhesion response in pKO-αV, pKO-αVβ3, and pKO-αVβ5 fibroblasts (Supplementary Fig. 12d). Additionally, it considerably reduced the adhesion force for all retraction speeds in pKO-αV and pKO-αVβ5 fibroblasts. Lastly, prevention of cortical actin branching by Arp2/3 inhibition disrupted the biphasic adhesion response for all three fibroblast lines (Fig. 3a). The rapid increase in adhesion force between 2 and 5 µm s^–1^ (low retraction speeds) largely reduced in both pKO-αV and pKO-αVβ3 fibroblasts, while in pKO-αVβ5 fibroblasts this rapid increase in adhesion force remained unchanged. However, for 10 and 20 µm s^–1^ (high retraction speeds), pKO-αVβ3 fibroblasts increased the adhesion force, while pKO-αVβ5 fibroblasts reduced it. This opposite effect of Arp2/3 inhibition on pKO-αVβ3 and pKO-αVβ5 fibroblast adhesion indicates a differential engagement of Arp2/3 activity in an integrin β-subunit-specific manner.

Together, actomyosin structural integrity (F-actin), nucleation (mDia), and contractility (myosin II and ROCK) are essential for vitronectin-interacting fibroblasts to initiate the biphasic adhesion response to mechanical load. The actomyosin contractility is required for the rapid increase in cell adhesion at low retraction speeds by both αVβ3 and αVβ5 integrins, which marks the first phase of the biphasic cell adhesion response. The inhibition of the actin branching Arp2/3 complex led pKO-αVβ3 fibroblasts to establish higher adhesion force and pKO-αVβ5 fibroblasts to establish lower adhesion force at higher retraction speeds. This observation suggests that the Arp2/3 complex is required differentially to strengthen adhesion by αVβ3 and αVβ5 integrins under mechanical load.

### β-subunit drives mechanotransduction of vitronectin-bound αV-class integrins

After characterizing the role of the actomyosin cortex and its regulation in the vitronectin-bound αV-class integrin-mediated biphasic adhesion response, we explored the involvement of key signaling proteins, including the cellular Src kinase (cSrc)^24,68^, phosphatidyl inositol 3-kinase (PI3K), and focal adhesion kinase (FAK)^40,69^. To perturb these signaling proteins, we used small molecules PP2 to inhibit cSrc, LY294002 to inhibit PI3K, or Y11 and PF573228 to inhibit FAK, which did not change the surface expression of αVβ3 or αVβ5 integrins (Supplementary Fig. 11). cSrc inhibition disrupted the biphasic adhesion response of pKO-αV and pKO-αVβ3 fibroblasts, while it did not affect the biphasic adhesion response of pKO-αVβ5 fibroblasts (Fig. 3b). FAK inhibition reduced the adhesion force of pKO-αV, pKO-αVβ3 and pKO-αVβ5 fibroblasts at 5 µm s^–1^ (Supplementary Fig. 12e, f). Thus, upon FAK inhibition, all fibroblasts lost their ability to establish the biphasic adhesion response.

To test the contributions of membrane phospholipids, we inhibited PI3K to prevent the formation of phosphatidyl inositol 3,4,5-trisphosphate (PIP3) from phosphatidyl inositol 4,5-bisphosphate (PIP2) (Fig. 3c). In pKO-αV fibroblasts, PI3K-inhibition disrupted the biphasic adhesion response by considerably reducing the adhesion force at 5 µm s^–1^ retraction speed, while maintaining the adhesion force at 2, 10, and 20 µm s^–1^. pKO-αVβ3 fibroblasts responded to PI3K-inhibition by reducing the biphasic adhesion response; however, the fibroblasts increased adhesion force considerably at 10 and 20 µm s^–1^. pKO-αVβ5 fibroblasts increased the adhesion force at 2 µm s^–1^, which disrupted the biphasic adhesion response, while the adhesion force at 5, 10, and 20 µm s^–1^ retraction speeds did not change. These findings highlight that FAK and PI3K are essential for the early cell adhesion response to mechanical load for both integrin β-subunits, while αVβ3 integrins preferably required cSrc signaling.

Because recent reports have linked αVβ5 integrin to clathrin-mediated endocytosis (CME)^70^ and flat clathrin lattices^32^, we queried the involvement of this endocytosis in the early adhesion-related biphasic adhesion response. Upon inhibiting CME by Pitstop2, the biphasic adhesion response was lost for αVβ5 integrin-expressing pKO-αV and pKO-αVβ5 fibroblasts (Fig. 3d). In contrast, pKO-αVβ3 fibroblasts maintained the biphasic adhesion response. Hence, the signaling pathways involved in the sensing of mechanical load through vitronectin-bound αV-class integrins differ for αVβ3 and αVβ5 integrins.

To better understand the role of integrin β3/β5-subunits in mechanotransduction, we transfected the pKO-αVβ3 fibroblasts with a chimeric β-subunit that contained the extracellular and transmembrane domain of the integrin β5-subunit fused to the cytoplasmic tail of the integrin β3-subunit^71^. pKO-αVβ3 fibroblasts transfected with this chimeric β-subunit could not entirely recover the biphasic adhesion response, as observed for pKO-αV fibroblasts, unlike pKO-αVβ3 fibroblasts rescued with the full integrin β5-subunit (Supplementary Fig. 13a and Fig. 2h). The biphasic adhesion response of pKO-αVβ3 fibroblasts transfected with the chimeric β-subunit instead remained unchanged (Fig. 2b). We further confirmed the involvement of the integrin β-subunit tail in the biphasic adhesion response by depleting the adapter proteins paxillin and talin (Supplementary Fig. 13b, c). The depletion of paxillin (PxnKO) disrupted the biphasic adhesion response for both pKO-αV and pKO-αVβ5 fibroblasts. Interestingly, pKO-αVβ5 (PxnKO) fibroblasts considerably reduced the adhesion force for all retraction speeds, unlike pKO-αV (PxnKO) fibroblasts. Upon depletion of talin in pKO-αV fibroblasts, the biphasic adhesion response disappeared along with a reduction of adhesion force for all retraction speeds (Supplementary Fig. 13d, e). Together, the data indicate the involvement of the integrin β5-subunit tail, and the co-existence of different mechanosensing signaling pathways that are employed by the integrin β3- and β5-subunits, during the binding to vitronectin.

### Vitronectin-bound αV-class integrins modulate cellular stiffness

**Fig. 4:**
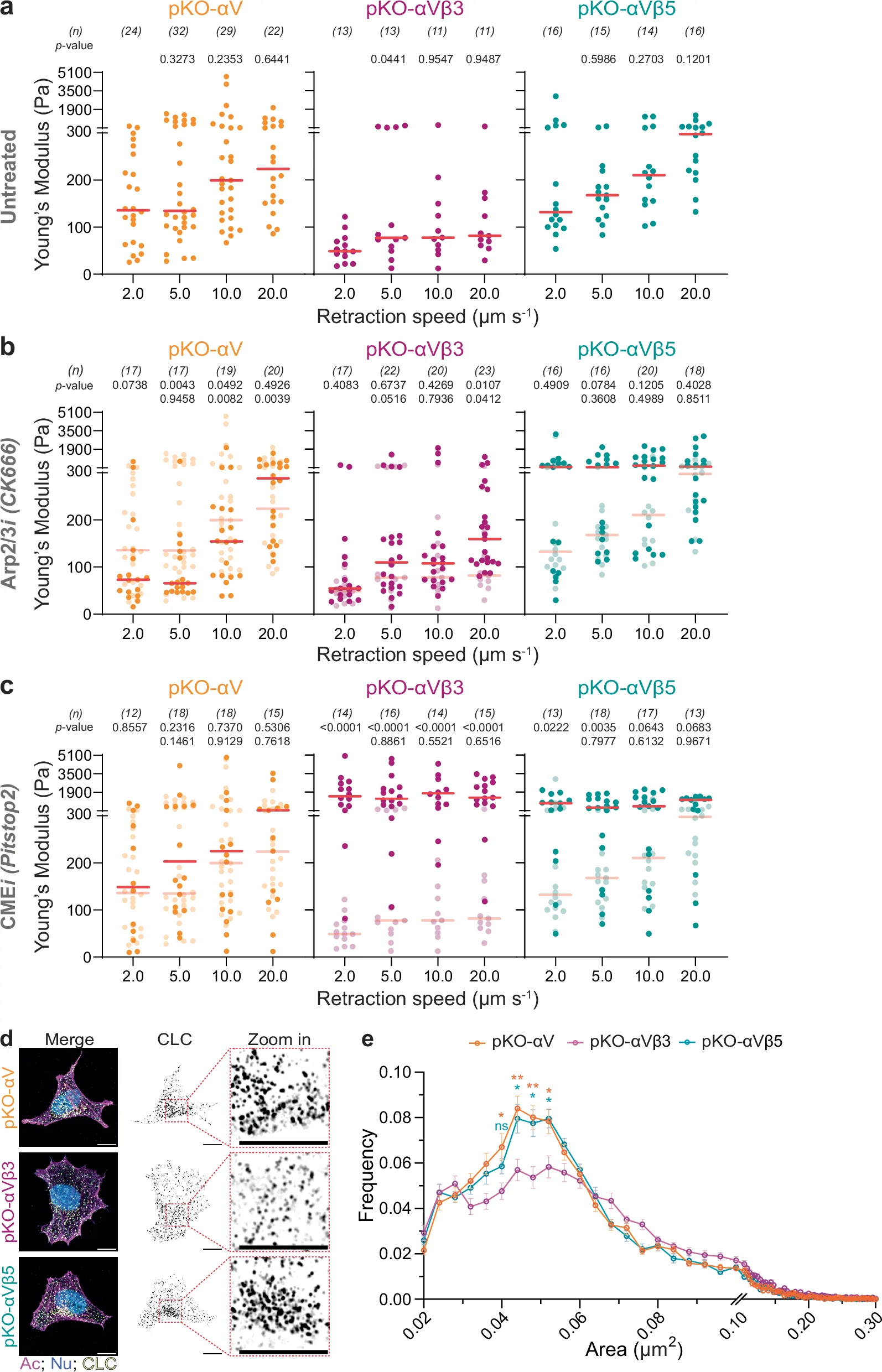
Integrin β-subunit identity, Arp2/3 and clathrin-mediated endocytosis are key determinants of cellular stiffening under mechanical load. **a**–**c** Retraction speed-dependent apparent cell stiffness (Young’s modulus) determined for chemically (un-)perturbed pKO-αV, pKO-αVβ3 and pKO-αVβ5 fibroblasts on vitronectin substrate after 120 s of contact. Fibroblasts were either **a** untreated or were treated with **b** 200 μM Arp2/3 inhibitor CK666, or **c** 30 μM clathrin-mediated endocytosis (CME) inhibitor Pitstop2. Each dot represents one retraction curve obtained by measuring one single cell and correspond to data reported in Fig. 2b, c and Fig. 3a, d; red bars represent median values. *P*-values were obtained using a two-tailed Mann-Whitney U-test. *n* denotes the number of cells measured. *P*-values in the top row compare the data to the unperturbed (lightened) data. *P*-values in the bottom row compare data for a given retraction speed to the preceding speed (e.g., 5 µm s^–1^ with respect to 2 µm s^–1^). **d** Immunofluorescence images with labeled actin (Ac), nucleus (Nu) and clathrin light chain (CLC) in pKO-αV, pKO-αVβ3 and pKO-αVβ5 fibroblasts on vitronectin substrate after 45 min of adhesion. Zoom in, highlighting the CLC assemblies in the respective fibroblasts. Scale bars, 10 μm. **e** Frequency distribution of area quantified for CLC assemblies in pKO-αV (*n* = 16), pKO-αVβ3 (*n* = 16) and pKO-αVβ5 (*n* = 15) fibroblasts. Each data point represents mean ± SE calculated for ≥15 fibroblasts. At area 0.04 µm^2^ the *P*-values ‘cyan’ ns (non-significant) >0.9999 and ‘orange’ * = 0.0121; at area 0.044 µm^2^ the *P*-values ‘cyan’ * = 0.0156 and ‘orange’ ** = 0.0059; at area 0.048 µm^2^ the *P*-values ‘cyan’ * = 0.0180 and ‘orange’ ** = 0.0020; and at area 0.052 µm^2^ the *p*-values ‘cyan’ * = 0.0112 and ‘orange’ * = 0.0414, obtained using Kruskal-Wallis/Dunn’s test. ‘Orange’ *P*-values re*p*resent comparison between pKO-αV and pKO-αVβ3 data. ‘Cyan’ *P*-values re*p*resent comparison between pKO-αVβ5 and pKO-αVβ3 data.

The biphasic cell adhesion response mediated through vitronectin-bound αV-class integrins emerged after 5 s (Fig. 1d), which was more pronounced at 120 s substrate contact, similarly to α5β1 integrins that trigger a biphasic cell adhesion response to fibronectin^48,61^. To further understand the role of αV-class integrins, we applied the Hertz-Sneddon model to extract the effective stiffness (Young’s modulus) of fibroblasts from SCFS data recorded at 120 s contact time to vitronectin (Fig. 4 and Supplementary Fig. 14)^61^. The slope of the retraction force-distance curve from the zero-force inflection point to the minimum force point, just before the cell begins to detach from the substrate, was quantified. By this approach, we obtained an apparent stiffness of a cell having established adhesion for 120 s (Supplementary Fig. 14). Because the adhesion stiffness differs between conditions, the same retraction speed corresponds to different loading rates experienced by the adhesion; the speed-dependent adhesion and rupture forces reported here therefore reflect both bond-level properties and the stiffness-dependent loading rate, which we do not attempt to disentangle. pKO-αV and pKO-αVβ5 fibroblasts showed a tendency towards higher stiffness at higher retraction speeds, which did not reach significance, in contrast to pKO-αVβ3 fibroblasts, which increased stiffness with increasing retraction speed. These responses suggested that the integrin β5-subunit contributes to cellular stiffness regulation. To inquire whether expression of different integrin heterodimers and their respective mechanosensing lead to changes in overall cellular stiffness, we extracted the cellular stiffness from SCFS experiments for pKO-αV, pKO-αVβ3, and pKO-αVβ5 fibroblasts performed in the absence or presence of small molecule inhibitors against Arp2/3, myosin II, F-actin, PI3K, cSrc and CME (Fig. 4 and Supplementary Fig. 14). This also examined if perturbation of the various mechanotransducing signaling pathways impact the apparent cellular stiffness across the different retraction speeds in fibroblasts. Inhibition of F-actin, myosin II, PI3K, and cSrc reduced the stiffness of pKO-αV and pKO-αVβ5 fibroblasts at all retraction speeds, while pKO-αVβ3 fibroblasts increased stiffness mainly at higher retraction speeds for these inhibitions (Supplementary Fig. 14b–e). Similarly, the inhibition of Arp2/3 or CME increased the stiffness of pKO-αVβ5 fibroblasts considerably (Fig. 4b, c). However, CME inhibition also increased the stiffness of pKO-αVβ3 fibroblasts considerably, whereas Arp2/3 inhibition showed no effect. Thus, independent of the integrin β-subunit, CME contributed to regulating the overall fibroblast stiffness. Moreover, the varied effect of Arp2/3 inhibition on the stiffness of pKO-αVβ3 and pKO-αVβ5 fibroblasts suggests that Arp2/3, a known driver of CME^72^, is required for CME only in the case of pKO-αVβ5 fibroblasts. We further characterized the αVβ5 integrin selectivity of CME by quantifying clathrin light chain (CLC) assemblies in pKO-αV, pKO-αVβ3, and pKO-αVβ5 fibroblasts. Only the αVβ5 integrin-expressing pKO-αV and pKO-αVβ5 fibroblasts formed clusters of CLC after 45 min of spreading on vitronectin (Fig. 4d). The area quantification of CLC assemblies showed that pKO-αV and pKO-αVβ5 fibroblasts form similar-sized assemblies as opposed to pKO-αVβ3 fibroblasts (Fig. 4e). Thus, fibroblasts initiating adhesion to vitronectin regulate stiffness through integrin β-subunits for which they employ signaling inputs, including those from CME cascade regulation.

### αVβ3 integrin dominates long-term adhesome composition on vitronectin

**Fig. 5:**
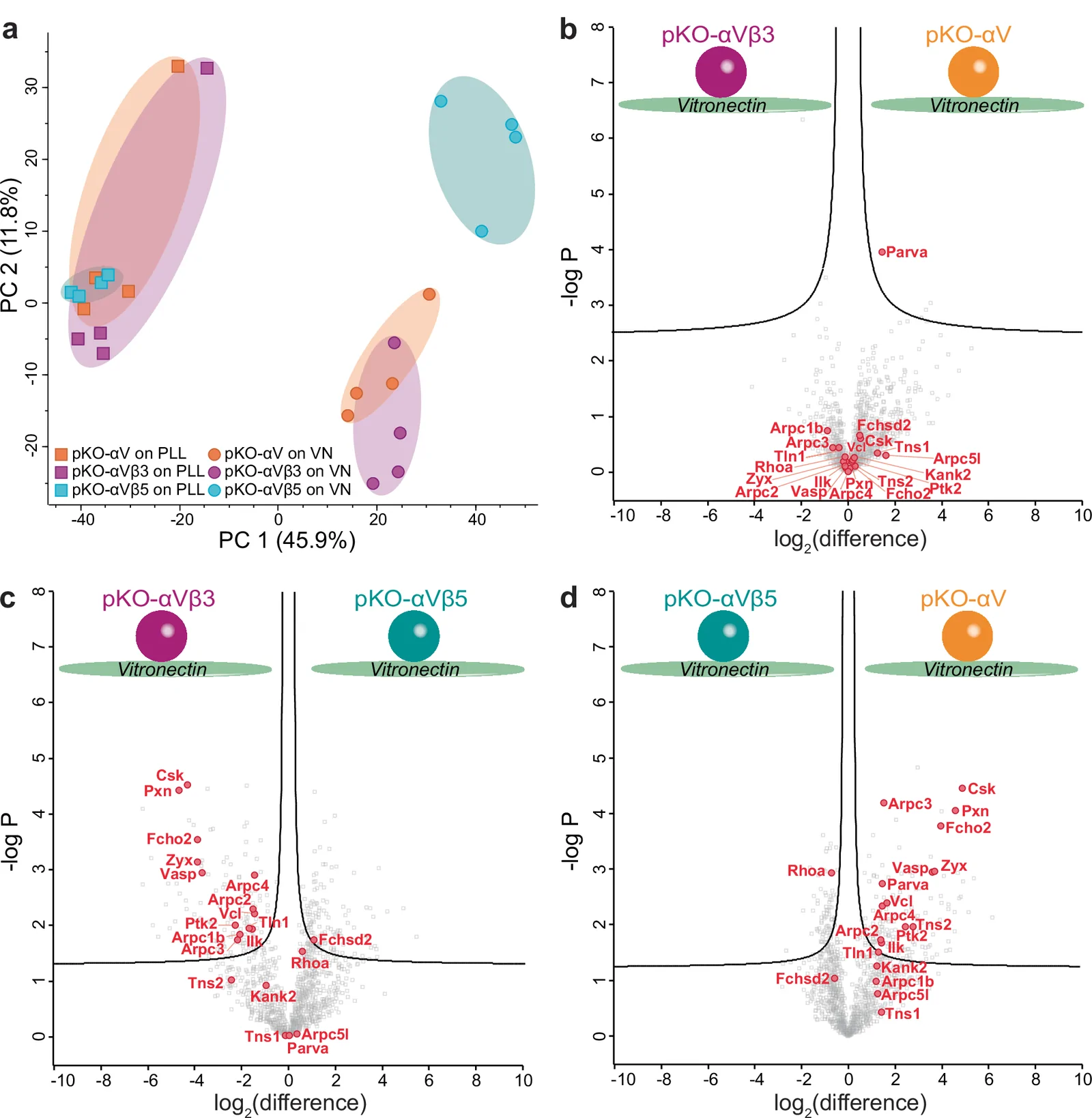
The consensus adhesome of αVβ3 integrin dominates the adhesome composition of pKO-αV fibroblasts on vitronectin. **a** Principal component (PC) analysis for the mass spectrometry of the fibroblast adhesome. Quality check for pKO-αV, pKO-αVβ3 and pKO-αVβ5 fibroblasts cultured on poly-*L*-lysine (PLL) (squares) and vitronectin (circles) used for adhesome mass spectrometry analysis. PC 1 along the x-axis represents 45.9% variation based on the poly-*L*-lysine or vitronectin substrate on which the fibroblasts were cultured. Four replicates for each condition are depicted. **b–d** Consensus adhesome components highlighted to display the comparison of relative protein abundance after 45 min of fibroblast adhesion to vitronectin for **b** pKO-αVβ3 and pKO-αV fibroblasts, **c** pKO-αVβ3 and pKO-αVβ5 fibroblasts, and **d** pKO-αVβ5 and pKO-αV fibroblasts, normalized to poly-L-lysine control. Black lines show the boundaries used to identify protein candidates corresponding to a false-discovery rate of 0.05 and a penalty factor for sample-wide standard deviation of 0.1. The data are representative of four independent replicates.

To gain insight into the molecular underpinnings of the integrin β-subunit-dependent biphasic adhesion response of fibroblast adhesion, we quantitatively and qualitatively analyzed the adhesome composition. To this end, we cultured pKO-αV, pKO-αVβ3 and pKO-αVβ5 fibroblasts for 45 min on vitronectin- or poly-*L*-lysine-coated substrates, crosslinked and isolated the adhesome, released the crosslinks, trypsinized the proteins and analyzed them by mass spectrometry^45,73^ (“Methods”). Principal component analysis showed that the proteome of poly-*L*-lysine cultured fibroblasts clustered separately from vitronectin-cultured fibroblasts (Fig. 5a). Key components of the consensus adhesome like the adapter proteins talin 1 (Tln1), kindlin 2 (Fermt2), vinculin (Vcl), zyxin (Zyx), integrin-linked kinase (Ilk), parvin A (Parva) and paxillin (Pxn) as well as the signaling proteins C-terminal Src kinase (Csk), and focal adhesion kinase (Ptk2) exhibited no marked difference between pKO-αV and pKO-αVβ3 fibroblasts (Fig. 5b and Supplementary Fig. 15a, b). However, the consensus adhesome proteins associated preferentially with αVβ3 integrin (Fig. 5b, c), which indicates that αVβ3 integrin dominates the consensus adhesome assembly when both αVβ3 and αVβ5 integrins are present (Fig. 5b, d). Yet, we did not find adhesome components that have previously been associated with αVβ5 integrins in the context of long-term adhesion of human cancer cells^10^ or keratinocytes^31^, which is most likely due to the much longer adhesion time-frame and/or the different tissue origins. In summary, the data demonstrate a clear divergence between the adhesomes established through integrin β3- and β5-subunits of vitronectin-bound αV-class integrins and confirm that the β3-subunit takes a dominant role in assembling the long-term (45 min) adhesome in pKO-αV fibroblasts expressing both integrin β-subunits.

## Discussion

Integrin-mediated mechanotransduction for the RGD-motif binding αV-class integrins, including αVβ1^54^, αVβ3^24,26^, αVβ5^28–31^, αVβ6^25^, and αVβ8^17,74^, has been studied intensively in recent years. For instance, αVβ3 integrins give rise to focal adhesions on fibronectin, while αVβ5 integrins form focal and reticular adhesions, or flat clathrin lattices on vitronectin^30–32,75^. However, the roles that individual β-subunits of αV-class integrins play during initial substrate contextualization and the establishment of adhesion complexes remain unexplored. Deciphering the molecular underpinnings of such integrin-mediated mechanotransduction is key to a better understanding of cellular homeostasis and disease. Here, we show that fibroblasts employ αV-class integrins to spread faster on vitronectin than on fibronectin, thereby occupying almost twice the area on vitronectin within 5 min (Fig. 1). Upon adhering for longer periods of 45 min to fibronectin or vitronectin, our quantitative mass spectrometric analysis shows that fibroblasts enrich different complement system proteins and ECM proteins (Supplementary Fig. 1). The findings, which align with previous reports indicating that fibronectin serves as a scaffold for collagen^76^, while vitronectin sequesters complement system proteins^77,78^, highlight that fibroblasts use αV-class integrins to distinguish the RGD-context between fibronectin and vitronectin in response to which they employ specific cellular responses to extracellular niches within tens of minutes.

Cells expressing αV-class integrins encounter mechanical cues ranging from intravascular pressure in the cardiovascular system to shear and strain in interstitial tissue^50,79^. We show that within seconds of attaching to vitronectin, fibroblasts employ αV-class integrins to strengthen adhesion biphasically in response to mechanical load (Fig. 1d). Fibroblasts show αV-class integrin-mediated biphasic adhesion strengthening when adhering to the RGD-motif of vitronectin, but not when adhering to other RGD-motif-containing ECM proteins, like fibronectin and osteopontin, or to cyclic RGD peptides (Fig. 1d and Supplementary Fig. 4). Two integrin β-subunits of αV-class integrins exhibit the biphasic adhesion response differently at high (β5-subunit) or low (β3-subunit) adhesion force (Fig. 2b, c, h, i). We have reported a similar phenomenon for the α5β1 integrin-fibronectin bond supported by αVβ3 integrin, which also binds fibronectin^48^. Here, we add the vitronectin-binding of αVβ3 and αVβ5 integrins to the existing literature on the few integrins for which early adhesion and mechanosensation have been quantitatively described^48,61^. Our findings reveal that αVβ3 integrin and αVβ5 integrin employ separate signaling pathways that together adjust cell adhesion and mechanics. The involvement of both pathways in the regulation of cell adhesion and mechanics depends on mechanical load. Ultimately, at a certain mechanical load, one pathway can dominate the other. This discrimination of ECM proteins in conjunction with mechanical sensing suggests that αV-class integrins equip fibroblasts for physiological resilience.

Upon establishing vitronectin as a mechanically important extracellular ligand for fibroblasts, we observe that within fibroblasts, single vitronectin bonds established through αVβ3 integrin dominate the vitronectin bonds formed through αVβ5 integrin. This dominance of αVβ3 integrin, which may originate from its higher intracellular engagement with talin and kindlin^67^ compared to αVβ5 integrin, leads to a higher likelihood of inside-out activation/force transduction of αVβ3 integrin at the cell surface. Thus, despite having a higher affinity for binding to vitronectin, αVβ5 integrin is outcompeted by αVβ3 integrin, which is more likely to be available in an active conformation to bind vitronectin^67^, during the early phases of adhesion. Furthermore, the similarity between αV-class integrin–-vitronectin bond behavior and the biphasic cell adhesion response, together with the distinct signaling molecules strengthening each cell adhesion phase, indicates that the mechanical single integrin bond behavior also involves specific intracellular signaling pathways, with αVβ5 and αVβ3 integrins cooperating to regulate overall cell adhesion. We emphasize that the retraction speed is the experimentally controlled variable, and that differences in adhesion stiffness across conditions translate a given retraction speed into different loading rates. Hence, the measured detachment and rupture forces reflect both bond-level properties and the stiffness-dependent loading rate, which we here do not interpret in terms of single-bond kinetics.

The biphasic cell adhesion response facilitated by αV-class integrins requires force generation through actomyosin regulatory components like myosin II and ROCK (Supplementary Fig. 12a–c). Notably, Arp2/3 inhibition reduces the β-subunit-dependent biphasic adhesion response to different degrees, suggesting that the Arp2/3 complex is differentially recruited to the integrin β3- and β5-subunits and regulates the adhesome rapidly (Fig. 3a). This additional role of Arp2/3 may arise from its involvement as a scaffold within the integrin adhesome, beyond its conventional function as an actin brancher^80^. The actin nucleator mDia and FAK are crucial for the biphasic adhesion response observed for both the integrin β3- and β5-subunits (Supplementary Fig. 12d–f), which extends previous studies in disease models^81,82^ that showed the importance of active FAK for αVβ3 or αVβ5 integrin-mediated angiogenesis and cancer progression, respectively. Although cSrc is reported to contribute to adhesion via β3- and β5-subunits in epithelial and cancer tissues^83–85^, our results provide evidence that during early mechanosensing in fibroblasts, only the integrin β3-subunit requires active cSrc (Fig. 3b). In addition to the actomyosin regulation and adhesion related signaling, we discovered that CME contributes to the αVβ5 integrin-specific biphasic adhesion response of fibroblasts adhering to vitronectin within 120 s. We speculate that it is this early mechanosensory coupling and αVβ5 integrin-specific recruitment of clathrin that results in clathrin-linked adhesion complexes like clathrin-coated pits or flat clathrin lattices after minutes to hours of αVβ5 integrin-mediated adhesion to vitronectin (Fig. 4d)^29,32^. Thus, although the adhesome components, namely Arp2/3 (Fig. 3a), PI3K, cSrc and processes like CME contribute differentially to the integrin β-subunit-dependent biphasic adhesion response (Fig. 3b–d), we find components like F-actin, myosin II, ROCK (Supplementary Fig. 12a–c), mDia (Supplementary Fig. 12d) and FAK (Supplementary Fig. 12e, f) playing conserved roles for both β3- and β5-subunits. Additionally, the early involvement of adapter proteins talin and paxillin in facilitating the biphasic adhesion response adds to the repertoire of proteins that are involved in vitronectin mechanosensing enabled by αV-class integrins.

Whether these regulatory proteins are also found in the integrin β-subunit-associated adhesome at later adhesion times (>>120 s) remained unclear. Using mass spectrometry, we show that the integrin β3- and β5-subunits indeed assemble distinct adhesomes in fibroblasts adhering for 45 min to vitronectin (Fig. 5). Additionally, pKO-αV fibroblasts containing both integrin β3- and β5-subunits show adhesome compositions similar to pKO-αVβ3 fibroblasts containing only the β3-subunit, unlike pKO-αVβ5 fibroblasts containing only the β5-subunit (Supplementary Fig. 15). Moreover, our SCFS data suggest that the stiffness of fibroblasts initiating adhesion to vitronectin is regulated through the integrin β-subunit (Fig. 4 and Supplementary Fig. 14). Specifically, upon establishing cell adhesion, αVβ5 integrin dominates over αVβ3 integrin to regulate the stiffness of fibroblasts with the assistance of the integrin-associated signaling pathways and actomyosin regulatory pathways.

**Fig. 6:**
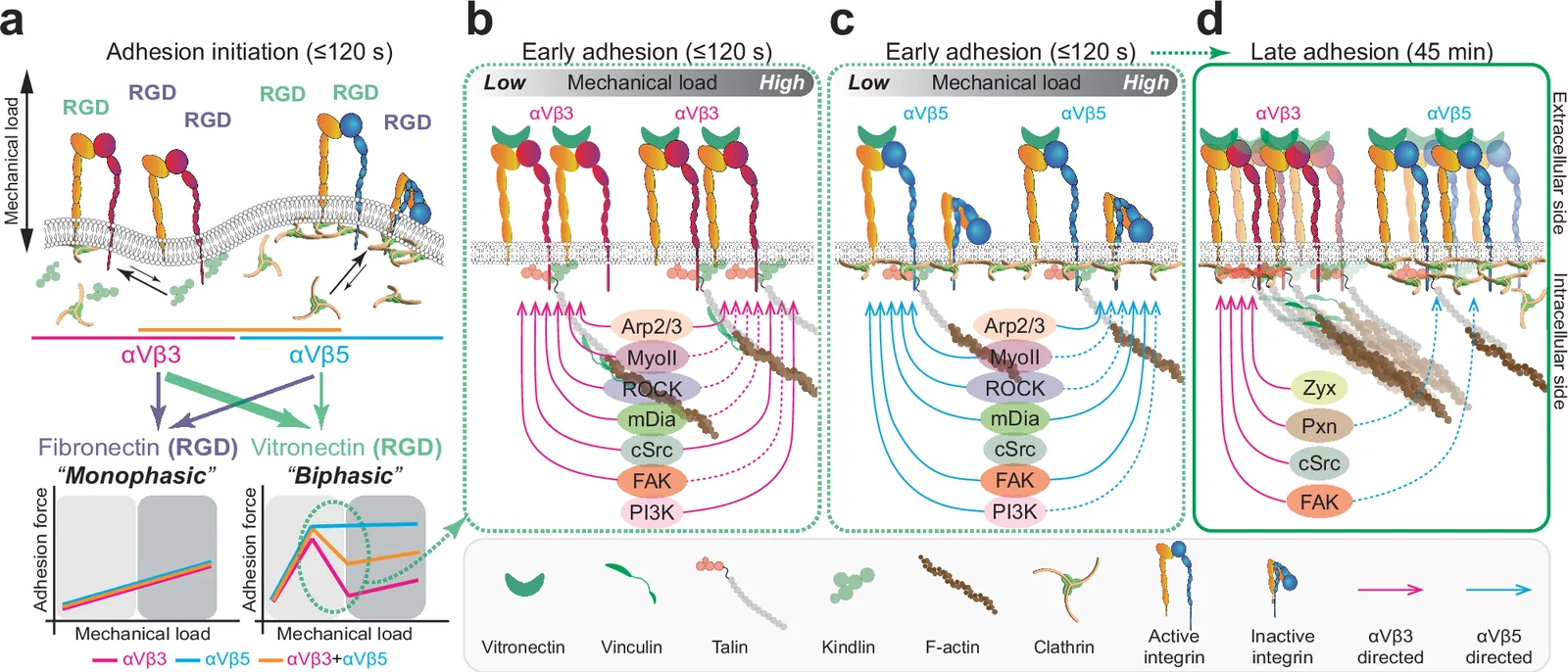
β3- and β5-subunits of αV-class integrins contextualize the extracellular RGD-motif and take different roles in establishing cell adhesion and mechanics in response to mechanical load. **a** Depending on which integrin β-subunit complexes with the αV integrin subunit, αVβ3 or αVβ5 integrins are formed, which bind to and contextualize the RGD-motif in fibronectin and vitronectin to initiate cell adhesion differentially. If the integrins bind the RGD-motif in fibronectin, “monophasic” adhesion strengthening occurs in response to mechanical load. However, if bound to the RGD-motif in vitronectin, fibroblasts strengthen adhesion in a “biphasic” manner in response to load. **b**, **c** Fibroblasts initiate adhesion to vitronectin and couple adhesion regulation to mechanical load. **b** At low mechanical load, αVβ3 integrins strengthen cell adhesion to vitronectin by involving intracellular proteins like Myo II, Arp2/3, ROCK, mDia, cSrc and FAK. With increasing mechanical load, αVβ3 integrins require additional proteins like PI3K to strengthen adhesion, while Myo II, mDia, ROCK and FAK are no longer essential. **c** At low mechanical load, αVβ5 integrins strengthen cell adhesion to vitronectin by involving intracellular proteins like Myo II, ROCK, mDia, FAK, clathrin and PI3K. With increasing mechanical load, αVβ5 integrins additionally require proteins like Arp2/3 to strengthen adhesion, while Myo II, ROCK and PI3K are no longer essential. Dotted arrows in **b** and **c** indicate recruitment of the protein or protein complex to a given integrin only at low mechanical load and not at high mechanical load. Together, **b** and **c** indicate αVβ3 integrin to be the main recruiter of signaling pathways like cSrc, PI3K and FAK, while αVβ5 integrin is the main regulator of cell stiffness through clathrin recruitment. **d** The late adhesome composition of fibroblasts adhering to vitronectin is distinct for αVβ3 and αVβ5 integrins, where αVβ3 integrin dominates over αVβ5 integrin by recruiting most consensus adhesome components, including Zyx, Pxn, cSrc, and FAK, in addition to the proteins described in early adhesion regulation (**b**, **c**). Dotted arrows indicate recruitment of the protein or protein complex to integrin only during early adhesion (≤120 s) and its lack of enrichment in late adhesion (45 min).

In summary, our study provides profound mechanistic insights into how mammalian fibroblasts attach to vitronectin, establish and strengthen adhesion biphasically through αV-class integrins (Fig. 6a). This vitronectin-specific biphasic adhesion response of fibroblasts was not observed for RGD-motifs in fibronectin, osteopontin, or cyclic RGD peptides. Upon binding to vitronectin under low mechanical load, the adhesion force contributed by αVβ3 integrins required regulation of actomyosin integrity and contractility (via F-actin, Myo II, ROCK, Arp2/3 and mDia), along with signaling (via FAK and cSrc) to strengthen adhesion. The adhesion force contributed by αVβ5 integrins also required actomyosin integrity and contractility (via F-actin, Myo II, ROCK and mDia) while the clathrin-mediated endocytosis and signaling pathways (via FAK and PI3K) employed by αVβ5 integrins differed from those employed by αVβ3 integrins (Fig. 6b, c). Under higher mechanical load, adhesion forces contributed by αVβ3 integrins required actin integrity and branching (F-actin and Arp2/3) and signaling (via PI3K and cSrc) to strengthen adhesion. αVβ5 integrins under higher mechanical load required actomyosin nucleation and branching (via mDia and Arp2/3, respectively), signaling (via FAK) and clathrin-mediated endocytosis for the biphasic adhesion response. In addition to this, αVβ5 integrins alone contribute to regulating the cellular stiffness by employing Arp2/3, CME and PI3K, which hints towards a unique membrane-linked stiffness regulatory mechanism. At longer timescales of tens of minutes, the early (≤120 s) mechanosensitive assemblies mature into distinct integrin β-subunit-dependent adhesomes, where molecules like Arp2/3, FAK, cSrc, Zyx, Vcl, and Tln1 enrich in a β3-subunit-specific manner. Thus, we demonstrate that αVβ3 and αVβ5 integrins differ in mediating cellular adhesion and mechanotransduction to vitronectin. This is due to the distinct nature of single integrin αVβ3/αVβ5-vitronectin bonds (Supplementary Fig. 9), actomyosin cortical integrity and regulation (Supplementary Fig. 12), differential involvement of signaling pathways, and membrane involvement in cell stiffness regulation (Supplementary Fig. 14), during the initial phases (≤120 s) of adhesion. Together, these result in early differences in mechanosensing programs that become pronounced and consolidated over tens of minutes and enable the establishment of markedly different adhesomes for the two integrins.

## Methods

### Cell culture

All mouse embryonic fibroblast cell lines were cultured in Dulbecco’s modified Eagle medium (DMEM, 31966047, Thermo Fisher Scientific), supplemented with 10% (v/v) fetal bovine serum (FBS, #F9665, Sigma), 100 U ml^−1^ penicillin and 100 μg ml^−1^ streptomycin (15140122, Thermo Fisher Scientific). Fibroblasts were grown in tissue culture flasks (#TCF012050, Jet BioFil) coated with bovine fibronectin (341631, Calbiochem, Merck Millipore). The fibroblast lines were routinely tested for mycoplasma contamination (MycoTest Kit, #MP0035, Merck) and decontaminated when required (MycoClear Kit, #MP0036, Merck).

### Fibroblast engineering

To deplete β3/β5 integrins in pKO-αV fibroblasts, we used CRISPR/Cas9 as described^86^. Briefly, single-guide RNAs (sgRNAs) targeting two exons were designed using CHOPCHOP^87^ for the *Itgb3* and *Itgb5* genes, using the *Mus musculus* genome (mm10/GRCm38) as a reference. sgRNAs were chosen for high rank, low self-complementarity, low off-target score, and high predicted efficiency. The sgRNAs (with underlined PAM sequences; 5′-NGG-3′) targeted two separate exons [*Itgb3* exon 4 (chr11:104,633,595-104,633,614) 5′-ATGTACATGTACGGCGATACAGG−3′; *Itgb3* exon 10 (chr11:104,643,724-104,643,743) 5′-CCAGCCCACGCTGCAACAATGGG−3′; *Itgb5* exon 3 (chr16:33,865,447-33,865,466) 5′-ACCGTGGATTGCCAAAGTACTGG−3′; *Itgb5* exon 4 (chr16:33,876,034-33,876,053) 5′- ACCCAATACACGGATTGGTCTGG −3′] on the *Itgb3* and *Itgb5* genes. The forward and reverse DNA oligomers encoding for the sgRNAs were phosphorylated by T4 polynucleotide kinase enzyme (#M0201S, New England BioLabs) treatment and then hybridized to form double stranded oligomers. Following a BpiI (#ER1011, Thermo Fisher Scientific)-mediated digestion of backbones containing the Cas9 expression cassette with fluorescent reporter proteins (pSpCas9-2A-BFP and pSpCas9-2A-GFP), the hybridized sgRNA were ligated to the linearized Cas9 backbones using T4 DNA ligase (#M0201S, New England BioLabs). pKO-αV fibroblasts were transfected using Lipofectamine 3000 (#L3000001, Invitrogen) following the supplier's instructions. After 48 h of transfection, we sorted (SONY MA900) the green fluorescence protein (GFP)- and blue fluorescence protein (BFP)-double-positive fibroblasts thereafter cultured them on fibronectin-coated multi-well dishes. Loss of respective integrin expression was verified by immunolabelling and flow cytometric analysis (see “Methods” “*Flow cytometry*” section).

To deplete paxillin (*Pxn*) in pKO-αV and pKO-αVβ5 fibroblasts, CRISPR/Cas9 was employed as described above for integrin depletion. Single-guide RNAs (sgRNAs) for two exons were designed using CHOPCHOP (https://chopchop.cbu.uib.no) for the *Pxn* gene in the reference *Mus musculus* genome (mm10/GRCm38). The sgRNAs (with underlined PAM sequences; 5′-NGG-3′) that we designed targeted two separate exons on the *Pxn* gene: exon 2 (chr5:115,544,490) [5′- CGTGCCATTGAGGGCCTCGCTGG−3′] and exon 8 (chr5:115,552,099) [5′- GTAAGGTCGTGACCGCCATGGGG−3′].

Talin (*Tln*) was depleted in pKO-αV and pKO-αVβ5 fibroblasts using CRISPR/Cas9 as described above. Single-guide RNAs (sgRNAs) for two exons were designed using CHOPCHOP for talin1 (*Tln1*) and talin2 (*Tln2*) genes in the reference *Mus musculus* genome (mm10/GRCm38). The sgRNAs (with underlined PAM sequences; 5′-NGG-3′) targeted two exons of the *Tln1* gene, where exon 2 (chr4:43,556,642) [5′-GGATCCGCTCACGAATCATGCGG−3′] and exon 46 (chr4:43,535,990) [5′-CGAGCTTGACCACATCAGCGAGG−3′] were targeted. For *Tln2* gene, exon 14 (chr9:67,362,656) [5′-ATGGTCGGACAGATGCACCGAGG−3′] and exon 29 (chr9:67,321,836) [5′-AGCTACTTGTAGATTCGGTGAGG−3′] were targeted.

To reintroduce integrins in pKO fibroblasts, we cultured them on fibronectin-coated 6-well plates. At ~70% confluency, we transfected the pKO-αVβ5 fibroblasts with a pCMV3-mItgb3 plasmid (Sino Biological Inc., MG50080-UT) and pKO-αVβ3 fibroblasts with a pCMV-mItgb5 plasmid (Sino Biological Inc., #MG58180-CM) using Lipofectamine 3000. The transfection medium was washed off after ~ 20 h and replaced with culture medium containing 50 µg ml^−1^ hygromycin B (Millipore, 400053) for selection. The hygromycin selection was continued for 3 days with daily replenishment of new antibiotic-containing culture medium. After 3 days of selection, the surviving fibroblasts were stained with anti-mouse CD61 (integrin β3) (1:200, Invitrogen, #12-0611-82) with Armenian hamster IgG control (1:200, Invitrogen, #12-4888-83), anti-αVβ5 (1:200, BD Pharmingen, #565836) with mouse IgGb2(κ) control (1:200, BD Pharmingen, #565378) for flow cytometric analysis (see “Methods”, section “Flow cytometry experiments”). Only cells with positive signals comparable to the parental fibroblasts were sorted (Sony MA900), expanded, and further used for SCFS, cell size, and integrin expression measurements.

### Coating of AFM cantilever and Petri dishes

Tipless 200 µm long V-shaped silicon nitride cantilevers (NP-O: D, Bruker Inc.) were functionalized as described^58^. Briefly, to coat the cantilevers they were plasma cleaned (PDC-32G, Harrick Plasma) for 2–3 min and incubated overnight with 2 mg ml^−1^ concanavalin A (ConA, #C2010, Sigma-Aldrich) at 4 °C. The fibronectin fragment (FNIII7-10) and RGD-deleted fibronectin fragment (FNIII7-10ΔRGD) were expressed and purified as described before^48,88^. For substrate coating, four-segmented polydimethylsiloxane (PDMS, #101697, Dowsil) masks were attached to the glass surface of a Petri dish (FD35-100, WPI). The PDMS-free windows of the masks were incubated with 20 µl of vitronectin, fibronectin, osteopontin, cyclic RGD, or FNIII7-10ΔRGD (all at 50 μg ml^−1^ in PBS) overnight at 4 °C.

### Single-cell force spectroscopy (SCFS)

All fibroblast lines were grown on fibronectin-coated 24-well plates to a maximal confluency of ~80% (#142475, Thermo Fisher Scientific) and serum-deprived in FBS-free DMEM with 100 U ml^−1^ penicillin and 100 μg ml^−1^ streptomycin (#15140122, Thermo Fisher Scientific) overnight before the experiment. SCFS was performed using an AFM (CellHesion 200, JPK Instruments) and a motorized stage (JPK Instruments) mounted on an inverted optical microscope (AxioObserver Z1, Zeiss). The temperature during the measurements was maintained at 37 °C by a temperature controlling system (The Cube, Life Imaging Services). Functionalized tipless, 200 µm long V shaped silicon nitride cantilevers (NP-O: D, Bruker) with a nominal spring constant of 0.06 N m^−1^ were used for SCFS measurements.

To prepare fibroblasts for SCFS, they were detached from the culture flasks using 0.25% (w/v) trypsin (Thermo) for 2 min. Trypsin was inactivated by resuspending the cells in SCFS medium [(DMEM, 12800017, Thermo Fisher) with 20 mM HEPES (A3724, Applichem) and 4 mM sodium bicarbonate, pH 7.2] supplemented with 1% (v/v) FBS, the cells were then pelleted and resuspended in 100 µl SCFS medium. Fibroblasts were then allowed to recover from 0.25% trypsin treatment for 30 min at 37 °C in SCFS medium. In the meantime, ECM protein- functionalized glass-bottom Petri dishes were washed twice with 1X PBS to remove unbound ECM proteins. Next, suspended fibroblasts were added onto FNIII7-10ΔRGD-coated window in the glass-bottom dish. The ConA-coated cantilever was lowered onto a single fibroblast with an approach speed of 10 μm s^−1^ until a contact force of 5 nN was attained. After a contact time of 5 s, the cantilever was retracted at 10 μm s^−1^ until the fibroblast was entirely detached from the substrate. The morphological state of the fibroblasts was continuously monitored throughout the experiment using an optical microscope. Cell adhesion measurements were conducted exclusively on non-blebbing, round, and non-spread fibroblasts of similar diameter. The adhesion force of each fibroblast was quantified by lowering the cantilever onto the substrate-coated glass dish with an approach speed of 5 μm s^−1^. After a contact force of 1 nN was reached, the cantilever height was kept constant for contact times of 5, 20, 50 or 120 s. Afterwards, the cantilever was retracted at 2, 3, 5, 6, 8, 10, 20 or 40 μm s^−1^ for 100 μm until the fibroblast was entirely separated from the substrate (Supplementary Fig. 2a). Adhesion forces were determined after the retraction force-distance curves were drift- and baseline-corrected using the JPK data processing software (version 8.0.92, Bruker Nano GmbH).

### Adhesion probability assay

To perform the adhesion probability assays with single-molecule sensitivity, the AFM (NanoWizard II, JPK Instruments) was mounted on an inverted optical microscope (AxioObserver Z1, Zeiss). The temperature of the experimental setup was maintained at 37 °C by a PetriDishHeater (JPK Instruments). The glass-bottom Petri dish was coated with 50 μg ml^−1^ of vitronectin or FNIII7-10ΔRGD substrate for 2 h at room temperature and washed three times with 1X PBS prior to the measurements. Fibroblasts were attached to the ConA-coated cantilever as described above, before being approached and retracted repeatedly from the substrate-coated Petri dish. The cantilever-attached fibroblast was approached at 1 μm s^−1^ speed to the substrate-coated support until reaching a contact force of 500 pN was reached. The cantilever was retracted immediately afterwards, resulting in a contact time of ~50 ms between fibroblast and substrate. Each fibroblast was subjected to ~100 approach-detachment cycles and within each cycle interacted for ~50 ms with vitronectin. To estimate the binding probability of ligand-receptor bonds, every force-distance curve was analyzed to count the number of binding events using the data processing software of the AFM (version 8.0.92, Bruker Nano GmbH; Supplementary Fig. 2c). The adhesion probability was calculated for each fibroblast as the number of force-distance curves showing single binding events over the total number of curves recorded.

### Analysis of single rupture events from SCFS

To measure the forces rupturing single integrin-vitronectin bonds, rupture events were extracted from force-distance curves using in-house code (Igor 8, Wavemetrics). To analyze single integrin bonds, force-distance curves, acquired as described in the ‘SCFS’ section, were analyzed for pKO-αV, pKO-αVβ3, and pKO-αVβ5 fibroblasts. Rupture events were analyzed following previous reports^58,66^. Briefly, to avoid any contribution of (un-)binding events related to membrane tethers, we analyzed only rupture events up to retraction lengths of 10 µm beyond which the connection between the adhesion assemblies and actomyosin cortex is expected to be lost, and membrane tether events are expected to occur more frequently^58,66^.

### Analysis of apparent cell stiffness

We used JPK data processing software (version 8.0.92, Bruker Nano GmbH) to drift- and baseline-correct the force-distance curves. The curves were smoothened using a boxcar filter with a width of 3.0. The retraction curves were then fitted between the values of zero force and the maximum force before cell detachment to obtain the apparent cell stiffness (Young’s modulus). The fit was performed using the Hertz-Sneddon model for a spherical tip geometry with a radius of 10 μm and a Poisson ratio of 0.5.

### Perturbation assays

Cytoplasmic proteins were perturbed by incubating fibroblasts at 37 °C for 30 min in SCFS media with small molecule inhibitors as follows; 1 µM latrunculin A (LatA, Sigma, #L513), 200 µM CK666 (Tocris Bioscience, #3950), 20 µM blebbistatin (Sigma, #B0560), 10 µM Y27632 (Sigma, #Y0503), 10 µM Y11 (Tocris Bioscience, #6056), 10 µM LY294002 (Cell Signaling Technology, #9901), 20 μM SMIFH2 (MCE, #HY-101445), 20 μM PF573228 (MCE, #HY-10461), or 20 µM PP2 (Tocris Bioscience) in DMSO (to a final composition of 0.1% (v/v) DMSO in SCFS medium). DMSO at 0.1% (v/v) was included as a solvent control and had no effect on adhesion forces from carrier solvents. Fibroblasts were treated with 30 μM Pitstop2 (MCE, #HY-12262) for 10 min. For manganese treatment, the fibroblasts were treated with 0.5 mM MnCl_2_ for 30 min. 10 µg ml^–1^ of the αVβ3 integrin inhibitor P11 (Merck, #407272) was used to pretreat the fibroblasts for 30 min. All chemicals were present in the given concentrations during SCFS.

### Flow cytometry experiments

For flow cytometry analysis, fibroblasts were cultured on fibronectin-coated 24 well plates (Greiner) up to ≈80% confluency. They were serum deprived for >4 h in DMEM (31966047, Thermo Fisher Scientific), supplemented with 100 U ml^−1^ penicillin and 100 μg ml^−1^ streptomycin (15140122, Thermo Fisher Scientific). Serum-deprived fibroblasts were detached from the plates using 0.25% (v/v) trypsin-EDTA (Gibco #25200056) at 37 °C for 2 min. Detached fibroblasts were resuspended in SCFS medium with 1% (v/v) FBS and allowed to recover from detachment at 37 °C for at least 30 min. After recovery, cells were pelleted and 10^5^ fibroblasts were resuspended in 100 µl of flow cytometry buffer (2 mM EDTA and 3% (w/v) BSA in PBS) containing fluorophore conjugated primary antibodies as follows; anti-mouse CD61 (integrin β3) (1:200, Invitrogen, #12-0611-82) with Armenian hamster IgG control (1:200, Invitrogen, #12-4888-83), anti-αVβ5 (1:200, BD Pharmingen, #565836) with mouse IgGb2(κ) control (1:200, BD Pharmingen, #565378). Fibroblasts were stained with the said antibodies for 30 mins at 4 °C in the dark. Afterwards, fibroblasts were washed using flow cytometry buffer twice, followed by measurement of their fluorescence intensity using an LSRFortessa (BD AG). The flow cytometry data were analyzed by using FlowJo (v10, BD AG). Fluorescence-activated cell sorting (FACS) was performed using the culture and staining protocols identical to the flow cytometry analysis experiments. Integrin-negative cells were sorted using a cell sorter (MA900 Sony) at 4 °C in fibronectin-coated 6-well plates. The changes in the cell surface expression of integrins due to perturbation experiments were characterized by flow cytometry using an LSRFortessa Cell Analyzer (BD AG, BD FACSDiva v8.0 software). The cell suspension preparation and immunostaining were conducted as described earlier in this section, whereas the treatment of fibroblasts with small-molecule inhibitors was performed as described above.

### Cell spreading kinetics

To monitor fibroblasts spreading on vitronectin and fibronectin, we used an Airyscan confocal microscope (LSM980, Zeiss), a 20x air objective (Zeiss), and data acquisition software (ZEN Blue version 3.12, Zeiss). pKO-αV fibroblasts were stained with CellTracker™ Green CMFDA (5-chloromethylfluorescein diacetate) dye (excitation/emission spectra of 492/517 nm maxima) (Thermo Fisher Scientific #C7025) to stain the cytoplasm. The fibroblasts were added to fibronectin- and vitronectin-coated Ibidi well plates with inserts and imaged every 30 s using confocal microscopy for > 25 min to analyze their spreading. Laser intensities and gains were kept constant across the experiments. Fluorescence signal was analyzed using Fiji (Version 2.1.0/1.52c). White outline masks used to estimate the projected cell areas were obtained using suitable threshold settings and automated detection of the region of interest around the CellTracker™ signal.

### 3D-Structured illumination microscopy (3D-SIM)

Serum deprived pKO-αV, pKO-αVβ3, and pKO-αVβ5 fibroblasts were plated on glass coverslips coated with 2 mg ml^−1^ concanavalin A (ConA, C2010, Sigma-Aldrich) at a density of 50,000 cells per well for 10 min in SCFS medium. The cells were washed with 1X PBS and fixed using 4% (v/v) paraformaldehyde (in 1X PBS) for 10 min. The cells were blocked in 3% (w/v) bovine serum albumin and 1% (v/v) goat serum (in 1X PBS) for 30 min. pKO-αV and pKO-αVβ3 fibroblasts were immunolabeled with rat anti-mouse integrin β3 (1:200, EmFret) antibody, while pKO-αVβ5 fibroblasts were immunolabeled with rabbit anti-mouse integrin αV (1:200, Abcam) antibody, for 1 h. After washing with 1X PBS, all fibroblasts were immunolabeled with goat anti-rat and goat anti-rabbit AF488 secondary antibodies (1:400, Abcam) for 1 h. After washing with 1X PBS, the coverslips were mounted on a glass slide using 20 µl vectashield anti-fade agent (Vector Technologies). All immunolabeled fibroblasts were imaged using a 3D-SIM microscope (OMX Blaze, API) with a Plan Apo N, 60x/1.42 oil objective with a working distance of 0.15 mm.

### Airyscan confocal microscopy

Knockout of paxillin (PxnKO) in pKO-αV and pKO-αVβ5 fibroblasts was confirmed by immunostaining. Before immunostaining, fibroblasts were allowed to spread on vitronectin for 45 min. The fibroblasts were stained for paxillin using a knockout-verified rabbit anti-mouse monoclonal anti-paxillin antibody (1:200, Abcam). After primary immunostaining, the fibroblasts were stained with DAPI (1:1000) to label nuclei, goat anti-rabbit AF488 (1:400, Abcam) and phalloidin-AF647 (1:400, Invitrogen). The samples were imaged using the SR-4Y Airyscan module of the confocal microscope (LSM980, Zeiss) with a C Apochromat N 40x/1.1 water objective.

### Adhesome proteomics

Quantification of adhesome composition was performed by enrichment and isolation of the adhesion-associated proteome, as described^45,73^. Briefly, for each fibroblast line, four 10 cm diameter Petri dishes per experimental condition were coated with 3 ml of 50 µg ml^−1^ fibronectin, 50 µg ml^−1^ vitronectin, or 0.1% (v/v) poly-*L*-lysine overnight at 4 °C. At 70–90% confluency pKO-αV, pKO-αVβ3, and pKO-αVβ5 fibroblasts were serum-starved for 4 h before being detached with 0.008% (v/v) trypsin-EDTA, obtained by diluting 0.25% (w/v) trypsin-EDTA (Gibco #25200056) in 1X PBS, for 3–5 min, neutralized using 10% (v/v) trypsin inhibitor (in FBS-free DMEM), and allowed to recover from detachment in FBS-free DMEM for 30 min at 37 °C. The fibroblasts were plated on the substrate-coated dishes for 45 min. Afterwards, following a wash with 1X PBS, each dish was incubated on ice with 5 ml PBS (supplemented with 1 mM CaCl_2_ and 1 mM MgCl_2_). Next, samples were treated with 5 ml crosslinker solution (25 mM dithiobis(succinimidyl propionate) (DSP, #22585, Sigma) and 25 mM 1,4-di-[3′-(2′-pyridyldithio)-propionamido]butane (DPDPB, #CL112, Soltec Ventures) in PBS supplemented with 1 mM CaCl_2_ and 1 mM MgCl_2_) for 5 min at room temperature on a rocker. The crosslinking reaction was quenched for 15 min at room temperature by adding 100 mM Tris (pH 7.5) to each dish. Fibroblasts were lysed for 30 min at 4 °C in 3 ml radioimmunoprecipitation assay (RIPA) lysis buffer (25 mM Tris, 150 mM NaCl, 0.1% (w/v) SDS, 0.5% (w/v) sodium deoxycholic acid, one protease inhibitor tablet per 50 ml of RIPA lysis buffer (#04693159001 Roche, Sigma)). Non-covalently bound cellular material was removed by applying high-pressure shear-flow jetting with deionized water for 1 min. 3 ml elution buffer (25 mM Tris, 10 mM NaCl, 0.1% (w/v) SDS, 100 mM dithiothreitol) was added to each dish and incubated for 1 h at 57 °C. Plates were scraped to harvest the eluate. Harvested proteins were precipitated using 12% (v/v) trichloroacetic acid at 4 °C for 30 min. Proteins were pelleted by centrifugation at 5000 x g and 4 °C for 35 min, followed by further centrifugation in ice-cold acetone for 20 min. The pellets were allowed to dry overnight at 4 °C. The protein pellets were then dissolved with buffer containing 6 M urea, 2 M thiourea, 10 mM HEPES (pH 8.0) for in-solution digestion and analyzed by mass spectrometry using an LTQ Orbitrap analyzer (Thermo Electron). Data were analyzed using the label-free quantification (LFQ) algorithm provided by the MaxQuant (version 2.1.0.0) and Perseus software (version 2.0.6.0).

### Statistical analysis

All statistical analysis was performed using GraphPad Prism (software version 10.1.0). Unpaired, nonparametric two-tailed Mann–Whitney U-tests were applied to evaluate statistical significance. *P*-values lower than 0.05 were considered significant.

### Reporting summary

Further information on research design is available in the Nature Portfolio Reporting Summary linked to this article.

## Supplementary information

Supplementary Information

Reporting Summary

Transparent Peer Review file

## Source data

Source Data

## Data Availability

The experimental data generated in this study have been deposited in the ETH research database under accession code http://hdl.handle.net/20.500.11850/803892 and https://doi.org/10.3929/ethz-c-000803892. Any additional requests for information can be directed to, and will be fulfilled by, the corresponding author. Source data are given in the Source Data file of this paper.

## Code availability

The code developed in this study has been deposited in a public repository under https://doi.org/10.3929/ethz-c-000803892.
