# Supplementary material for: Load-dependent RGD-context sensing via αV-class integrins reprograms cell adhesion and mechanics
Source: Nat Commun. 2026 Aug 24;17:10137. doi: 10.1038/s41467-026-77028-8 (PMC13601624; doi:10.1038/s41467-026-77028-8)
Supplement: Supplementary file 1 [file 41467_2026_77028_MOESM1_ESM.pdf]

Supplementary Information

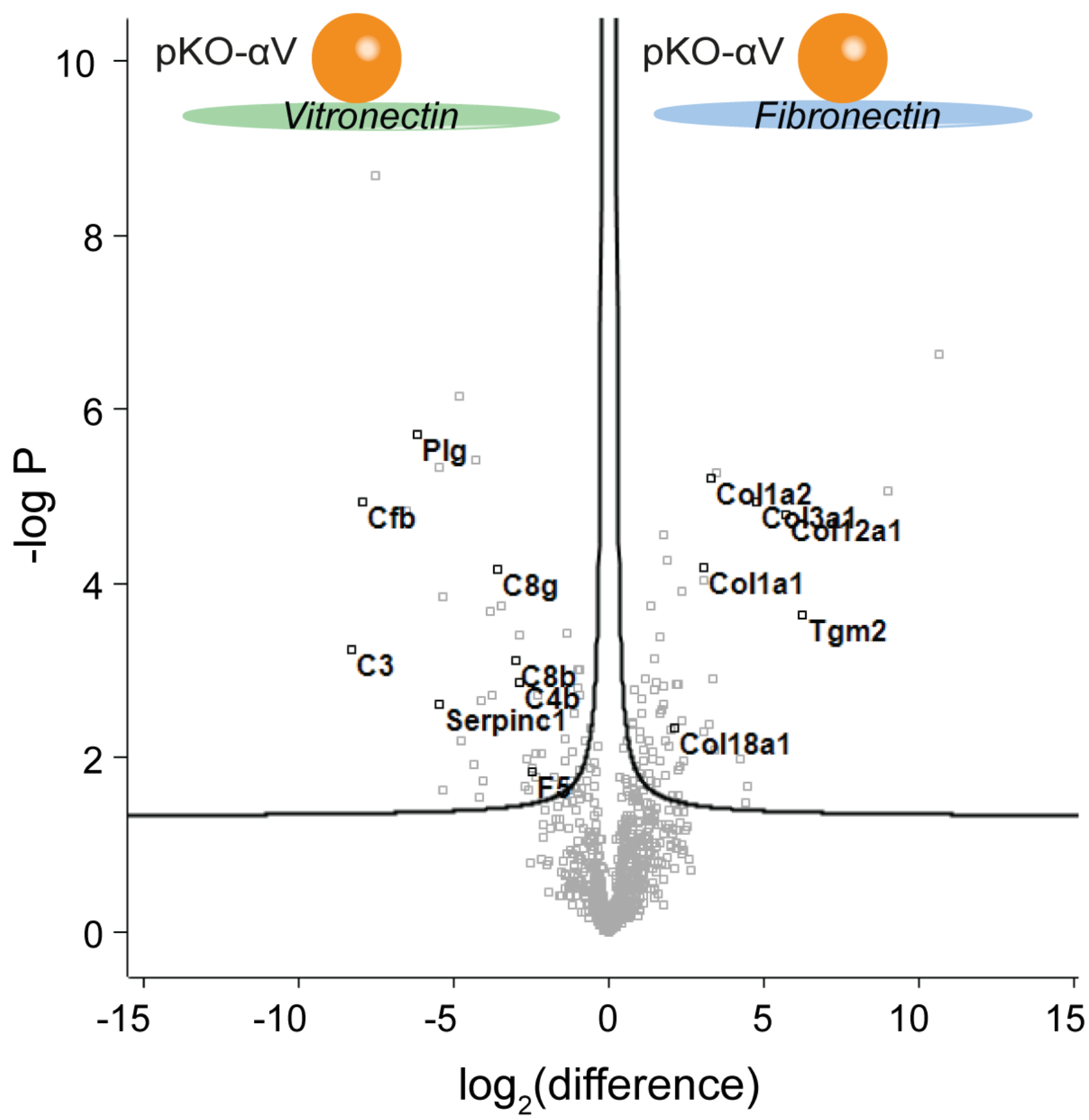

Supplementary Fig. 1. Composition of adhesomes isolated from pKO-αV fibroblasts adhering to fibronectin- or vitronectin-coated glass substrates for 45 min. Volcano plot of mass spectrometry (MS) analysis shows the abundance of ECM-related proteins. Black lines represent the boundaries used to identify protein candidates corresponding to a false-discovery rate of 0.1 and a penalty factor for sample-wide standard deviation of 0.1. Please note that the peptides detected are of murine origin, which is different from the human fibronectin and vitronectin used to plate the cells (Supplementary Table 1). Data are representative of four independent replicates.

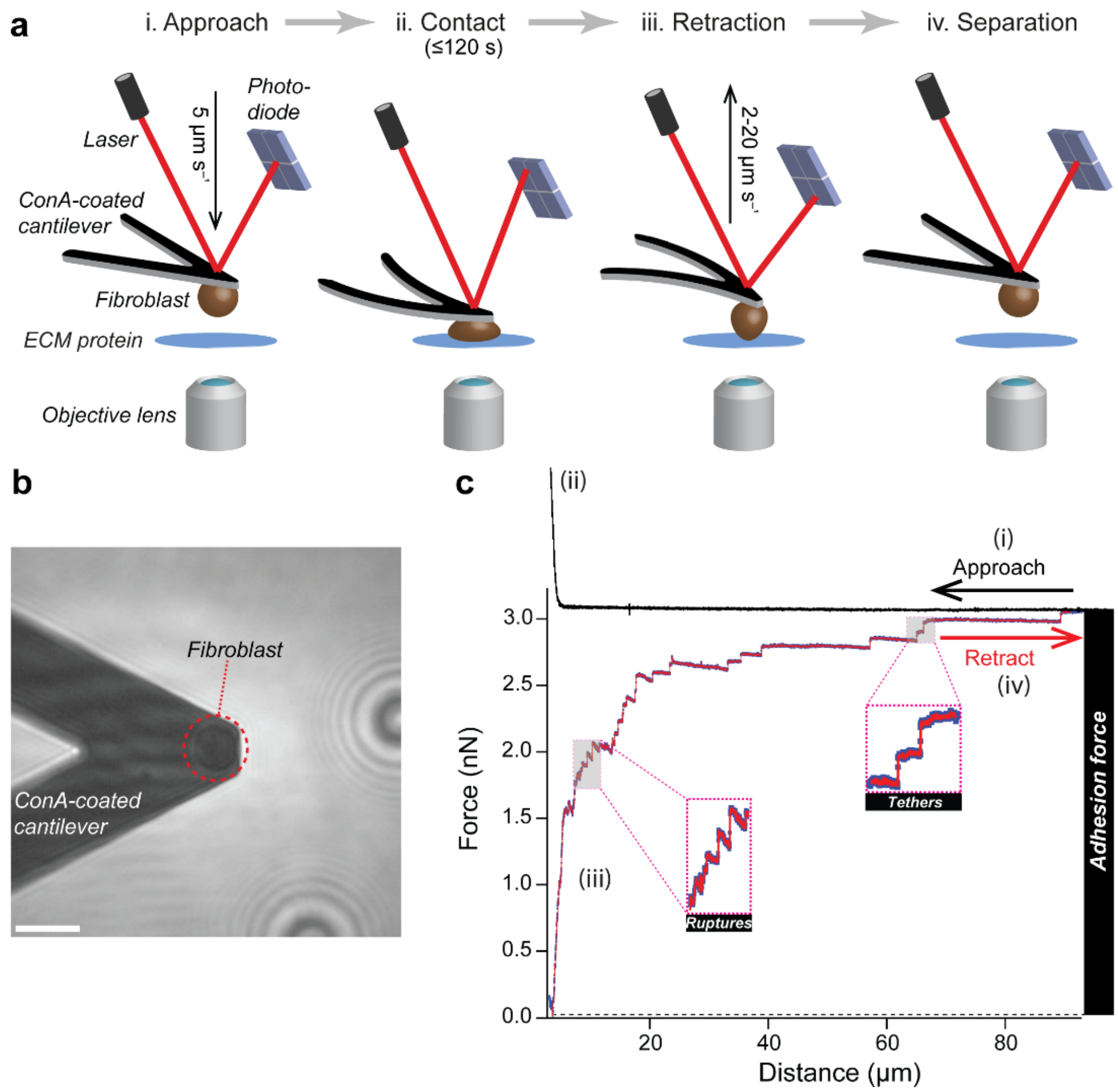

Supplementary Fig. 2. Experimental scheme used to quantify cell adhesion forces to different substrates under variable retraction speeds. a, Schematic of atomic force microscopy (AFM)-based single-cell force spectroscopy (SCFS) depicting a single fibroblast attached to a concanavalin A (ConA)-coated cantilever and approached to a vitronectin- or fibronectin-coated glass surface (substrate) (i) until reaching a contact force of 1 nN (ii). After maintaining contact between fibroblast and substrate for a given contact time (here 5, 20, 50, or 120 s) the cantilever is retracted at different retraction speeds in most cases (here 2-20 µm s^{-1}) to separate fibroblast and substrate while recording a force-distance curve (iii-iv). **b**, Bright field image of a fibroblast on a ConA-coated cantilever. Scale bar, 20 µm. **c**, A force-distance curve tracing the steps (i), (ii), (iii), and (iv) depicted in the SCFS schematic (**a**). The adhesion force of the fibroblast is measured by quantifying the maximum cantilever deflection (force) during retraction. Single membrane 'tether' and integrin-ligand 'rupture' events (inset) occur upon detaching the cell from the substrate[1,2]. Single integrin-ligand 'rupture' events are explored in further detail in Supplementary Fig. 9.

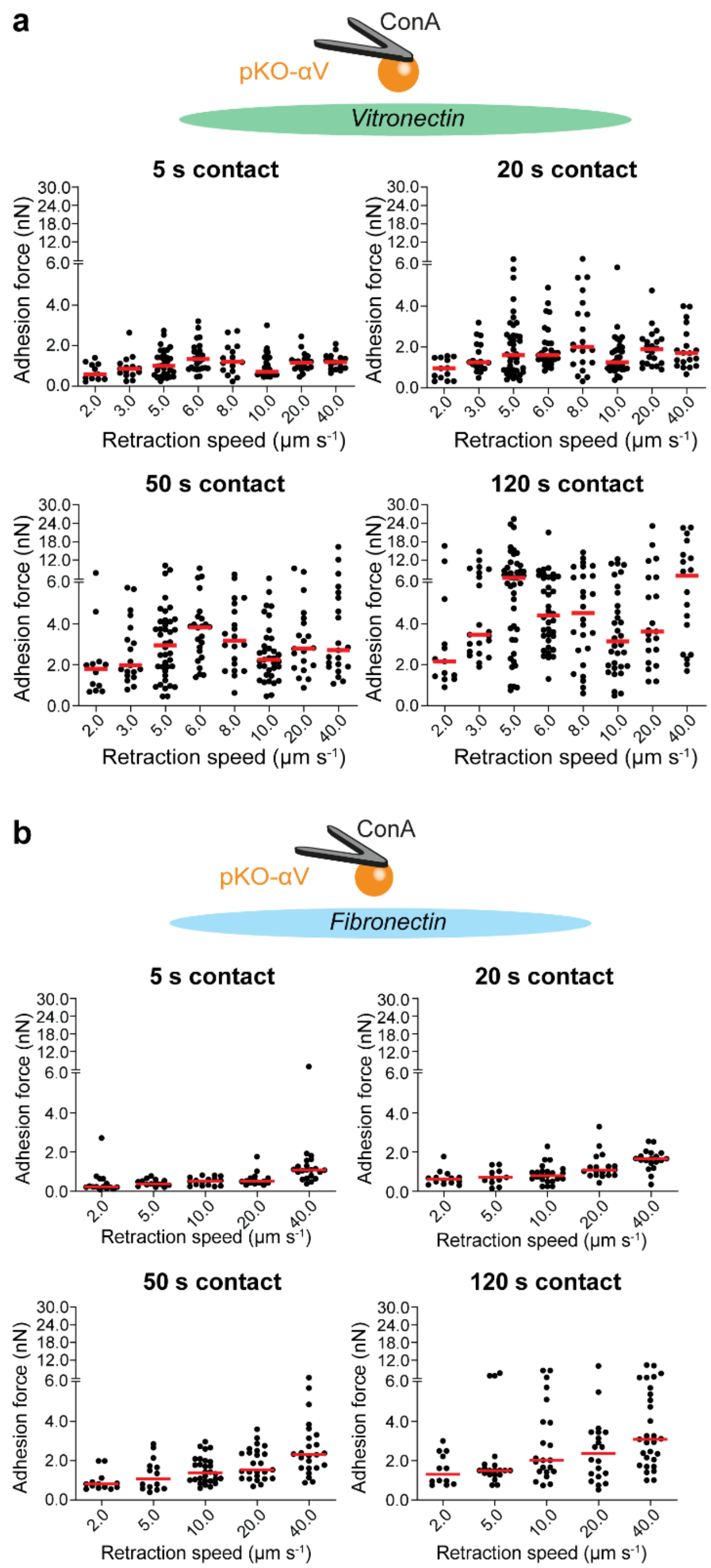

Supplementary Fig. 3. The biphasic adhesion response of fibroblasts is unique to vitronectin and becomes more prominent with increasing contact time. a, Adhesion force of pKO-αV fibroblasts to vitronectin (schematic) increases with retraction speed and contact time. The rapid increase in adhesion force is observed between 2 μm s^{-1} and 5 μm s^{-1} retraction speed and the decrease in adhesion force between 5 μm s^{-1} and 10 μm s^{-1} becomes most noticeable at 120 s contact time. **b**, Adhesion force of pKO-αV fibroblasts to fibronectin (schematic) increases monophasically with retraction speed and contact time. No rapid increase in adhesion force between 2 μm s^{-1} and 5 μm s^{-1} or a reduction in adhesion force between 5 μm s^{-1} and 10 μm s^{-1} is observed. Dots represent the adhesion force of individual fibroblasts and red bars correspond to medians.

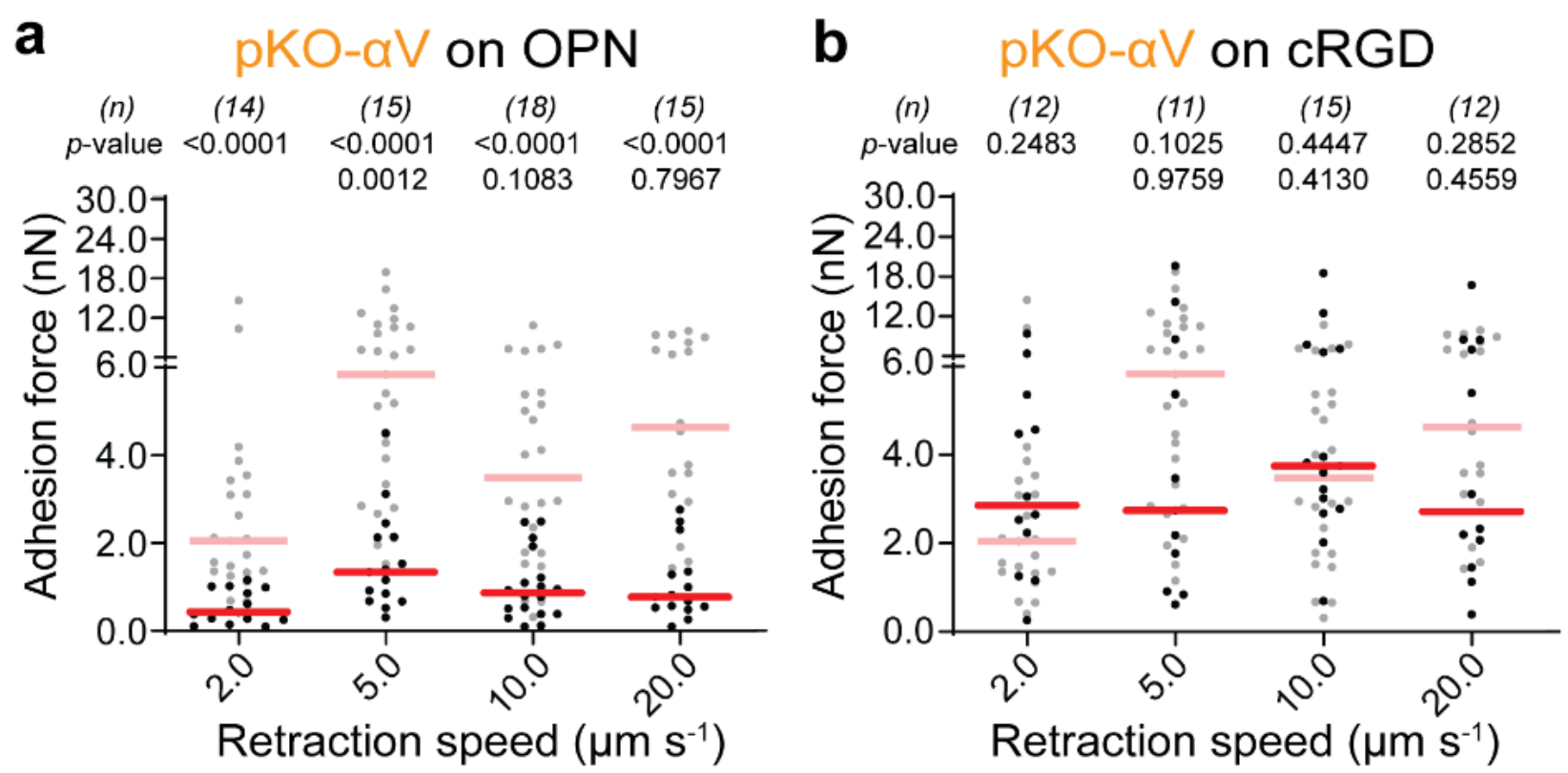

Supplementary Fig. 4. The mechanical-load-dependent adhesion response of pKO-αV fibroblasts to RGD-motif-containing substrates such as osteopontin (OPN) and cyclic RGD (cRGD) [cyclo-RGDfV pentapeptide] is distinct from the biphasic adhesion response to vitronectin. a, The adhesion force of pKO-αV fibroblasts adhering to osteopontin (black) is much lower than when adhering to vitronectin (grey) for 120 s. **b**, The adhesion force of pKO-αV fibroblasts adhering to cRGD (black) is lower than when adhering to vitronectin (grey) for 120 s. Each dot represents the measurement of a single fibroblast; red bars correspond to the median. *p*-values were obtained using two-tailed Mann-Whitney U-test. *p*-values in the top row compare cell adhesion force on osteopontin or cRGD (black) to vitronectin (lightened). *p*-values in the bottom row compare data for a given retraction speed to that of the preceding retraction speed (*e.g.*, 5 μm s^{-1} with 2 μm s^{-1}). *n* denotes the number of fibroblasts characterized on three different experimental days. SCFS measurements were conducted at 120 s contact time at which the mechanical-load-dependent biphasic adhesion response of fibroblasts to vitronectin becomes most noticeable.

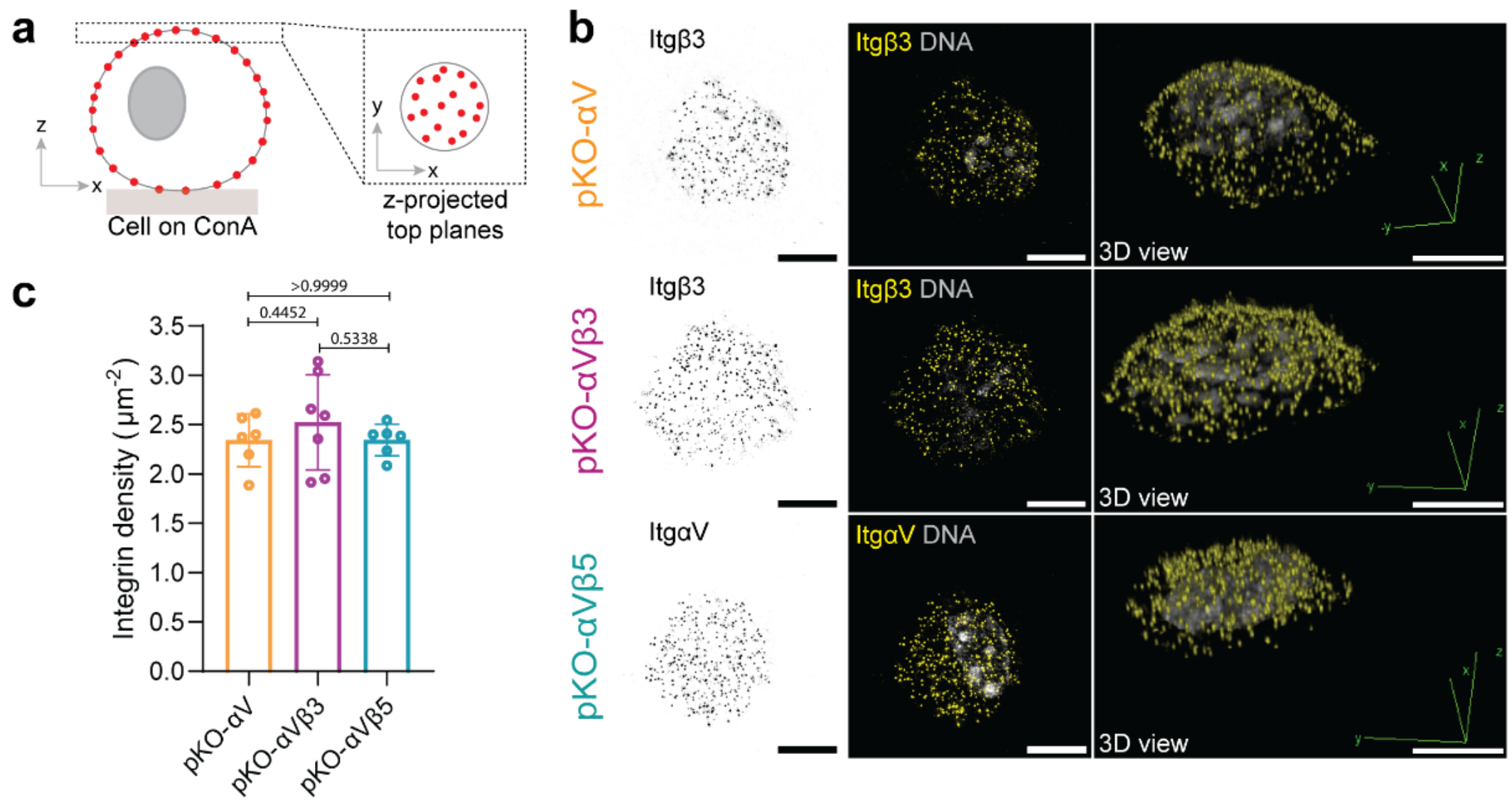

Supplementary Fig. 5. Cell surface density of integrins is similar in pKO-αV, pKO-αVβ3 and pKO-αVβ5 fibroblasts. a, Schematic depicting the strategy used for 3D-structured illumination microscopy (3D-SIM) to observe the integrin (red dots) distribution in fibroblasts plated on a ConA-coated glass coverslip for 5 min. **b**, Left, Inverted SIM images. Middle, composite SIM images visualizing the distribution of integrin β3-subunits (Itgβ3) and αV-class integrins (ItgαV) along the apical surface of pKO-αV, pKO-αVβ3 and pKO-αVβ5 fibroblasts. Right, 3D view (x, y, z axes in green) of the fibroblast apical surface with integrins (yellow) and nucleus (stained for DNA in grey). Scale bars, 5 µm. **c**, Integrin densities for pKO-αV (n = 6), pKO-αVβ3 (n = 7) and pKO-αVβ5 (n = 6) fibroblasts. Bar graphs represent mean ± SD. *p*-values were obtained using two-tailed Mann-Whitney U-test. Each dot represents integrin density from one fibroblast. The experiment was repeated twice revealing similar results.

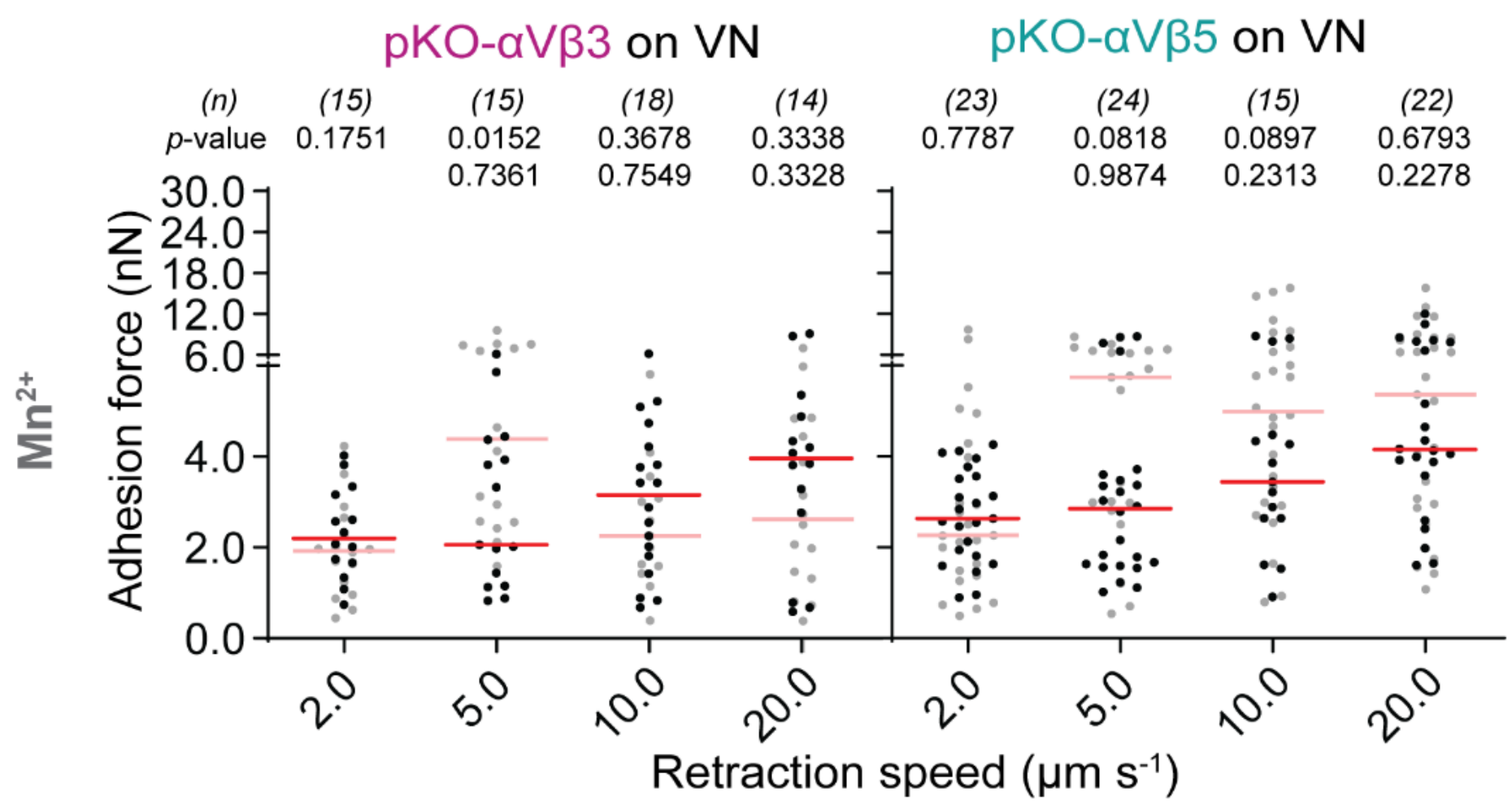

Supplementary Fig. 6. Manganese disrupts the biphasic adhesion response of pKO-αVβ3 and pKO-αVβ5 fibroblasts to mechanical load. Left panel, the adhesion force of Mn^{2+} treated pKO-αVβ3 fibroblasts adhering to vitronectin (black) is lower at 5 µm s^{-1} than for untreated pKO-αVβ3 fibroblasts (grey). Right panel, the adhesion force of Mn^{2+} treated pKO-αVβ5 fibroblasts adhering to vitronectin (black) is lower at 5 µm s^{-1} than for untreated pKO-αVβ5 fibroblasts (grey). Each dot represents the measurement of a single fibroblast, red bars correspond to the median. *p*-values were obtained using two-tailed Mann-Whitney U-test. *p*-values in the top row compare the adhesion force of Mn^{2+} treated (black) to untreated (lightened) fibroblasts. *p*-values in the bottom row compare data for a given retraction speed to that of the preceding retraction speed (*e.g.*, 5 µm s^{-1} with 2 µm s^{-1}). *n* denotes the number of fibroblasts characterized on three different experimental days. SCFS was conducted at 120 s contact time at which the mechanical-load-dependent biphasic adhesion response of fibroblasts to vitronectin becomes most noticeable (Fig. 1 and Supplementary Fig. 3).

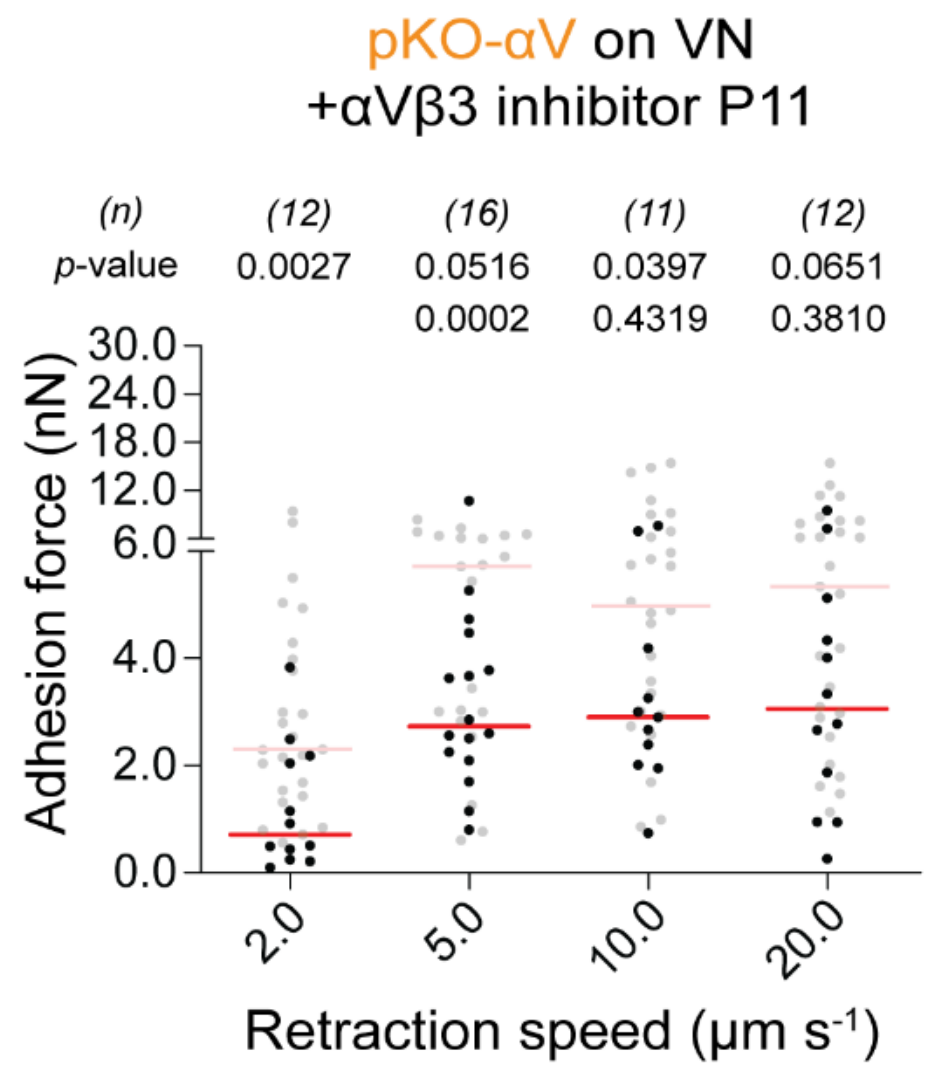

Supplementary Fig. 7. Chemical inhibition of αVβ3 integrin through P11 verifies that the biphasic adhesion response of pKO-αV fibroblasts to vitronectin is mediated through αVβ5 integrin. To verify the αVβ5 integrin-specific biphasic cell adhesion response that plateaus at retraction speeds ≥ 5 µm s^{-1} (Fig. 2b), we chemically inhibited αVβ3 integrin using 10 µg ml^{-1} P11 for 30 min (preincubation). As a consequence of this inhibition, we observed a biphasic adhesion response trend similar to that mediated through αVβ5 integrin alone (Fig. 2c), although at lower cell adhesion force. The data was recorded at 120 s contact time on vitronectin (VN). Dots represent the adhesion force of individual fibroblasts and red bars correspond to medians. *p*-values were obtained using two-tailed Mann-Whitney U-test. *p*-values in the top row compare the data to the pKO-αVβ5 fibroblasts on vitronectin control (lightened) data. *p*-values in the bottom row compare data for a given retraction speed to the preceding retraction speed (*e.g.,* 5 µm s^{-1} with respect to 2 µm s^{-1}). *n* gives the number of fibroblasts characterized.

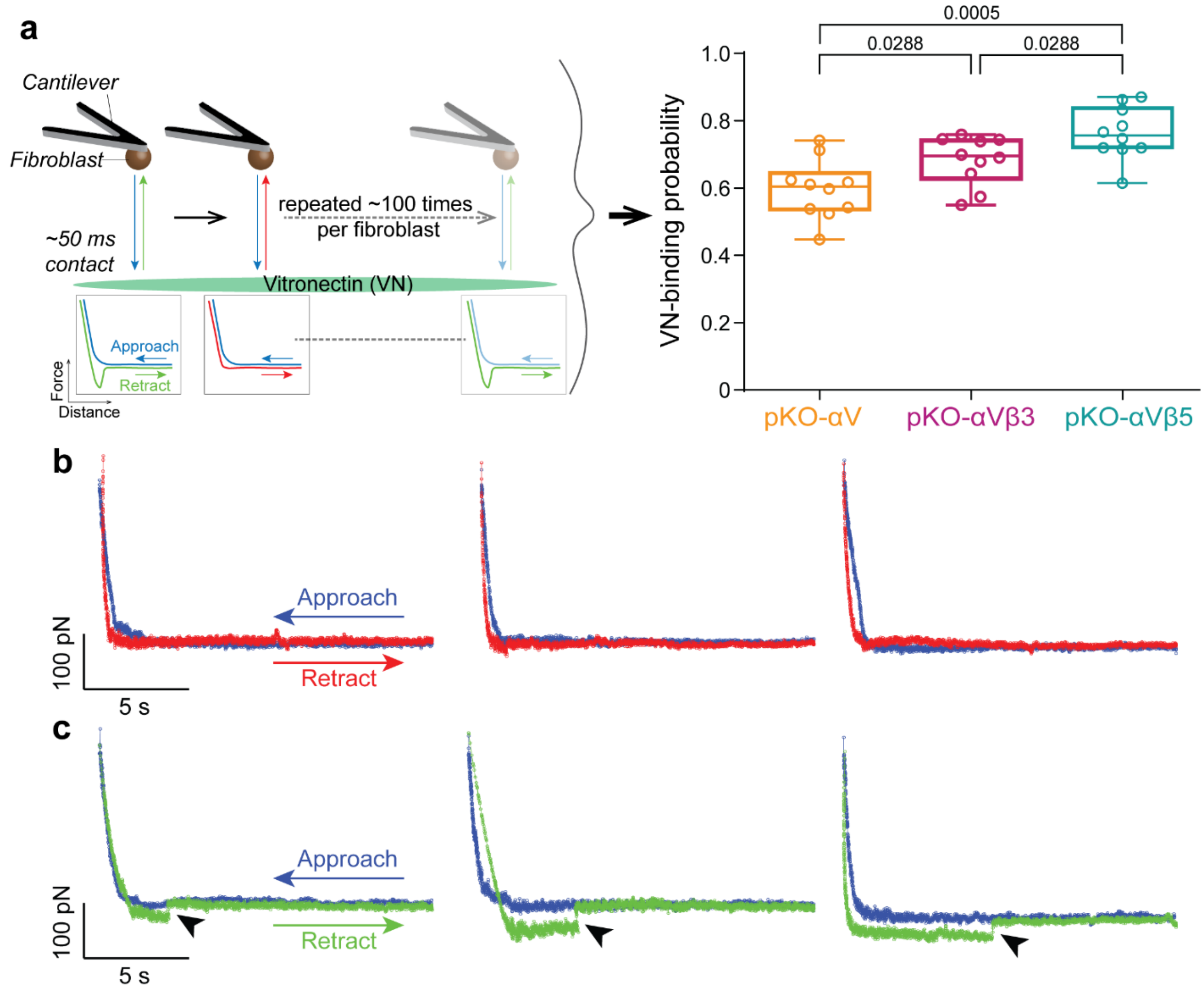

Supplementary Fig. 8. Binding probability of αV-class integrins to vitronectin. a, Vitronectin-binding probability of pKO-αV, pKO-αVβ3 and pKO-αVβ5 fibroblasts as measured by repeatedly approaching and retracting a single fibroblast to a vitronectin (VN)-coated substrate at 1 μm s^{-1} velocity and ~50 ms contact time (Methods). Each data point represents an outcome from ~100 approach-retraction cycles per cell collected for 10 different fibroblasts. Box plots represent median and interquartile range. *p*-values were obtained using Mann-Whitney U-test. **b**, Three example force-time curves when no unbinding event between fibroblast and substrate is recorded. **c**, Three example force-time when an unbinding event (black arrow heads) between fibroblast and substrate is recorded.

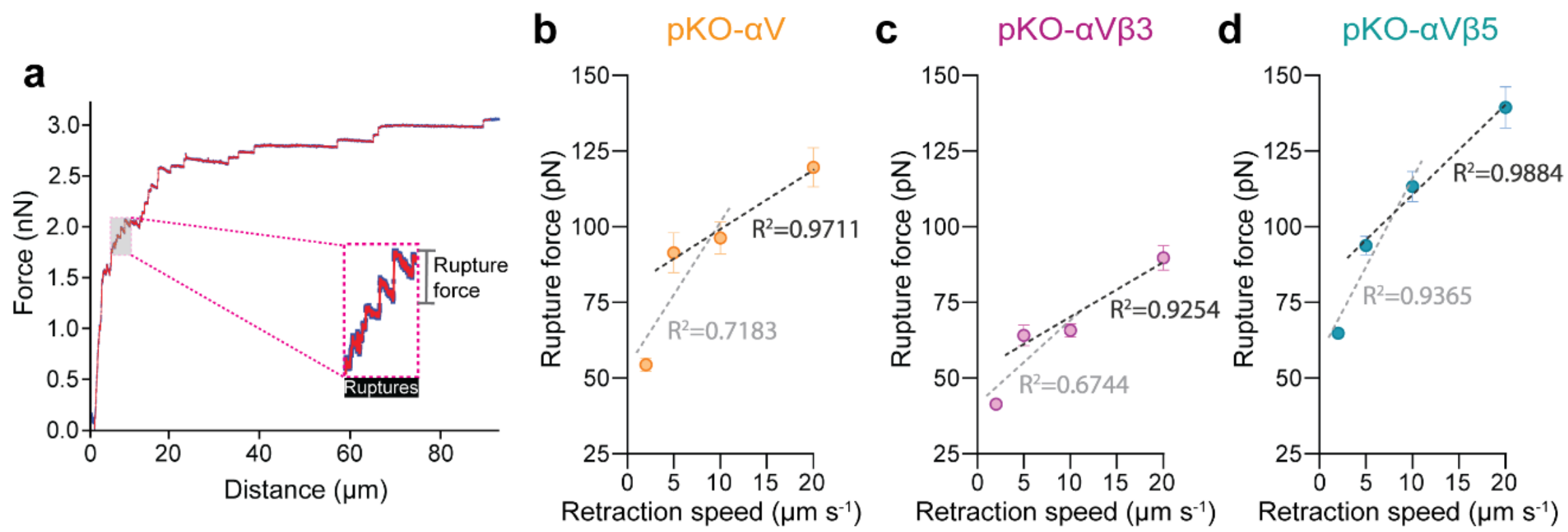

Supplementary Fig. 9. Rupture force of individual integrin-vitronectin bonds depends differently on retraction speed. a, Schematic of analyzing 'rupture' events (inset) of a force-distance curve recorded upon retracting a fibroblast from vitronectin by SCFS. The rupture events highlighted quantify the rupture force of ligand-integrin bonds[1,3]. Single rupture events of integrins adhering to vitronectin were analyzed from single-cell force spectroscopy (SCFS) data recorded for Fig. 1d and Fig. 2b,c. **b**, The rupture force of αV-class integrin-vitronectin bonds depends non-linearly on the retraction speed in pKO-αV fibroblasts. In pKO-αV fibroblasts both, αVβ3 and αVβ5 integrins can bind vitronectin. **c**, The rupture force of αVβ3 integrin-vitronectin bonds depends non-linearly on the retraction speed in pKO-αVβ3 fibroblasts. In pKO-αVβ3 fibroblasts only αVβ3 integrin can bind vitronectin. **d**, The rupture force of αVβ5 integrin-vitronectin bonds depends linearly on the retraction speed in pKO-αVβ5 fibroblasts. In pKO-αVβ5 fibroblasts only αVβ5 integrin can bind vitronectin. Number of single rupture events analyzed in (**b**) for 2 µm s^{-1}=352; 5 µm s^{-1}=313; 10 µm s^{-1}=239; 20 µm s^{-1}=130, in (**c**) for 2 µm s^{-1}=229; 5 µm s^{-1}=183; 10 µm s^{-1}=165; 20 µm s^{-1}=120, and in (**d**) for 2 µm s^{-1}=587; 5 µm s^{-1}=339; 10 µm s^{-1}=225; 20 µm s^{-1}=132. Circles represent mean rupture force and error bars SEM.

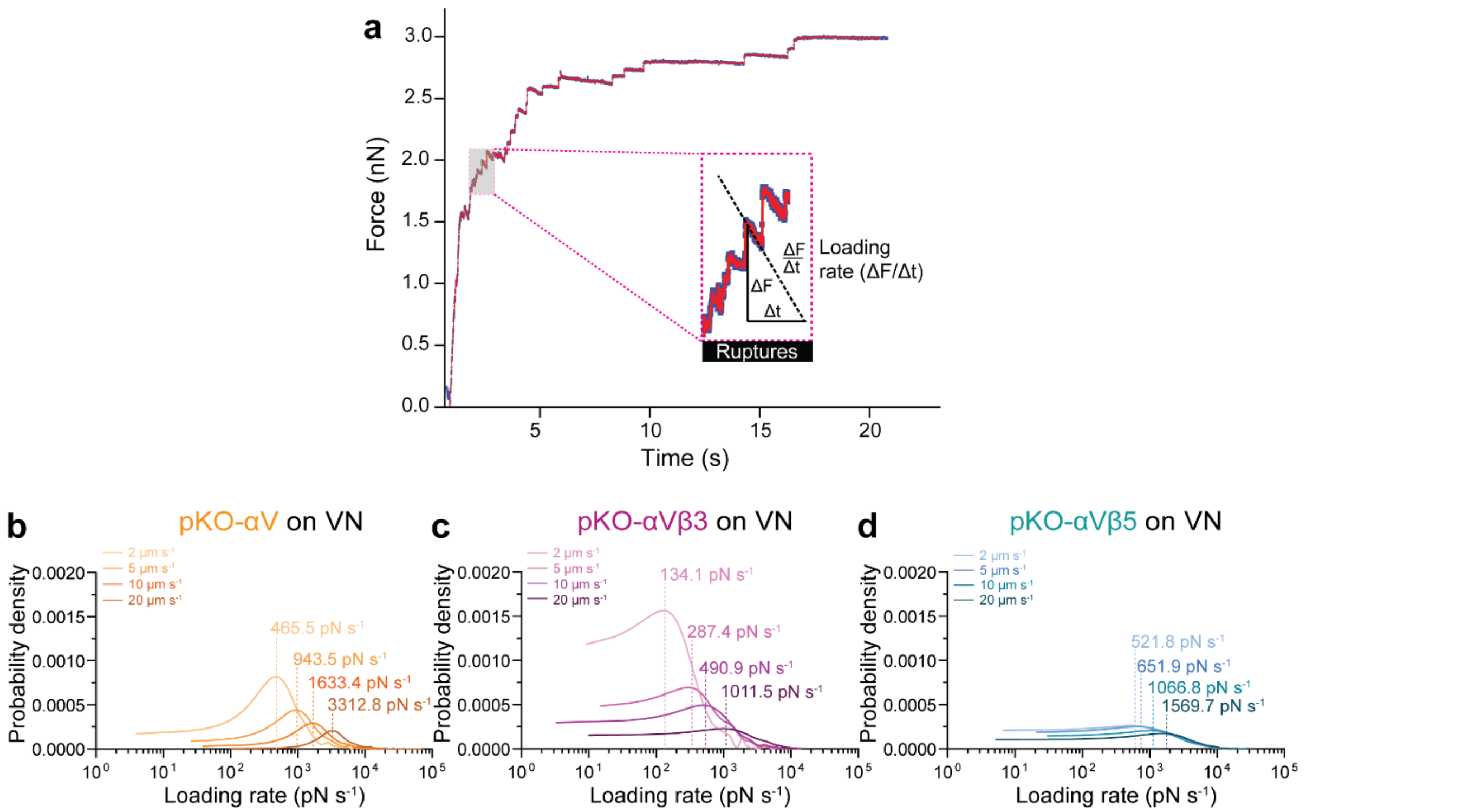

Supplementary Fig. 10. Loading rate experienced by single integrin-vitronectin bonds depends on the retraction speed. a, Schematic of analyzing loading rates ($\Delta F/\Delta t$) (inset) for a ligand-integrin bond detected by rupture events in a force-time curve recorded upon retracting a fibroblast from vitronectin by SCFS. **b-d**, Probability density of loading rates (*i.e.*, force applied over time) to which individual integrin-vitronectin bonds are exposed in (**b**) pKO-αV fibroblasts, (**c**) pKO-αVβ3 fibroblasts and (**d**) pKO-αVβ5 fibroblasts. Values in the graphs give the most expected loading rates at each retraction speed before bond rupture. The experimental data are taken from Fig. 1d and Fig. 2b,c for analysis. Number of single rupture events analyzed to obtain the loading rate in (**b**) for 2 µm s^{-1}=352; 5 µm s^{-1}=313; 10 µm s^{-1}=239; 20 µm s^{-1}=130, in (**c**) for 2 µm s^{-1}=229; 5 µm s^{-1}=183; 10 µm s^{-1}=165; 20 µm s^{-1}=120, and in (**d**) for 2 µm s^{-1}=587; 5 µm s^{-1}=339; 10 µm s^{-1}=225; 20 µm s^{-1}=132.

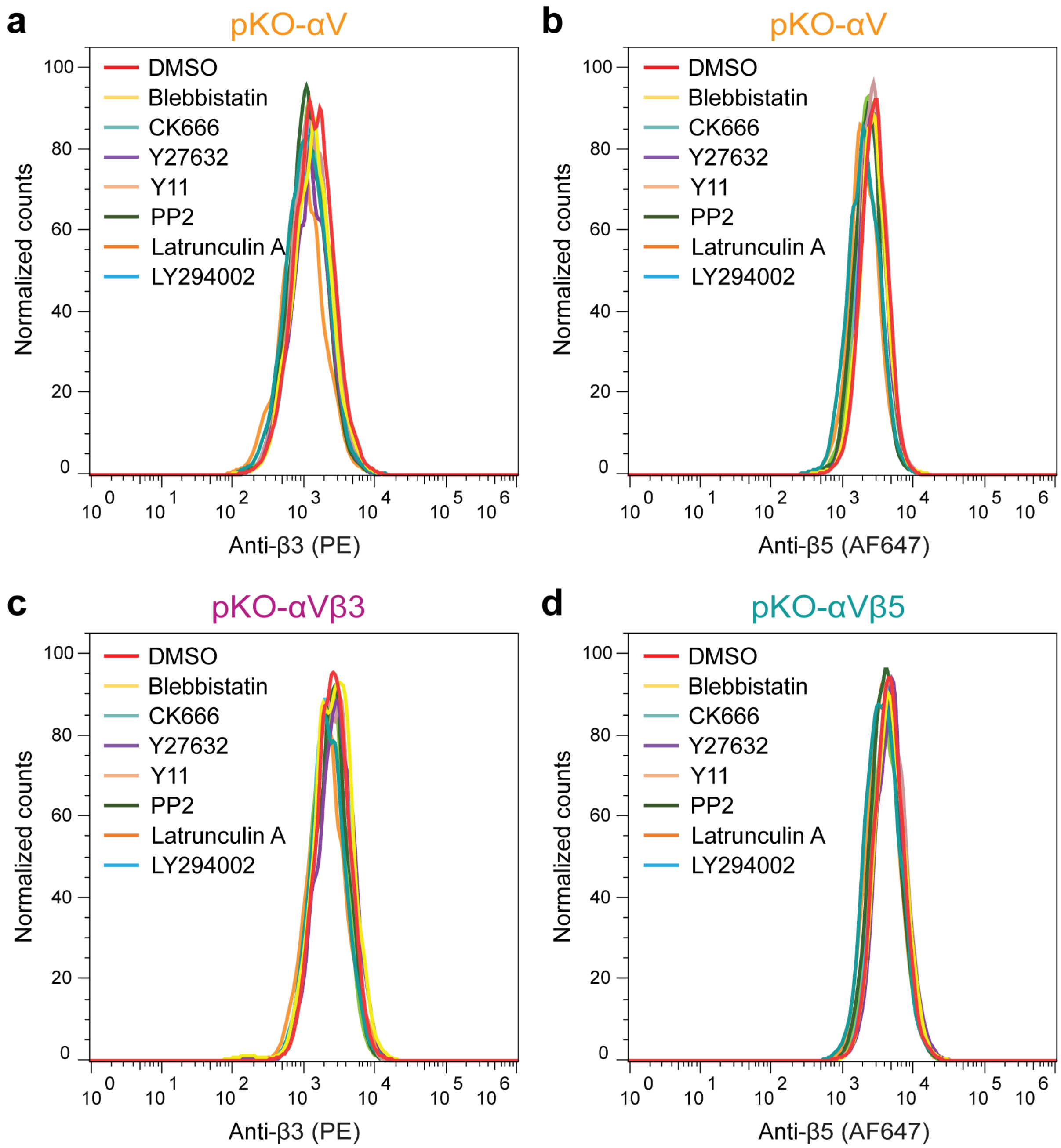

Supplementary Fig. 11. Integrin β-subunit cell surface expression is independent of small molecule inhibitor treatment. To confirm the surface expression of integrin β3 and β5-subunit(s) in (**a**, **b**) pKO-αV fibroblasts, (**c**) pKO-αVβ3 fibroblasts, and (**d**) pKO-αVβ5 fibroblasts using flow cytometry analysis, the fibroblasts were treated with DMSO (0.1% v/v), 20 µM blebbistatin, 200 µM CK666, 10 µM Y27632, 10 µM Y11, 20 µM PP2, 1 µM latrunculin A, or 10 µM LY294002 for 30 min before being doubly stained with anti-β3 subunit and anti-β5 subunit fluorophore-tagged monoclonal antibodies [control for Fig. 3 and Supplementary Fig. 12]. **a**, Surface expression of αVβ3 integrin of pKO-αV fibroblasts remains the same for all treatments. **b**, Surface expression of αVβ5 integrin of pKO-αV fibroblasts remains the same for all treatments. **c**, Surface expression of αVβ3 integrin in pKO-αVβ3 fibroblasts remains the same for all treatments. **d**, Surface expression of αVβ5 integrin in pKO-αVβ5 fibroblasts remains the same for all

treatments. 5000 fibroblasts were analyzed per sample and filtered for live and single cells. The data are representative of two independently repeated experiments.

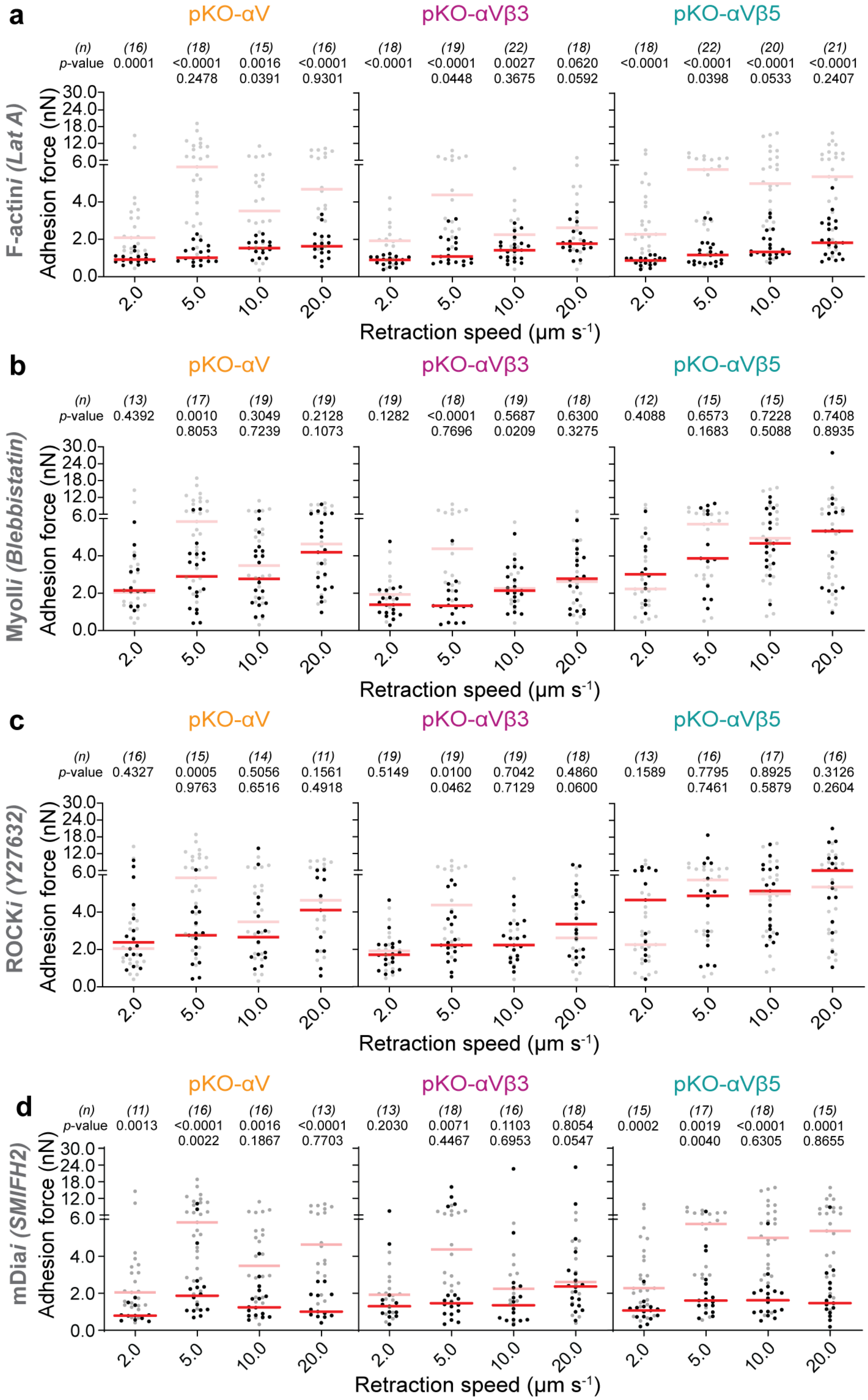
a
pKO-αV
pKO-αVβ3
pKO-αVβ5
(n) (16) (18) (15) (16) (18) (19) (22) (18) (18) (22) (20) (21)
p-value 0.0001 <0.0001 0.0016 <0.0001 <0.0001 <0.0001 0.0027 0.0620 <0.0001 <0.0001 <0.0001 <0.0001
0.2478 0.0391 0.9301 0.0448 0.3675 0.0592 0.0398 0.0533 0.2407
F-actini (Lat A)
Adhesion force (nN)
30.0 24.0 18.0 12.0 6.0 4.0 2.0 0.0
2.0 5.0 10.0 20.0 2.0 5.0 10.0 20.0 2.0 5.0 10.0 20.0
Retraction speed (μm s-1)
b
pKO-αV
pKO-αVβ3
pKO-αVβ5
(n) (13) (17) (19) (19) (19) (18) (19) (18) (12) (15) (15) (15)
p-value 0.4392 0.0010 0.3049 0.2128 0.1282 <0.0001 0.5687 0.6300 0.4088 0.6573 0.7228 0.7408
0.8053 0.7239 0.1073 0.7696 0.0209 0.3275 0.1683 0.5088 0.8935
MyoIIi (Blebbistatin)
Adhesion force (nN)
Retraction speed (μm s-1)
c
pKO-αV
pKO-αVβ3
pKO-αVβ5
(n) (16) (15) (14) (11) (19) (19) (19) (18) (13) (16) (17) (16)
p-value 0.4327 0.0005 0.5056 0.1561 0.5149 0.0100 0.7042 0.4860 0.1589 0.7795 0.8925 0.3126
0.9763 0.6516 0.4918 0.0462 0.7129 0.0600 0.7461 0.5879 0.2604
ROCKi (Y27632)
Adhesion force (nN)
Retraction speed (μm s-1)
d
pKO-αV
pKO-αVβ3
pKO-αVβ5
(n) (11) (16) (16) (13) (13) (18) (16) (18) (15) (17) (18) (15)
p-value 0.0013 <0.0001 0.0016 <0.0001 0.2030 0.0071 0.1103 0.8054 0.0002 0.0019 <0.0001 0.0001
0.0022 0.1867 0.7703 0.4467 0.6953 0.0547 0.0040 0.6305 0.8655
mDiai (SMIFH2)
Adhesion force (nN)
Retraction speed (μm s-1)

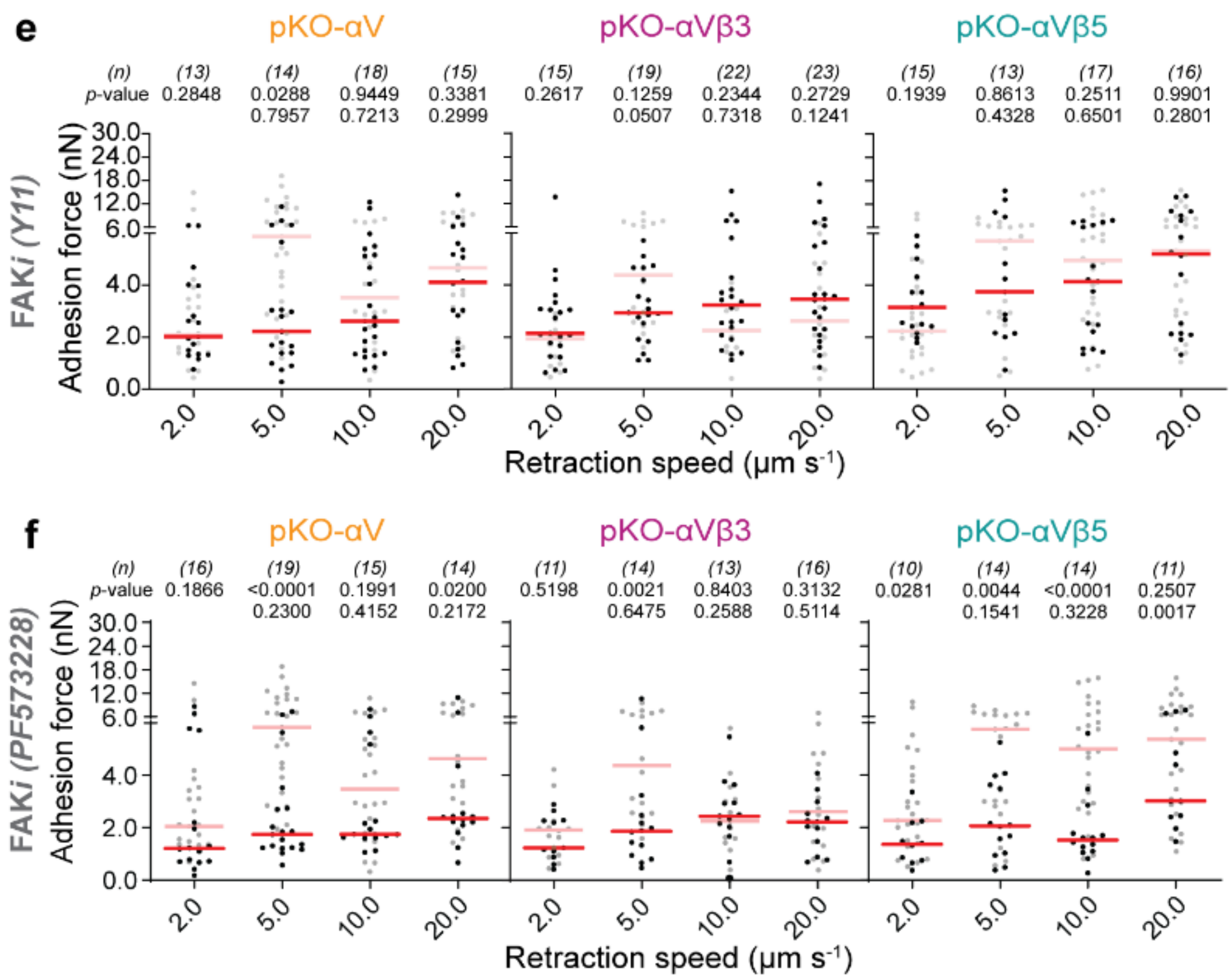

Supplementary Fig. 12. Integrity and regulation of actomyosin cortex together with FAK mediate the biphasic response of vitronectin-bound αV-class integrins. a-f, Retraction speed-dependent adhesion force of chemically perturbed pKO-αV, pKO-αVβ3 and pKO-αVβ5 fibroblasts adhering to vitronectin for 120 s contact time. Fibroblasts were treated with (**a**) 1 µM filamentous actin (F-actin) inhibitor latrunculin A (LatA), (**b**) 20 µM myosin II (Myo II) inhibitor blebbistatin, (**c**) 10 µM Rho kinase (ROCK) inhibitor Y27632, (**d**) 20 µM mDia inhibitor SMIFH2, (**e**) 10 µM focal adhesion kinase (FAK) inhibitor Y11 and (**f**) 20 µM focal adhesion kinase (FAK) inhibitor PF573228. Each dot represents one fibroblast, red bars the median. *p*-values were obtained using two-tailed Mann-Whitney U-test. *p*-values in the top row compare data to unperturbed (lightened) data. *p*-values in the bottom row compare data for a given retraction speed to the preceding speed (*e.g.*, 5 µm s^{-1} with 2 µm s^{-1}). *n* denotes the number of fibroblasts characterized.

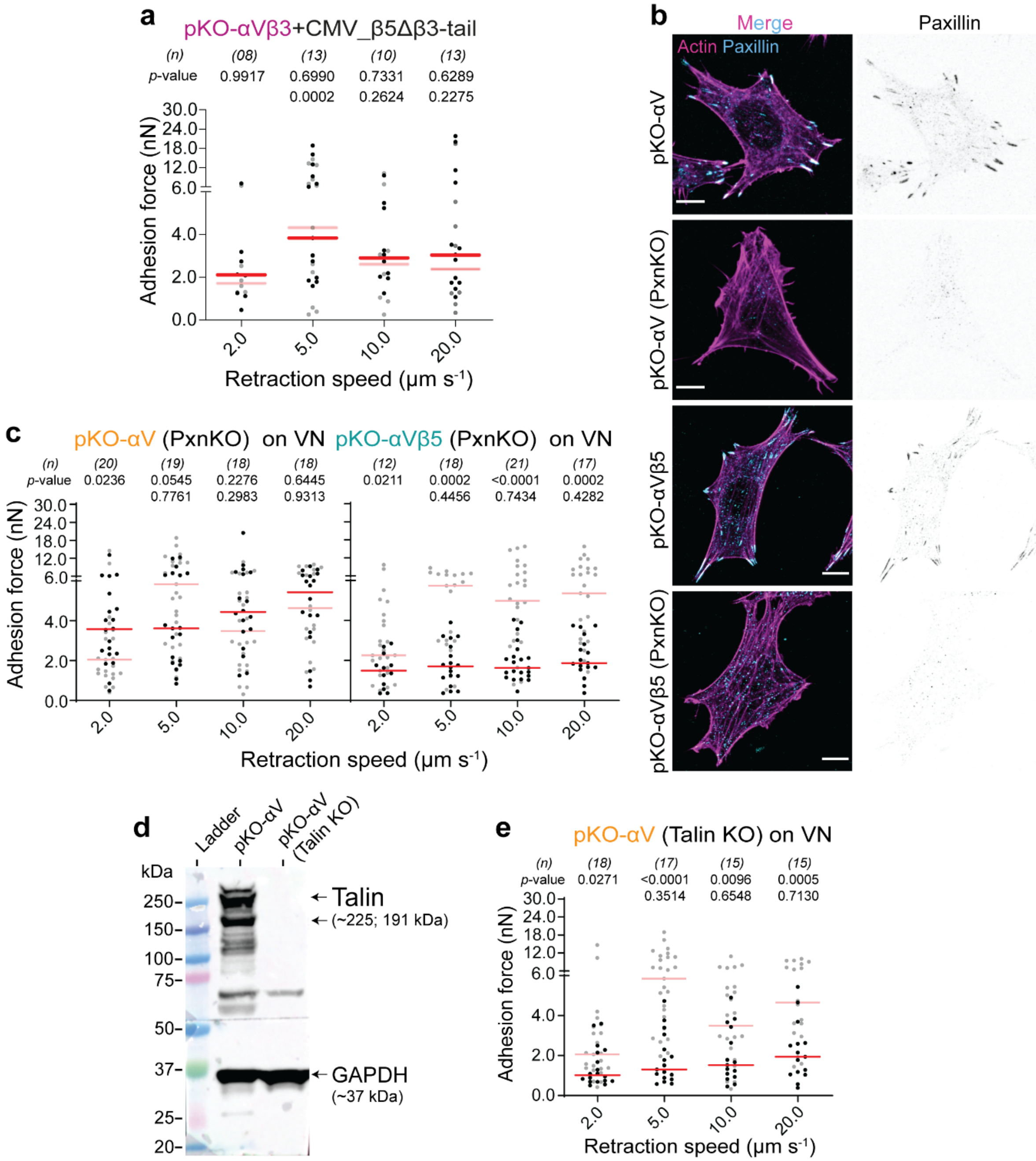

Supplementary Fig. 13. The integrin tail domain and the associated adaptor proteins paxillin and talin influence early cell adhesion to vitronectin under mechanical load. a, The rescue (as per Fig. 2f-i) of the integrin β5-subunit dependent higher cell adhesion force fails if a chimeric β5-subunit[4] (extracellular and transmembrane domain of integrin β5-subunit fused to the cytoplasmic tail of the integrin β3-subunit) is introduced in pKO-αVβ3 fibroblasts. SCFS was conducted at 120 s contact time at which the mechanical-load-dependent biphasic adhesion response of fibroblasts to vitronectin becomes most noticeable (Fig. 1d). The lightened data represent no rescue of adhesion force if pKO-αVβ3 fibroblasts are transfected with an empty vector (control). **b**, Knockout of paxillin (PxnKO) from pKO-αV and pKO-αVβ5 fibroblasts as confirmed *via* immunostaining. The fibroblasts were allowed to spread on vitronectin for 45 min and thereafter stained for paxillin using anti-paxillin antibody (AF488) and for actin using phalloidin-AF647. Scale bars, 10 µm. **c**, Knockout of paxillin from pKO-αV and pKO-αVβ5 fibroblasts

disrupts the biphasic adhesion response upon vitronectin binding for 120 s. *p*-values in the top row compare cell adhesion force on pKO-αV (PxnKO) and pKO-αVβ5 (PxnKO) (black) to pKO-αV and pKO-αVβ5 (lightened) fibroblasts, respectively. **d**, Confirmation of loss of talin in pKO-αV fibroblasts using western blot. GAPDH is used as the loading control. In pKO-αV fibroblasts, talin results in two specific bands of ~225 kDa and ~191 kDa. In pKO-αV (Talin KO) fibroblasts, these two specific bands are not observed, GAPDH results in a specific band at ~37 kDa. **e**, Depleting talin in pKO-αV fibroblasts disrupts the biphasic response upon binding vitronectin for 120 s. *p*-values in the top row compare cell adhesion force of pKO-αV (Talin KO) fibroblasts (black) to pKO-αV fibroblasts (lightened) on vitronectin. *p*-values in the bottom row compare the data for a given retraction speed to that for the preceding retraction speed (*e.g.*, 5 µm s^{-1} with 2 µm s^{-1}). Each dot represents the measurement of a single fibroblast, red bars correspond to the median. *p*-values were obtained using two-tailed Mann-Whitney U-test. *n* denotes the number of fibroblasts characterized on three different experimental days.

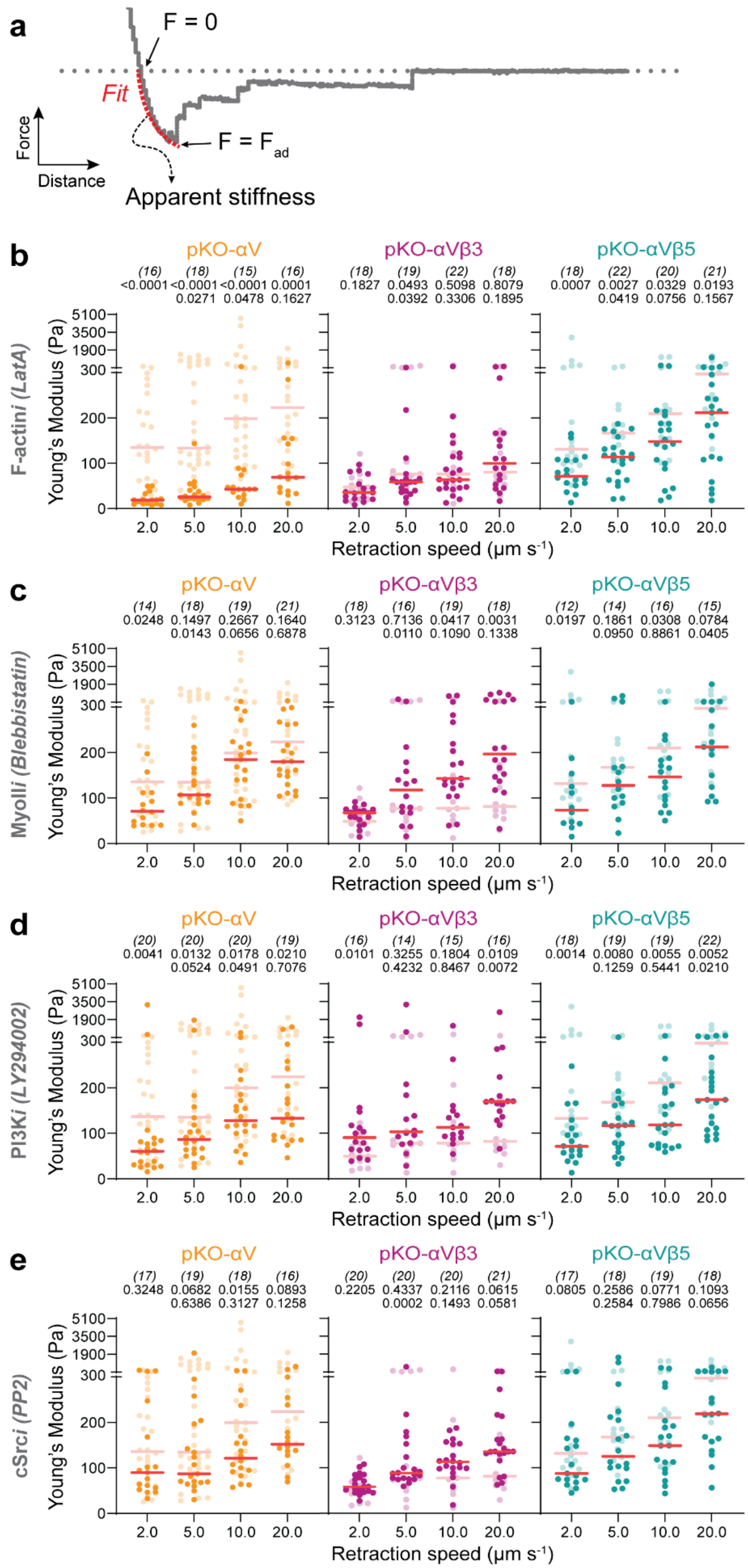

Supplementary Fig. 14. Integrin β-subunits of αV-class integrins are key determinants for cellular

stiffening under mechanical load. a, Representative force-distance curve recorded upon retracting a fibroblast from the substrate using SCFS (compare Supplementary Fig. 2a). To reveal the apparent stiffness of the fibroblast adhering to the substrate for a distinct contact time, the Hertz-Sneddon model is fit to the slope (red dashed line) between the point of inflection ($F = 0$), where the retracting cantilever begins pulling on the cell, and the maximum detachment force of the cell F_{ad}.[5] It has been shown that this fitting procedure estimates the mechanical stiffness of fibroblasts initiating adhesion[5]. **b**-**e**, Retraction speed-dependent apparent cell stiffness (Young's modulus) determined for chemically perturbed pKO-αV, pKO-αVβ3 and pKO-αVβ5 fibroblasts on vitronectin substrate after 120 s of contact. Fibroblasts were treated with 1 µM of F-actin inhibitor LatA (**b**), 20 µM myosin II (Myo II) inhibitor blebbistatin (**c**), 10 µM phosphatidyl inositol 3-kinase (PI3K) inhibitor LY294002 (**d**), or 20 µM cSrc kinase inhibitor PP2 (**e**). Each dot corresponds to one retraction force-distance curve corresponding to SCFS data reported in Supplementary Fig. 12a,b and Fig. 3b,c. Red bars represent median values. *p*-values were obtained using two-tailed Mann-Whitney U-test. *n* denotes the number of fibroblasts characterized. *p*-values in the top row compare the data to the unperturbed (lightened) data. *p*-values in the bottom row compare data for a given retraction speed to the preceding speed (*e.g.*, 5 µm s^{-1} with respect to 2 µm s^{-1}).

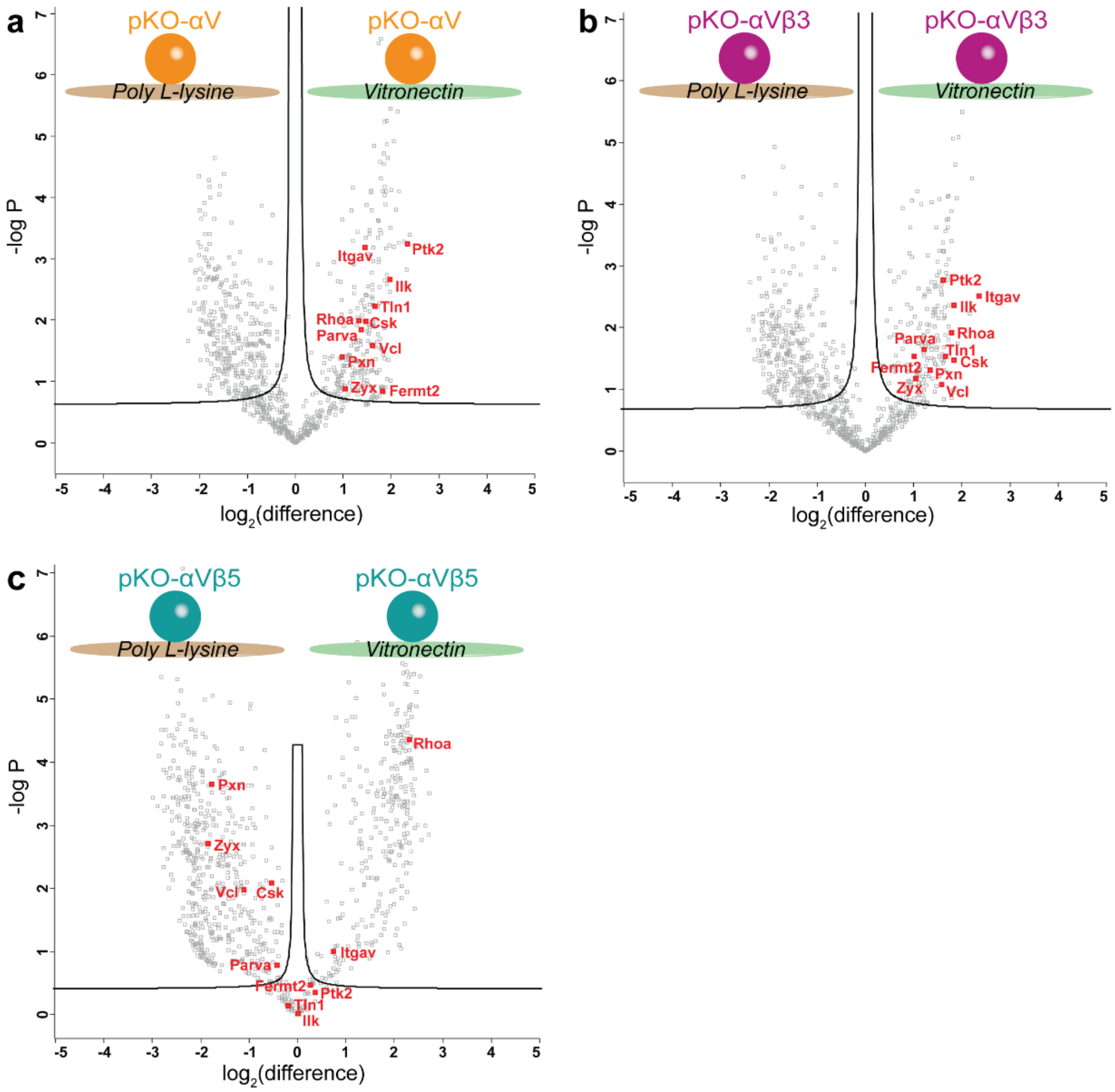

Supplementary Fig. 15. The consensus adhesome composition of different fibroblasts adhering to vitronectin. a-c, Consensus adhesome components highlighted in addition to those displayed in Fig. 5, at adhesion sites isolated from pKO-αV, pKO-αVβ3, and pKO-αVβ5 fibroblasts adhering to vitronectin or poly-L-lysine (control) for 45 min. Mass spectrometry analysis shows the change in protein abundance on vitronectin (+x axis) against poly-L-lysine (-x axis) for (**a**) pKO-αV fibroblasts, (**b**) pKO-αVβ3 fibroblasts, and (**c**) pKO-αVβ5 fibroblasts. The labelled adhesome components in (**a**) pKO-αV fibroblasts are conserved in (**b**) pKO-αVβ3 fibroblasts, though remarkably altered in (**c**) pKO-αVβ5 fibroblasts. Black lines show the boundaries used to identify protein candidates corresponding to a false-discovery rate of 0.1 and a penalty factor for sample-wide standard deviation of 0.1. The data are representative of four independent replicates.

Supplementary Table 1. List of unique peptides detected by mass spectrometry with murine origin corresponding to the complement system proteins and collagens found enriched in plated pKO-αV fibroblasts. Please note that the peptides detected of murine origin are different from the human fibronectin and vitronectin used to plate the fibroblasts. Data taken from Supplementary Fig. 1.

Protein name	Protein ID	Peptide sequence	Start, end position
C3	P01027	IFTVDNNLLPVGK	150, 162
		EYVLPSFEVR	227, 236
		IGQQEVEVK	905, 913
C4b	P01030; P01029	DSSTWLTAFVLK	1072, 1083
		VQYTVCIWR	1464, 1472
Col12a1	E9PX70; Q60847-2;Q60847-5; Q60847	TEFNLNQYYR	190, 199
		DLTADTEYQISVFAMK	393, 408
		FTQVTANSFK	640, 649
		EGTQYDLSVTALYASGAGEALSGK	875, 898
Col18a1	E9QPX1; P39061-2;P39061-1; P39061	GAGLFVGQAGTADPDK	651, 666
		FQGMISELK	667, 675
		TETTGATGQASSLLSGR	1730, 1746
Col1a1	P11087; P11087-2	SAGVSVPGPMGPSGPR	161, 176
		GFPGADGVAGPKGPSGER	483, 500
		GADGSPGKDGAR	741, 752
		GEAGPSGPPGPTGAR	771, 785
Col1a2	Q01149	GEAGAAGPSGPAGPR	700, 714
		TGETGASGPPGFVGEK	837, 852
		GEPGPAGSVGPVGAVGPR	985, 1002
Col3a1	P08121	GGPGGPGLPGPAGK	596, 609

Supplementary References

1. Friedrichs, J., Helenius, J. & Muller, D. J. Quantifying cellular adhesion to extracellular matrix components by single-cell force spectroscopy. *Nat Protoc* **5**, 1353–1361 https://doi.org/10.1038/nprot.2010.89 (2010).
2. Viljoen, A., *et al.* Force spectroscopy of single cells using atomic force microscopy. *Nat Rev Methods Primers* **1**, 63 https://doi.org/10.1038/s43586-021-00062-x (2021).
3. Friedrichs, J., *et al.* A practical guide to quantify cell adhesion using single-cell force spectroscopy. *Methods* **60**, 169–178 https://doi.org/10.1016/j.ymeth.2013.01.006 (2013).
4. Lock, J. G., *et al.* Reticular adhesions are a distinct class of cell-matrix adhesions that mediate attachment during mitosis. *Nat Cell Biol* **20**, 1290–1302 https://doi.org/10.1038/s41556-018-0220-2 (2018).
5. Andreu, I., *et al.* The force loading rate drives cell mechanosensing through both reinforcement and cytoskeletal softening. *Nat Commun* **12**, 4229 https://doi.org/10.1038/s41467-021-24383-3 (2021).
