# Supplementary material for: Load-dependent RGD-context sensing via αV-class integrins reprograms cell adhesion and mechanics
Source: Nat Commun. 2026 Aug 24;17:10137. doi: 10.1038/s41467-026-77028-8 (PMC13601624; doi:10.1038/s41467-026-77028-8)
Supplement: Supplementary file 2 [file 41467_2026_77028_MOESM2_ESM.pdf]

nature portfolio

Corresponding author(s): Daniel J. Müller

Last updated by author(s): Aug 5, 2026

Reporting Summary

Nature Portfolio wishes to improve the reproducibility of the work that we publish. This form provides structure for consistency and transparency in reporting. For further information on Nature Portfolio policies, see our Editorial Policies and the Editorial Policy Checklist.

Statistics

For all statistical analyses, confirm that the following items are present in the figure legend, table legend, main text, or Methods section.

n/a	Confirmed	
☐	☒	The exact sample size (*n*) for each experimental group/condition, given as a discrete number and unit of measurement
☐	☒	A statement on whether measurements were taken from distinct samples or whether the same sample was measured repeatedly
☐	☒	The statistical test(s) used AND whether they are one- or two-sided<br>*Only common tests should be described solely by name; describe more complex techniques in the Methods section.*
☒	☐	A description of all covariates tested
☒	☐	A description of any assumptions or corrections, such as tests of normality and adjustment for multiple comparisons
☐	☒	A full description of the statistical parameters including central tendency (e.g. means) or other basic estimates (e.g. regression coefficient) AND variation (e.g. standard deviation) or associated estimates of uncertainty (e.g. confidence intervals)
☐	☒	For null hypothesis testing, the test statistic (e.g. *F*, *t*, *r*) with confidence intervals, effect sizes, degrees of freedom and *P* value noted<br>*Give P values as exact values whenever suitable.*
☒	☐	For Bayesian analysis, information on the choice of priors and Markov chain Monte Carlo settings
☒	☐	For hierarchical and complex designs, identification of the appropriate level for tests and full reporting of outcomes
☒	☐	Estimates of effect sizes (e.g. Cohen's *d*, Pearson's *r*), indicating how they were calculated

Our web collection on statistics for biologists contains articles on many of the points above.

Software and code

Policy information about availability of computer code

Data collection	SCFS experiments- JPK Cell-Hesion 200 data acquisition software v5.0; Flow Cytometry- BD FACSDiva v8.0 software; FACS- SONY MA900 software v2.0; Brightfield Microscopy- Zeiss (ZenBlack v2012; AxioVision v4.8.2) software; Airyscan microscopy - Zeiss Zen Blue 3.0 software; Mass Spectrometry- Max Quant Software 2.8.1.0, 3D-SIM microscopy using API OMX Blaze - DeltaVision OMX softWoRx v7.0
Data analysis	SCFS experiments- JPK SPM data processing software v8.0.42; Rupture and tether event analysis- Igor Pro 8; Flow Cytometry and FACS- FlowJo v10.10.0 software; Brightfield Microscopy and Airyscan data- Fiji v1.54; Mass Spectrometry- Perseus Software v2.0.6.0

For manuscripts utilizing custom algorithms or software that are central to the research but not yet described in published literature, software must be made available to editors and reviewers. We strongly encourage code deposition in a community repository (e.g. GitHub). See the Nature Portfolio guidelines for submitting code & software for further information.

Data

Policy information about availability of data

All manuscripts must include a data availability statement. This statement should provide the following information, where applicable:

- Accession codes, unique identifiers, or web links for publicly available datasets
- A description of any restrictions on data availability
- For clinical datasets or third party data, please ensure that the statement adheres to our policy

The data that support the findings and the code to analyze the data are available from the corresponding authors upon request.

Research involving human participants, their data, or biological material

Policy information about studies with human participants or human data. See also policy information about sex, gender (identity/presentation), and sexual orientation and race, ethnicity and racism.

Reporting on sex and gender	Not applicable
Reporting on race, ethnicity, or other socially relevant groupings	Not applicable
Population characteristics	Not applicable
Recruitment	Not applicable
Ethics oversight	Not applicable

Note that full information on the approval of the study protocol must also be provided in the manuscript.

Field-specific reporting

Please select the one below that is the best fit for your research. If you are not sure, read the appropriate sections before making your selection.

☒ Life sciences ☐ Behavioural & social sciences ☐ Ecological, evolutionary & environmental sciences

For a reference copy of the document with all sections, see nature.com/documents/nr-reporting-summary-flat.pdf

Life sciences study design

All studies must disclose on these points even when the disclosure is negative.

Sample size	Sample size was not predetermined. The adhesion of at least 10 cells was measured per condition to obtain statistically firm results. We estimated that this number of cells would suffice for biologically relevant differences. For fluorescence intensities, 10'000 cells per sample and a minimum of 2 independent samples were analyzed to attain statistically firm results. Airyscan imaging was performed on at least 15 cells from 2 independent experiments to verify the appearance of phenotypes.
Data exclusions	No analyzed data was excluded.
Replication	Adhesion forces of at least ten cells per condition and contact time were measured on at least three different days. For confocal imaging at least 15 cells in at least two different samples were analyzed. Flow cytometry experiments were performed on at least two samples with each containing 10'000 cells on at least three different days.
Randomization	The order of contact times and retraction speeds was randomized for every cell and condition. Samples used for flow cytometry were analyzed in a random order and in alternation with other samples tested during the same experiment. Samples were allocated randomly into experimental groups on any given day
Blinding	Blinding was not relevant to this study since measurements (cell adhesion/flow cytometry/microscopy) were analyzed after data acquisition and were therefore not susceptible to experimenter bias.

Behavioural & social sciences study design

All studies must disclose on these points even when the disclosure is negative.

Study description	Not applicable
Research sample	Not applicable
Sampling strategy	Not applicable
Data collection	Not applicable
Timing	Not applicable
Data exclusions	Not applicable
Non-participation	Not applicable

Randomization	Not applicable

Ecological, evolutionary & environmental sciences study design

All studies must disclose on these points even when the disclosure is negative.

Study description	Not applicable
Research sample	Not applicable
Sampling strategy	Not applicable
Data collection	Not applicable
Timing and spatial scale	Not applicable
Data exclusions	Not applicable
Reproducibility	Not applicable
Randomization	Not applicable
Blinding	Not applicable

Did the study involve field work? ☐ Yes ☒ No

Reporting for specific materials, systems and methods

We require information from authors about some types of materials, experimental systems and methods used in many studies. Here, indicate whether each material, system or method listed is relevant to your study. If you are not sure if a list item applies to your research, read the appropriate section before selecting a response.

Materials & experimental systems

n/a	Involved in the study	
☐	☒	Antibodies
☐	☒	Eukaryotic cell lines
☒	☐	Palaeontology and archaeology
☒	☐	Animals and other organisms
☒	☐	Clinical data
☒	☐	Dual use research of concern
☒	☐	Plants

Methods

n/a	Involved in the study	
☒	☐	ChIP-seq
☐	☒	Flow cytometry
☒	☐	MRI-based neuroimaging

Antibodies

Antibodies used	Anti-mouse CD61 (integrin β3) (1:200 dilution, Invitrogen, Cat. No. 12-0611-82)<br>Armenian hamster IgG control (1:200 dilution, Invitrogen, Cat. No.12-4888-83)<br>Anti-αVβ5 (1:200 dilution, BD Pharmigen, Cat. No. 565836)<br>Mouse IgGb2(κ) control (1:200 dilution, BD Pharmigen, Cat. No. 565378)<br>Anti-mouse integrin β3 (1:200, EmFret, Cat. No. M031-0)<br>Anti-mouse integrin αV (1:200, Abcam, Cat. No. ab179475)<br>Anti-Paxillin antibody (1:200, Abcam, Cat. No. ab32084)<br>Anti-Talin antibody (1:100, Sigma, Cat. No. T3287)<br>Goat Anti-Mouse AF488 (1:400, Abcam, Cat. No. ab150113)<br>Anti-GAPDH (1:2500, Cell Signaling Technology, Cat. No. 2118S)<br>Goat anti rabbit HRP conjugated secondary antibody (1:2500, BioRad, 170–6515)
Validation	Anti-mouse CD61 (integrin β3) (1:200 dilution, Invitrogen, Cat. No. 12-0611-82) used as per directed by supplier and supported by previous reports provided (https://www.thermofisher.com/antibody/product/CD61-Integrin-beta-3-Antibody-clone-2C9-G3-Monoclonal/12-0611-82#Flow-content)<br>Armenian hamster IgG control (1:200 dilution, Invitrogen, Cat. No.12-4888-83) used as per directed by supplier and supported by previous reports provided (https://www.thermofisher.com/antibody/product/Armenian-Hamster-IgG-clone-eBio299Arm-Monoclonal/12-4888-83)<br>Anti-αVβ5 (1:200 dilution, BD Pharmigen, Cat. No. 565836) used as per directed by supplier and supported by previous reports provided (https://www.bdbiosciences.com/en-us/products/reagents/flow-cytometry-reagents/research-reagents/single-color-

antibodies-ruo/alexa-fluor-647-mouse-anti-integrin-v-5.565836#citations_references)
Mouse IgGb2(κ) control (1:200 dilution, BD Pharmigen, Cat. No. 565378) used as per directed by supplier and supported by previous reports provided (https://www.bdbiosciences.com/en-us/products/reagents/flow-cytometry-reagents/research-reagents/flow-cytometry-controls-and-lysates/alexa-fluor-647-mouse-igg2b-isotype-control.565378#citations_references)
Anti-mouse integrin β3 (1:200, EmFret, Cat. No. M031-0) used as per supplier directions (https://www.emfret.com/fileadmin/user_upload/Datasheets/M031-0_LucH11.pdf)
Anti-mouse integrin αV (1:200, Abcam, Cat. No. ab179475) used as per the supplier suggestions (https://doc.abcam.com/datasheets/active/ab179475/en-us/integrin-alpha-v-antibody-epr16800-ab179475.pdf)
Anti-Paxillin antibody (1:200, Abcam, Cat. No. ab32084) used as per supplier suggestions (https://doc.abcam.com/datasheets/active/ab32084/en-us/paxillin-antibody-y113-ab32084.pdf)
Anti-Talin antibody (1:100, Sigma, Cat. No. T3287) used as per supplier suggestions (https://www.sigmaaldrich.com/specification-sheets/227/151/T3287-BULK_________SIGMA_____.pdf)
Goat Anti-Mouse AF488 (1:400, Abcam, Cat. No. ab150113) used as per supplier suggestions (https://doc.abcam.com/datasheets/active/ab150113/en-us/goat-mouse-igg-h-l-alexa-fluor-488-ab150113.pdf)
Anti-GAPDH (1:2500, Cell Signaling Technology, Cat. No. 2118S) used as per supplier suggestions (https://www.cellsignal.com/products/2118/datasheet?images=1&protocol=0&size=A4)
Goat anti rabbit HRP conjugated secondary antibody (1:2500, BioRad, 170–6515) used as per supplier suggestions (https://www.bio-rad.com/webroot/web/pdf/lsr/literature/LIT418.pdf)

Eukaryotic cell lines

Policy information about cell lines and Sex and Gender in Research

Cell line source(s)

The pKO-αV fibroblast line used in the study was provided by Reinhard Faessler, Max Planck Institute of Biochemistry, Department of Molecular Medicine, 82152 Martinsried, Germany

The Itgb3 and Itgb5 knockout cell lines were generated in house using CRISPR/Cas9 gene editing in the pKO-αV fibroblasts. sgRNAs (with underlined PAM sequences; 5′-NGG-3′) targeted two separate exons [Itgb3 exon 4 (chr11:104,633,595-104,633,614) 5′-ATGTACATGTACGGCGATACAGG-3′; Itgb3 exon 10 (chr11:104,643,724-104,643,743) 5′-CCAGCCCACGCTGCAACAATGGG-3′; Itgb5 exon 3 (chr16:33,865,447-33,865,466) 5′-ACCGTGGATTGCCAAAGTACTGG-3′; Itgb5 exon 4 (chr16:33,876,034-33,876,053) 5′- ACCCAATACACGGATTGGTCTGG -3′] on these genes.

To deplete paxillin in pKO-αV and pKO-αVβ5 fibroblasts CRISPR/Cas9 was employed as described above in case of integrin knockout production. Single-guide RNAs (sgRNAs) for two exons were designed using CHOPCHOP (https://chopchop.cbu.uib.no) for paxillin gene in Mus musculus genome (mm10/GRCm38). The sgRNAs that we designed targeted two separate exons on the paxillin gene. The exon 2 (chr5:115 544 490) [5′- CGTGCCATTGAGGGCCTCGCTGG-3′] and exon 8 (chr5:115 552 099) [5′- GTAAGGTCGTGACCGCCATGGGG-3′] were targeted.

To deplete talin in pKO-αV, we used CRISPR/Cas9 as in the case of integrin knockout production. Single-guide RNAs (sgRNAs) for two exons were designed using CHOPCHOP (https://chopchop.cbu.uib.no) for talin1 and talin2 genes in Mus musculus genome (mm10/GRCm38). The sgRNAs that we designed targeted two separate exons on the talin1 gene where exon 2 (chr4:43,556,642) [5′-GGATCCGCTCACGAATCATGCGG-3′] and exon 46 (chr4:43,535,990) [5′-CGAGCTTGACCACATCAGCGAGG-3′] were targeted. For talin2 gene, where exon 14 (chr9:67,362,656) [5′-ATGGTCGGACAGATGCACCGAGG-3′] and exon 29 (chr9:67,321,836) [5′-AGCTACTTGTAGATTCGGTGAGG-3′] were targeted.

Authentication

All fibroblasts were authenticated by flow cytometry, western blotting or immunostaining.

Mycoplasma contamination

All fibroblasts were tested negative for mycoplasma.

Commonly misidentified lines
(See ICLAC register)

No commonly misidentified cells were used. Cells routinely characterized for their very specific expression patterns of integrins or adhesome proteins.

Palaeontology and Archaeology

Specimen provenance

Not applicable

Specimen deposition

Not applicable

Dating methods

Not applicable

☐ Tick this box to confirm that the raw and calibrated dates are available in the paper or in Supplementary Information.

Ethics oversight

Not applicable

Note that full information on the approval of the study protocol must also be provided in the manuscript.

Animals and other research organisms

Policy information about studies involving animals; ARRIVE guidelines recommended for reporting animal research, and Sex and Gender in Research

Laboratory animals	Not applicable
Wild animals	Not applicable
Reporting on sex	Not applicable
Field-collected samples	Not applicable
Ethics oversight	Not applicable

Note that full information on the approval of the study protocol must also be provided in the manuscript.

Clinical data

Policy information about clinical studies

All manuscripts should comply with the ICMJE guidelines for publication of clinical research and a completed CONSORT checklist must be included with all submissions.

Clinical trial registration	Not applicable
Study protocol	Not applicable
Data collection	Not applicable
Outcomes	Not applicable

Dual use research of concern

Policy information about dual use research of concern

Hazards

Could the accidental, deliberate or reckless misuse of agents or technologies generated in the work, or the application of information presented in the manuscript, pose a threat to:

No	Yes	
☒	☐	Public health
☒	☐	National security
☒	☐	Crops and/or livestock
☒	☐	Ecosystems
☒	☐	Any other significant area

Experiments of concern

Does the work involve any of these experiments of concern:

No	Yes	
☒	☐	Demonstrate how to render a vaccine ineffective
☒	☐	Confer resistance to therapeutically useful antibiotics or antiviral agents
☒	☐	Enhance the virulence of a pathogen or render a nonpathogen virulent
☒	☐	Increase transmissibility of a pathogen
☒	☐	Alter the host range of a pathogen
☒	☐	Enable evasion of diagnostic/detection modalities
☒	☐	Enable the weaponization of a biological agent or toxin
☐	☐	Any other potentially harmful combination of experiments and agents

Plants

Seed stocks: Not applicable

Novel plant genotypes: Not applicable

Authentication: Not applicable

ChIP-seq

Data deposition

☐ Confirm that both raw and final processed data have been deposited in a public database such as GEO.

☐ Confirm that you have deposited or provided access to graph files (e.g. BED files) for the called peaks.

Data access links
May remain private before publication.: Not applicable

Files in database submission: Not applicable

Genome browser session (e.g. UCSC): Not applicable

Methodology

Replicates: Not applicable

Sequencing depth: Not applicable

Antibodies: Not applicable

Peak calling parameters: Not applicable

Data quality: Not applicable

Software: Not applicable

Flow Cytometry

Plots

Confirm that:

☒ The axis labels state the marker and fluorochrome used (e.g. CD4-FITC).

☒ The axis scales are clearly visible. Include numbers along axes only for bottom left plot of group (a 'group' is an analysis of identical markers).

☒ All plots are contour plots with outliers or pseudocolor plots.

☒ A numerical value for number of cells or percentage (with statistics) is provided.

Methodology

Sample preparation: For flow cytometry analysis, fibroblasts were cultured on fibronectin-coated 24 well plates (Greiner) up to ≈ 80% confluency. They were serum deprived for > 4 h in DMEM (31966047, Thermo Fisher Scientific), supplemented with 100 U ml−1 penicillin and 100 µg ml−1 streptomycin (15140122, Thermo Fisher Scientific). Serum-deprived fibroblasts were detached from the plates using 0.25% (w/v) trypsin-EDTA (Gibco #25200056) at 37°C for 2 min. Detached fibroblasts were resuspended in SCFS media with 1% (v/v) FBS and recovered from detachment process at 37°C for at least 30 min. After recovery, cells were pelleted and 105 fibroblasts were resuspended in 100 µl of flow cytometry buffer (2 mM EDTA and 3% (w/v) BSA in PBS) containing fluorophore conjugated primary antibodies as follows; anti-mouse CD61 (integrin β3) (1:200, Invitrogen, #12-0611-82) with Armenian hamster IgG control (1:200, Invitrogen, #12-4888-83), anti-αVβ5 (1:200, BD Pharmigen, #565836) with mouse IgGb2(κ) control (1:200, BD Pharmigen, #565378). The cells were stained with the said antibodies for 30 mins at 4°C in dark conditions. Afterwards, fibroblasts were washed using flow cytometry buffer twice, followed by

measuring their fluorescence intensity employing LSRFortessa (BD AG). Fluorescence activated cell sorting (FACS) was performed using the culture and staining protocols identical to the flow cytometry analysis experiments.

Instrument	BD LSRFortessa; SONY MA900
Software	BD FACSDiva v8.0 software; SONY MA900 software v2.0
Cell population abundance	After excluding debris and doublets 100% of cells were relevant for the study, as we used cell culture condition (100% purity of cells of interest).
Gating strategy	The flow cytometry data was gated according to forward (FSC-A) and side scatter (SSC-A) to exclude debris or dead cells. Doublets were excluded as per forward scatter area (FSC-A) and height (FSC-H) parameters. Sorting gates were created based on the IgG isotype control labelled or unlabelled samples as compared to positive controls.

☐ Tick this box to confirm that a figure exemplifying the gating strategy is provided in the Supplementary Information.

Magnetic resonance imaging

Experimental design

Design type	Not applicable
Design specifications	Not applicable
Behavioral performance measures	Not applicable

Acquisition

Imaging type(s)	Not applicable
Field strength	Not applicable
Sequence & imaging parameters	Not applicable
Area of acquisition	Not applicable

Diffusion MRI ☐ Used ☒ Not used

Preprocessing

Preprocessing software	Not applicable
Normalization	Not applicable
Normalization template	Not applicable
Noise and artifact removal	Not applicable
Volume censoring	Not applicable

Statistical modeling & inference

Model type and settings	Not applicable
Effect(s) tested	Not applicable

Specify type of analysis: ☐ Whole brain ☐ ROI-based ☐ Both

Statistic type for inference (See Eklund et al. 2016)	Not applicable
Correction	Not applicable

Models & analysis

n/a	Involved in the study
☒	☐ Functional and/or effective connectivity
☒	☐ Graph analysis
☒	☐ Multivariate modeling or predictive analysis
