# Supplementary material for: Load-dependent RGD-context sensing via αV-class integrins reprograms cell adhesion and mechanics
Source: Nat Commun. 2026 Aug 24;17:10137. doi: 10.1038/s41467-026-77028-8 (PMC13601624; doi:10.1038/s41467-026-77028-8)
Supplement: Supplementary file 3 [file 41467_2026_77028_MOESM3_ESM.pdf]

nature portfolio

Peer Review File

Load-dependent RGD-context sensing via αV-class integrins reprograms cell adhesion and mechanics

Corresponding Author: Professor Daniel Mueller

This manuscript has been previously reviewed at another journal. This document only contains information relating to versions considered at Nature Communications.

This file contains all reviewer reports in order by version, followed by all author rebuttals in order by version.

Version 0:

Reviewer comments:

Reviewer #1

(Remarks to the Author)
As I mentioned in my previous reviews, the manuscript contains a very thorough characterization of how alphav-mediated adhesions respond to force when bound to vitronectin and fibronectin, and how this depends on different cytoskeletal components and signalling pathways. The measurements are of high quality and rigorous, and the breadth of perturbations employed, both through drugs and elegant cell models expressing specific integrins, is impressive. The combination with proteomics is also very welcome. Given the resubmission to Nature Communications, I do believe the level of novelty and impact is now adequate and I recommend publication. However, in my opinion the authors still have not addressed the main points that I raised in my review for Nature Materials. These points were meant to raise novelty for Nature Materials and they do not compromise the validity of the main results, so this does not change my overall recommendation. That being said, some points should be revised.

In my first comment, I asked for new experiments or theoretical models to address two questions: what mechanisms give rise to the biphasic behaviour, and how it is important to mechanosignalling. Regarding mechanisms, the authors in their response summarize their findings, characterizing how the biphasic behaviour depends on the beta subunits, and the response to the different inhibitions. They then claim that the biphasic behaviour emerges from the catch bond nature of integrin bonds, which is present on vitronectin but not fibronectin (where integrins would form slip bonds). This is problematic at different levels. First and most importantly, the authors do not demonstrate the catch versus slip bond behaviour on vitronectin versus fibronectin (see point below). Second, why should a catch bond lead to a biphasic behaviour of detachment times in response to retraction speeds? A catch bond is defined by a biphasic response of detachment times (or off rates) in response to increasing levels of force, where force remains constant during the pulling. How this translates to a curve where the x axis is not force but retraction speed is not trivial, particularly in an adhesion mediated by multiple bonds. This could still have been discussed with some mathematical arguments/transformations. In any case this is not needed as the catch bond /slip bond behaviour is not demonstrated, but this argument should be removed from the discussion and results.

Regarding the implications in mechanosignalling, all the arguments given by the authors revolve around the adhesion force measurements themselves. However, potential implications in mechanosignalling should be addressed by measuring such signalling, that is, measuring the activation of mechanotransduction pathways. This necessarily involves additional measurements quantifying the outcome of mechanotransduction pathways. As examples, this could be recruitment of focal adhesion components, phosphorylation of molecules like FAK, effects in YAP, or others … Again, I don't think this is necessary at this point, I just mention it to illustrate what I meant.

In another comment, I asked the authors to provide the values of adhesion stiffness in the different conditions. The authors now do provide these values, and I thank them for that. The values change as expected depending on the integrin type engaged and the perturbation employed. I agree with the authors that the trends do not correlate fully with detachment fore measurements, and therefore the force measurements cannot fully be explained by the stiffness. However, it is clear that altered adhesion stiffness will determine how a given retraction speed translates into a loading rate, and this loading rate will in turn affect detachment forces. Ideally, to decouple the effects the authors should calculate loading rates in each curve from

the stiffness values, and then report the detachment forces as a function of loading rates rather than retraction speeds. This could be done by reanalysing the already measured curves. If this is not feasible, the authors should at least clearly state this effect and associated caveats in the results and discussion section.

Finally, I must say that I am puzzled by the authors' response to the issue of catch bonds. I asked how the data in extended figure 9 demonstrated catch bond behaviour. I also asked what type of linear or non-linear relationship between pulling speed and rupture force is expected in catch and slip bonds, and why. The authors now reply that "We expect a catch bond to behave on the pulling speed such as previously proposed where the rupture force of integrins bound to fibronectin does not increase markedly while the retraction speeds double". First, this is a rather vague statement that is hard to verify. Second, why is this expected? This remains unanswered. For a simple slip bond following the bell model, off rates depend exponentially on applied force. From this fundamental relationship, one can mathematically derive the expected force at which a bond would rupture if loaded with a given loading rate (instead of a constant force). The resulting expression predicts that rupture force should scale logarithmically with loading rate, not linearly. If the relationship between off rates and force deviates from the bell model (even if it remains monotonic and thereby slip bond-like) then the force/loading rate relationship could change. So a linear versus non-linear relationship between forces and loading rates (or retraction speeds as experimentally controlled) is not necessarily a sign of catch versus slip bond behaviour. In addition to this, the two linear fits done to curves with 4 points remain inadequate to discriminate linear versus non linear behaviour. I understand that there are experimental limitations, but then the conclusion is that these data cannot be used to infer catch versus slip bond behaviour.

Overall, it is quite frankly surprising to me that such fundamental considerations on how slip and catch bonds work are absent from the manuscript, even after insisting on it two times. At this point, I would simply suggest to remove any claim on catch or slip bonds, and any discussion based on the implications of this.

I was also asked to assess the authors' responses to the comments of reviewer 3. In general, I think the points are addressed, with one exception. Regarding the comments on extended figures 8 and 9, reviewer 3 asked to show an example of a force retraction curve obtained after a very short 50 ms contact. It is not clear to me that this has been done. Then, the reviewer asked to clarify how rupture events were analyzed, and expressed the concern that some measured events could be membrane tethers. In response, the authors state that "The criteria for selecting rupture events have been illustrated in Supplementary Fig. 9a, as frequently described". However, supplementary fig. 9a simply shows an example of a curve, it does not explain any criteria for selecting ruptures. This should be thoroughly explained in the methods.

Open Access This Peer Review File is licensed under a Creative Commons Attribution 4.0 International License, which permits use, sharing, adaptation, distribution and reproduction in any medium or format, as long as you give appropriate credit to the original author(s) and the source, provide a link to the Creative Commons license, and indicate if changes were made.
In cases where reviewers are anonymous, credit should be given to 'Anonymous Referee' and the source.
The images or other third party material in this Peer Review File are included in the article's Creative Commons license, unless indicated otherwise in a credit line to the material. If material is not included in the article's Creative Commons license and your intended use is not permitted by statutory regulation or exceeds the permitted use, you will need to obtain permission directly from the copyright holder.

To view a copy of this license, visit https://creativecommons.org/licenses/by/4.0/

NCOMMS-26-022275-T

Load-dependent RGD-context sensing via αV-class integrins reprograms cell adhesion and mechanics

Point-by-point response to the comments of reviewer #1

Reviewer #1: As I mentioned in my previous reviews, the manuscript contains a very thorough characterization of how alphav-mediated adhesions respond to force when bound to vitronectin and fibronectin, and how this depends on different cytoskeletal components and signalling pathways. The measurements are of high quality and rigorous, and the breadth of perturbations employed, both through drugs and elegant cell models expressing specific integrins, is impressive. The combination with proteomics is also very welcome. Given the resubmission to Nature Communications, I do believe the level of novelty and impact is now adequate and I recommend publication. However, in my opinion the authors still have not addressed the main points that I raised in my review for Nature Materials. These points were meant to raise novelty for Nature Materials and they do not compromise the validity of the main results, so this does not change my overall recommendation. That being said, some points should be revised.

Authors: We sincerely thank Reviewer #1 for the careful re-evaluation of our Manuscript and, in particular, for recommending publication and for recognizing the quality, rigor, and breadth of our characterization of αV-class integrin–mediated adhesion. We are grateful for the reviewer's sustained engagement across the successive rounds of review.

We have taken the reviewer's remaining comments very seriously. In line with the reviewer's clear and repeated guidance, we have removed the catch-bond/slip-bond interpretation and every conclusion built upon it from the Abstract, Introduction, Results, Discussion, and figure legends, while retaining the underlying single-bond and cell-adhesion measurements as purely empirical yet crucial observations. We have additionally added explicit statements and caveats concerning the relationship between adhesion stiffness, loading rate, and detachment force, as the reviewer requested. We detail these revisions point-by-point below and trust that they fully resolve the reviewer's concerns.

Reviewer #1: In my first comment, I asked for new experiments or theoretical models to address two questions: what mechanisms give rise to the biphasic behaviour, and how it is important to mechanosignalling. Regarding mechanisms, the authors in their response summarize their findings, characterizing how the biphasic behaviour depends on the beta subunits, and the response to the different inhibitions. They then claim that the biphasic behaviour emerges from the catch bond nature of integrin bonds, which is present on vitronectin but not fibronectin (where integrins would form slip bonds). This is problematic at different levels. First and most importantly, the authors do not demonstrate the catch versus slip bond behaviour on vitronectin versus fibronectin (see point below). Second, why should a catch bond lead to a biphasic behaviour of detachment times in response to retraction speeds? A catch bond is defined by a biphasic response of detachment times (or off rates) in response to increasing levels of force, where force remains constant during the pulling. How this translates to a curve where the x axis is not force but retraction speed is not trivial, particularly in an adhesion mediated by multiple bonds. This could still have been discussed with some mathematical arguments/transformations. In any case this is not needed as the

catch bond /slip bond behaviour is not demonstrated, but this argument should be removed from the discussion and results.

Authors: We agree with the reviewer and have removed this argument. Specifically, we no longer claim, anywhere in the Manuscript, that the biphasic cell adhesion response emerges from, or is caused by, the catch-bond nature of integrin–vitronectin bonds. We accept the reviewer's central methodological point: a catch bond is defined by a non-monotonic dependence of bond lifetime (or off-rate) on a *constant* applied force, whereas our cell-level measurements report a detachment force as a function of retraction *speed*, and arise from many bonds that are loaded at time-varying rates. Translating the former into the latter is not straightforward and was not formally justified in our Manuscript. We have therefore severed any causal link between single-bond catch/slip behavior and the cellular biphasic response.

The biphasic adhesion response is now described strictly as an empirical, vitronectin- and β-subunit-specific phenomenon. The mechanistic account we retain is the one directly supported by our perturbation data: αVβ3 and αVβ5 integrins differentially require specific actomyosin components (F-actin, myosin II, ROCK, mDia, Arp2/3) and signaling activities (FAK, cSrc, PI3K), with αVβ5 integrin additionally engaging clathrin-mediated endocytosis. These conclusions stand independently of any bond-chemistry interpretation. The corresponding changes have been made in the Abstract, in the Results section 'β3- and β5-subunits modulate integrin-vitronectin bonds', and in the Discussion (see the list of revisions at the end of this response).

Reviewer #1: Regarding the implications in mechanosignalling, all the arguments given by the authors revolve around the adhesion force measurements themselves. However, potential implications in mechanosignalling should be addressed by measuring such signalling, that is, measuring the activation of mechanotransduction pathways. This necessarily involves additional measurements quantifying the outcome of mechanotransduction pathways. As examples, this could be recruitment of focal adhesion components, phosphorylation of molecules like FAK, effects in YAP, or others … Again, I don't think this is necessary at this point, I just mention it to illustrate what I meant.

Authors: We thank the reviewer for clarifying this point, which we understand is illustrative and not a request for further experiments at this stage. We fully agree that establishing the force-induced *activation* of a mechanotransduction pathway would require direct biochemical or imaging readouts, such as FAK phosphorylation, YAP nuclear translocation, or the recruitment of adhesion components under load, and that our small-molecule perturbations report the *requirement* of a given pathway for the adhesion response under mechanical load, rather than its activation by force. Consistent with the reviewer's earlier comment, we had already replaced "trigger" with "employ" wherever causation was implied; we have now gone further and removed any remaining wording that could be read as a claim of force-induced pathway activation from the Results and Discussion, and we frame these as open questions for future study. We are grateful that the reviewer considers the additional measurements unnecessary for the present work.

Reviewer #1: In another comment, I asked the authors to provide the values of adhesion stiffness in the different conditions. The authors now do provide these values, and I thank them for that. The values change as expected depending on the integrin type engaged and the

perturbation employed. I agree with the authors that the trends do not correlate fully with detachment fore measurements, and therefore the force measurements cannot fully be explained by the stiffness. However, it is clear that altered adhesion stiffness will determine how a given retraction speed translates into a loading rate, and this loading rate will in turn affect detachment forces. Ideally, to decouple the effects the authors should calculate loading rates in each curve from the stiffness values, and then report the detachment forces as a function of loading rates rather than retraction speeds. This could be done by reanalysing the already measured curves. If this is not feasible, the authors should at least clearly state this effect and associated caveats in the results and discussion section.

Authors: Thank you, we agree that adhesion stiffness co-determines the loading rate experienced at a given retraction speed and thereby influences the measured detachment forces. We carefully considered recomputing whole-cell detachment forces as a function of loading rate. We concluded, however, that a single loading rate cannot be uniquely assigned to a whole-cell detachment-force value: the rate at which the adhesion is loaded is set by the series compliance of cantilever and cell, varies over the course of a single retraction, and differs among the many bonds that together produce the whole-cell detachment force. A whole-cell "force versus loading rate" plot would therefore rest on an effective loading rate that is itself model-dependent and not directly measured. Moreover, as the reviewer rightly notes in the comment below, even a well-defined loading-rate axis would not by itself discriminate bond chemistry. For these reasons, and following the reviewer's explicit alternative, we have chosen to state the effect and its caveats clearly rather than to present a derived force-versus-loading-rate analysis that we could not robustly justify.

Concretely, we have: (i) retained the single-bond loading-rate analysis in Supplementary Fig. 10, which reports the most probable loading rates experienced by individual integrin–vitronectin bonds at each retraction speed; and (ii) added explicit statements to the Results (sections 'β3- and β5-subunits modulate integrin-vitronectin bonds' and 'Vitronectin-bound αV-class integrins modulate cellular stiffness') and to the Discussion making clear that the retraction speed is the experimentally controlled variable; that differences in adhesion stiffness across conditions translate a given retraction speed into different loading rates; that the measured detachment forces therefore reflects the contribution from bond-level properties and from the stiffness-dependent loading rate, which we do not attempt to disentangle; and that, as the reviewer observed, the measured stiffness trends do not fully correlate with the detachment-force trends, so adhesion stiffness alone does not account for the adhesion measurements.

Reviewer #1: Finally, I must say that I am puzzled by the authors' response to the issue of catch bonds. I asked how the data in extended figure 9 demonstrated catch bond behaviour. I also asked what type of linear or non-linear relationship between pulling speed and rupture force is expected in catch and slip bonds, and why. The authors now reply that "We expect a catch bond to behave on the pulling speed such as previously proposed where the rupture force of integrins bound to fibronectin does not increase markedly while the retraction speeds double". First, this is a rather vague statement that is hard to verify. Second, why is this expected? This remains unanswered. For a simple slip bond following the bell model, off rates depend exponentially on applied force. From this fundamental relationship, one can mathematically derive the expected force at which a bond would rupture if loaded with a given loading rate (instead of a constant force). The resulting expression predicts that rupture force should scale logarithmically with loading rate, not linearly. If the relationship between off rates and force deviates from the bell model (even if it remains monotonic and thereby slip bond-like) then the force/loading rate relationship could change. So a linear versus non-linear relationship between forces and loading rates (or retraction speeds as experimentally controlled) is not necessarily a sign of catch versus slip bond behaviour. In addition to this,

the two linear fits done to curves with 4 points remain inadequate to discriminate linear versus non linear behaviour. I understand that there are experimental limitations, but then the conclusion is that these data cannot be used to infer catch versus slip bond behaviour.

Authors: We thank the reviewer and we concede this point in full. We agree that, for a slip bond obeying the Bell model — where the off-rate increases exponentially with applied force — the framework of dynamic force spectroscopy predicts the most probable rupture force to scale approximately *logarithmically* with loading rate, not linearly, and that deviations of the rupture-force-versus-retraction-speed relationship from linearity are therefore not, in themselves, diagnostic of catch- versus slip-bond behavior. This is all the more true for an adhesion comprising multiple bonds loaded at a non-uniquely-defined rate. We also agree that fitting two straight lines to a four-point dataset (the overlapping triples at 2, 5, 10 µm s^{-1} and 5, 10, 20 µm s^{-1}) cannot reliably discriminate linear from non-linear behavior.

Accordingly, we have removed the catch-/slip-bond interpretation and the associated linear fits from Supplementary Fig. 9 and its legend, and we no longer draw any inference about bond chemistry from these data. We now present Supplementary Fig. 9 solely as the measured dependence of single integrin–vitronectin bond rupture force on retraction speed, describing this dependence as non-linear (for αVβ3-containing fibroblasts) or approximately linear (for pKO-αVβ5 fibroblasts) without assigning a bond mechanism. We have correspondingly revised the Abstract, Results, and Discussion. We have retained the recent structural-biology references on force-mediated conformational changes of αVβ3 integrin (hybrid-domain swing-out) introduced at Reviewer #3's request, but we no longer present these as evidence that our own data demonstrate a catch bond.

Reviewer #1: Overall, it is quite frankly surprising to me that such fundamental considerations on how slip and catch bonds work are absent from the manuscript, even after insisting on it two times. At this point, I would simply suggest to remove any claim on catch or slip bonds, and any discussion based on the implications of this.

Authors: We have followed this recommendation without reservation. All claims regarding catch or slip bonds, and all discussion built on their implications, have been removed throughout the Manuscript — from the Abstract, Introduction, Results, Discussion, and the Supplementary Fig. 9 legend, as well as from the interpretive schematic. We regret that our earlier responses did not adequately engage with the theoretical considerations the reviewer raised, and we are grateful for the reviewer's persistence, which has improved the precision of the Manuscript. We emphasize that none of our empirical findings depend on the removed interpretation: the vitronectin- and β-subunit-specific biphasic cell adhesion response, the differential requirements for actomyosin and signaling pathways, the αVβ5 integrin–specific role of clathrin-mediated endocytosis, the integrin β-subunit–dependent regulation of cell stiffness, and the αVβ3 integrin–dominated long-term adhesome all remain fully intact and are now presented without the contested mechanistic framing. We thank the reviewer once again for recommending publication.

Reviewer #1: I was also asked to assess the authors' responses to the comments of reviewer 3. In general, I think the points are addressed, with one exception. Regarding the comments on extended figures 8 and 9, reviewer 3 asked to show an example of a force retraction curve obtained after a very short 50 ms contact. It is not clear to me that this has been done. Then,

the reviewer asked to clarify how rupture events were analyzed, and expressed the concern that some measured events could be membrane tethers. In response, the authors state that "The criteria for selecting rupture events have been illustrated in Supplementary Fig. 9a, as frequently described". However, supplementary fig. 9a simply shows an example of a curve, it does not explain any criteria for selecting ruptures. This should be thoroughly explained in the methods.

Authors: We have followed the reviewers suggestion "*to show an example of a force retraction curve obtained after a very short 50 ms contact*", which has now been added to the Supplementary Fig. 8 in the Manuscript and shown in Fig. R1 below.

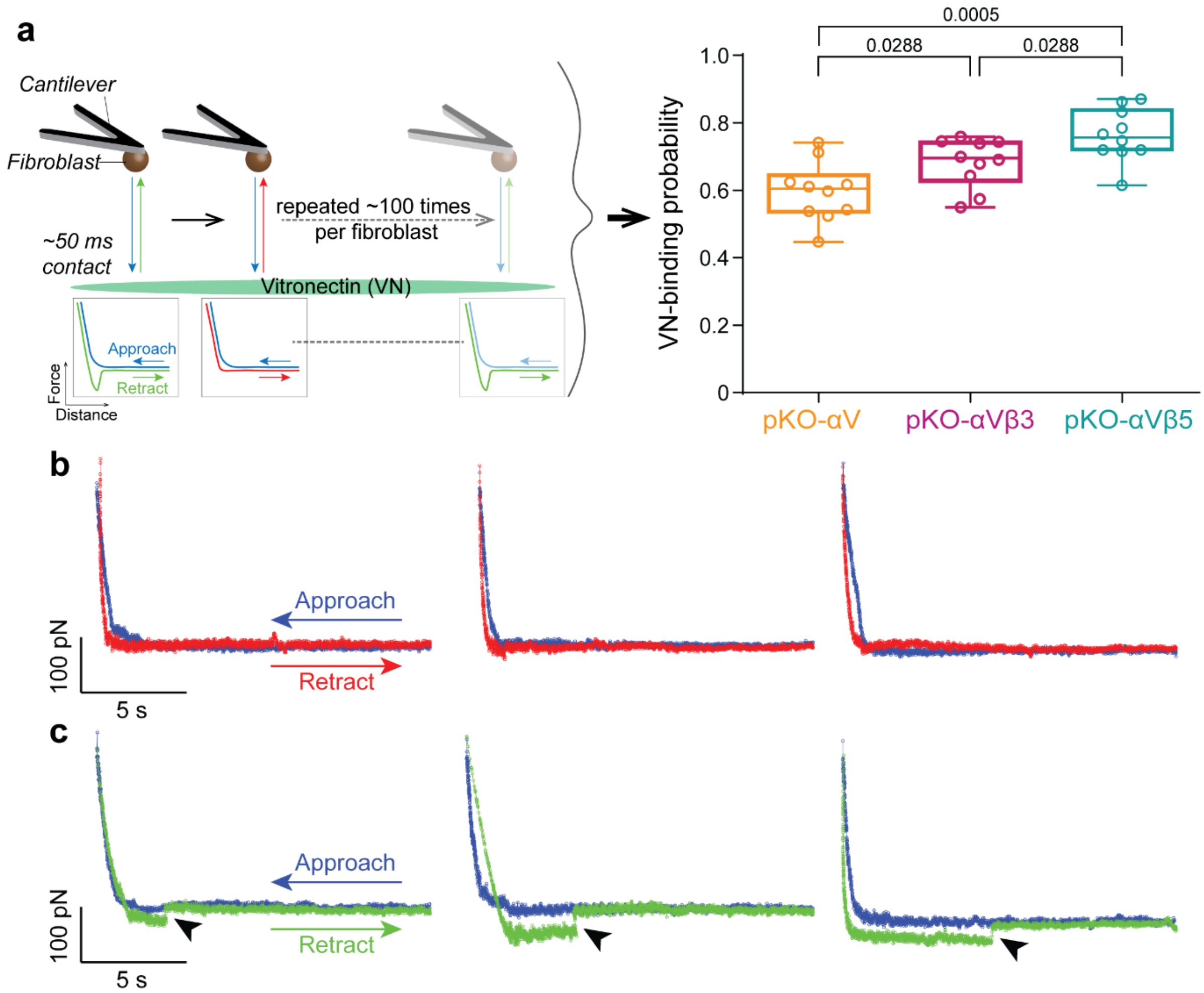

Fig. R1, included as Supplementary Fig. 8 in the revised Manuscript. Binding probability of αV-class integrins to vitronectin. a, Vitronectin-binding probability of pKO-αV, pKO-αVβ3 and pKO-αVβ5 fibroblasts as measured by repeatedly approaching and retracting a single fibroblast to a vitronectin (VN)-coated substrate at 1 µm s^{-1} velocity and ~50 ms contact time (Methods). Each data point represents an outcome from ~100 approach-retraction cycles per fibroblast collected for 10 different cells. Box plots represent median and interquartile range. *p*-values were obtained using Mann-Whitney U-test. **b**, Three example force-time curves representing approach and retract when no unbinding event between fibroblast and substrate is recorded. **c**, Three example force-time curves representing approach and retract when an unbinding event (black arrow heads) between fibroblast and substrate is recorded.

To address the Reviewer's next requirement to "*clarify how rupture events were analyzed*", we have added the following clarification to the Methods section '*Analysis of single rupture events from SCFS'* section in the Manuscript: "*Briefly, to avoid any contribution of (un-)binding events*

related to membrane tethers, we analyzed only rupture events up to cell-substrate retraction distances of 10 µm beyond which the connection between the adhesion assemblies and actomyosin cortex is expected to be lost, and membrane tether events are expected to occur more frequently[1,2]." This statement encompasses and elaborates what has been shown in Supplementary Fig. 9a of the Manuscript.

Summary of textual revisions made in response to Reviewer #1

For the reviewer's convenience, the principal changes are listed below. All are highlighted in the revised Manuscript.

- Abstract — the phrase describing "the catch-bond–forming αVβ3 and the slip-bond–forming αVβ5 integrins" has been reworded to refer to αVβ3 and αVβ5 integrins taking complementary, load-dependent roles, with no reference to bond chemistry.
- Results, 'β3- and β5-subunits modulate integrin-vitronectin bonds' — the sentence stating that the non-linear rupture force "indicat[es] … a catch bond behavior … [that] extends previous studies … thus suggesting catch bonds being common amongst integrin family members" has been removed. The data are now described as a non-linear (αVβ3) or approximately linear (αVβ5) dependence of single-bond rupture force on retraction speed, with an added caveat on the role of adhesion stiffness and loading rate.
- Discussion — the statements linking the single-bond catch/slip behavior to the biphasic cell adhesion response have been removed; the loading-rate/stiffness caveat has been added.
- Supplementary Fig. 9 and legend — the two linear fits and the "catch bond-like / slip bond-like" descriptors have been removed; the legend now reports the measured speed-dependence of rupture force without assigning a bond mechanism.
- Introduction — the structural-biology references on force-mediated αVβ3 conformational change (hybrid-domain swing-out) are retained, but no longer framed as support for a catch-bond claim in our data.
- Throughout — wording implying force-induced pathway activation has been tempered to reflect that the inhibitor experiments report pathway requirement, not activation.

References

1. te Riet, J., *et al.* Dynamic coupling of ALCAM to the actin cortex strengthens cell adhesion to CD6. *J Cell Sci* **127**, 1595–1606 https://doi.org/10.1242/jcs.141077 (2014).
2. Friedrichs, J., Helenius, J. & Muller, D. J. Quantifying cellular adhesion to extracellular matrix components by single-cell force spectroscopy. *Nat Protoc* **5**, 1353–1361 https://doi.org/10.1038/nprot.2010.89 (2010).
